# Obesity Increases Atherosclerosis Susceptibility via an Intertissue miR-30e-SLC7A11 Axis

**DOI:** 10.1161/CIRCRESAHA.126.328493

**Published:** 2026-09-11

**Authors:** Chen Wang, Jianqing She, Xiaoli Qian, Haoyu Wu, Wenyang Hao, Xiao Liang, Ning Guo, Xulei Dai, Fangzhou He, Songyang He, Jin Zhang, Yuyang Lei, Xiaozhen Zhuo, Hongbing Li, Yongbai Luo, Kai Deng, Yi Liu, Shanshan Gao, Xiao Yuan, Hui Liu, Ting Bai, Ying Xiong, John Y-J. Shyy, Zu-Yi Yuan

**Affiliations:** 1 Department of Cardiology (C.W., J.S., H.W., X.L., N.G., S.H., J.Z., Y. Lei, X.Z., H. Li, Y. Luo, S.G., X.Y., T.B., Y.X., Z.-Y.Y.), The First Affiliated Hospital of Xi’an Jiaotong University, China.; 2 Department of Clinical Laboratory (K.D., Y. Liu), The First Affiliated Hospital of Xi’an Jiaotong University, China.; 3 Cardiovascular Research Center, School of Basic Medical Sciences, Xi’an Jiaotong University Health Science Center, China (X.Q., W.H., F.H.).; 4 Division of Cardiology, Department of Medicine, University of California, San Diego, La Jolla (J.Y.-J.S.).; 5 Department of Laboratory Animal Science, School of Basic Medical Sciences, Xi’an Jiaotong University, China (X.D.).; 6 BioBank, The First Affiliated Hospital of Xi’an Jiaotong University, China (H. Liu).

**Keywords:** atherosclerosis, endothelial cell, exosomes, microRNAs, obesity, vascular diseases

## Abstract

**BACKGROUND:**

With obesity as a risk factor for atherosclerotic disease, recent research suggests that adipose tissue in obese animal models and humans can generate endocrine-like molecules that affect arterial health. Previous studies showed that microRNA-30e (miR-30e) levels are elevated in atherosclerosis and solute carrier family 7 member 11 (SLC7A11), a cystine/glutamate transporter, is involved in atherogenesis. However, whether an endocrine-like link between the adipose-derived miR-30e-5p and SLC7A11 in the vascular endothelium can lead to obesity-caused atherosclerosis is unknown.

**METHODS:**

miRNA data mining and reverse transcription-polymerase chain reaction validations were used to demonstrate the positive association among serum levels of miR-30e-5p, obesity, and coronary arterial disease in human patients. Transcriptomics (bulk RNA sequencing and single-nucleus RNA sequencing), metabolomics, and in silico analysis were used to establish a miR-30e-5p–SLC7A11 regulation of central carbon metabolism, mitochondrial and endothelial cell (EC) function. Mouse models with EC-specific *Slc7a11* knockout (EC-*Slc7a11*^−/−^) and gain- or loss-of- function of miR-30e-5p were used to elucidate the detrimental role of this endocrine-like axis in obesity-related atherosclerosis.

**RESULTS:**

The level of adipocyte-derived miR-30e-5p was significantly upregulated in obese human patients with coronary artery disease and in obese and atherosclerotic mice. Mediated through serum exosomes, the adipocyte-generated miR-30e-5p targeted *SLC7A11* mRNA in vascular ECs. SLC7A11 deficiency due to miR-30e-5p targeting dysregulated glutamate/cystine metabolism, increased glycolysis, reduced oxidative phosphorylation, and impaired mitochondrial function. The EC dysfunction could be rectified by SLC7A11 overexpression or miR-30e-5p antagonism. Administration of exogenous miR-30e-5p or white adipose tissue-derived exosomes phenocopied the increased atherosclerosis in EC-*Slc7a11*^−/−^ mice. In contrast, miR-30e-5p antagomir or GW4869 treatment reduced atherosclerosis in *Apoe*^−/−^ and *ob/ob* mice.

**CONCLUSIONS:**

Our multiomics approaches demonstrate that the adipose-derived miR-30e-5p downregulated *SLC7A11* mRNA in ECs via tissue crosstalk. The resulting EC dysfunction led to obesity-related atherosclerosis in mice. These findings underscore a causality between obesity and atherosclerosis in the context of cardiovascular-kidney-metabolic syndrome.

**Novelty and Significance**

**What Is Known?**

- Obesity is a risk factor for atherosclerosis, and dysfunctional white adipose tissue releases exosomal miRNAs for interorgan communication. A higher serum level of microRNA-30e (
  miR-30e)-5p is present in subjects with atherosclerosis, likely due to secretion by adipose tissues.
- Solute carrier family 7 member 11 (SLC7A11), a cystine-glutamate antiporter, regulates cystine uptake and glutathione synthesis to maintain cellular redox balance and defend against oxidative stress and ferroptosis.
- Although the cardiovascular-kidney-metabolic syndrome links obesity to vascular diseases, the adipose-miR-30e-5p-SLC7A11 axis linking obesity, endothelial dysfunction, and atherogenesis remains to be investigated.

**What New Information Does This Article Contribute?**

- Circulating and adipose-derived exosomal miR-30e-5p are elevated in obese individuals with coronary artery disease, which is correlated with metabolic risk factors.
- Adipocyte-secreted miR-30e-5p transfers to vascular endothelial cells via exosomes, and miR-30e-5p in endothelial cells targets SLC7A11 to cause endothelial cell dysfunction.
- Endothelial-specific *Slc7a11* knockout exacerbates atherosclerosis in mice. Overexpression of adipose-derived miR-30e-5p or administration of adipose exosomes increases atherogenesis. By contrast, miR-30e-5p inhibition or blockade of exosome secretion restores SLC7A11 expression, thus alleviating atherosclerosis.

Obesity is associated with metabolic dysfunction and an increased risk of atherosclerosis. We showed that miR-30e-5p is increased in atherosclerosis. miR-30e-5p is released from adipose tissue and delivered via exosomes to vascular endothelial cells, where miR-30e-5p targets SLC7A11. SLC7A11 downregulation disrupts glutamate/cystine metabolism, impairs mitochondria, and drives endothelial dysfunction, ultimately exacerbating atherosclerosis. This study identifies an endocrine miR-30e-5p–SLC7A11 axis linking obesity to atherosclerosis.

**Meet the First Author, see p e000771**

Atherosclerosis is a multifaceted disease that involves chronic inflammation and maladapted metabolism in several tissue types.^1–3^ Clinical guidance suggests that patients with metabolic syndrome are at greatly increased risk of atherosclerotic cardiovascular disease.^4,5^ The novel concept of cardiovascular-kidney-metabolic syndrome has brought further attention to the causation between obesity and atherosclerosis.^6^ Despite ample clinical evidence revealing the association between metabolically impaired adipose tissue and atherosclerotic vasculature, the underlying mechanism remains unclear.

Knowledge gained from multiple studies shows a strong association between visceral adipose tissue and atherosclerotic outcomes.^7,8^ With obesity, impaired differentiation of adipocytes reduces the production of adiponectin but increases the secretion of proinflammatory cytokines.^9^ The prevalent hypothesis of atherosclerosis states that endothelial injury aggravates vascular inflammation and lipid uptake, which initiates atherosclerosis.^10,11^ Because adipocytes are an abundant tissue origin of exosome-carried microRNAs (miRNAs), emerging evidence suggests that adipocyte-derived exosomes can regulate vascular function and disease in an endocrine-like manner.^12,13^ However, it is unknown whether any exosome-associated miRNA can function as a messenger between adipose tissue and vascular endothelium to affect atherosclerotic cardiovascular disease. The p53-induced microRNA-30e (miR-30e) not only functions as a tumor suppressor in multiple cancer types but also is linked to atherosclerotic cardiovascular disease in humans and rodents.^14–20^ miR-30e is primarily released from adipose tissue in an exosome-dependent manner.^12^ However, whether adipose-derived miR-30e affects the endothelium to exacerbate atherosclerosis remains unclear.

Expressed on the cell membrane, solute carrier family 7 member 11 (SLC7A11) is an amino acid transporter with its main function being the promotion of cystine uptake and glutathione biosynthesis, which protect cells against oxidative stress and ferroptosis.^21,22^ As a ferroptosis marker, SLC7A11 plays critical roles in glutamine-glutathione metabolism and thus is involved in the pathogenesis of cancer and atherosclerosis.^23,24^ Obese rats fed a high-fat diet (HFD) show lower levels of Slc7a11, which was associated with increased serum tumor necrosis factor-α (TNF-α) levels.^25^ Given that endothelial cell (EC) dysfunction with an exacerbated redox state causes clinical and experimental atherosclerosis, the impaired SLC7A11-glutathione axis may be involved in atherosclerotic cardiovascular disease.^26,27^

Notably, *SLC7A11* mRNA is a putative target of miR-30, which prompted us to investigate whether adipocyte-derived miR-30e can target *SLC7A11* in ECs in an exosome-dependent manner.^28^ If true, this endocrine-like mechanism may mediate, in part, obese-aggravated atherosclerosis. Our results show elevated levels of miR-30e-5p in adipose-derived exosomes and serum from obese individuals with coronary artery disease (CAD), which was associated with atherosclerosis progression. Moreover, adipose-originated miR-30e-5p could downregulate SLC7A11 and mitochondrial function in ECs. These findings provide new insights into adipocyte-EC crosstalk and their implications for miR-30e antagonism to ameliorate obesity-enhanced atherosclerotic cardiovascular disease.

## Methods

### Data Availability

The data that support the findings of this study are available from the corresponding author upon request. The RNA sequencing and single-nucleus RNA sequencing data sets produced from this study are available in the following databases: Gene Expression Omnibus (GSE334495) and GSE335161. A detailed description of the materials and methods is provided in the Supplemental Material. All clinical samples were obtained at the First Affiliated Hospital of Xi’an Jiaotong University with informed consent and institutional ethics committee approval (No. LLSBPJ-2024-483). Animal experiments were approved by the Institutional Animal Ethics Committee of Xi’an Jiaotong University (No. 2022-170).

## Results

### Elevated Level of Adipose-Derived miR-30e-5p in Obese Individuals With CAD

We initially mined published Gene Expression Omnibus data sets to identify putative miRNAs that link obesity and atherosclerosis. The level of miR-30e-5p was significantly increased in serum from patients with atherosclerosis (GSE96621) and circulating miRNA from HFD-fed mice.^29^ However, the miR-30e-5p level was lower in serum exosomes from ADicer knockout mice in which miRNA processing is hindered due to Dicer specific ablation in adipose tissue (Figure 1A).^12^ Reanalyzing the GSE25470 data set, we found that the level of miR-30e-5p was also increased in white adipose tissue (WAT) from obese patients (Figure 1B). To further investigate the causality between obesity, circulating miR-30e-5p, and atherosclerotic disease, we collected serum from patients with CAD and healthy controls (demographics in Table S3). In agreement with in silico analyses of data by others (Figure 1A and 1B), patients with CAD registered in this study had higher levels of miR-30e-5p than healthy controls (Figure 1C). The receiver operating characteristic curve revealed that patients would have a higher probability of CAD if their miR-30e-5p level was 43.53% higher than that of the healthy controls, with a sensitivity of 68.24% and specificity of 75.29% (area under the receiver operating characteristic curve, 0.80; Figure 1D). We further classified patients with CAD into 3 groups according to their body mass index (BMI): normal weight (18.5 ≤ BMI ≤24.9 kg/m^2^), overweight (25–29.9 kg/m^2^), and obese (≥30 kg/m^2^). Serum levels of miR-30e-5p were elevated more in obese individuals with CAD (Figure 1E). Furthermore, we initially observed positive correlations of circulatory levels of miR-30e-5p with BMI, triglycerides, and low-density lipoprotein cholesterol (LDL-C) level, but a negative correlation with high-density lipoprotein cholesterol level in the cohort (Figure S1A). After outlier exclusion, only BMI and LDL-C were significantly correlated with miR-30e-5p level, while triglycerides showed a marginal correlation (*P*=0.089) and high-density lipoprotein cholesterol lacked statistical correlation (Figure 1F). Exosomes were then isolated from adipose tissues and serum of obese/CAD individuals as well as nondiseased controls for further analysis (demographics in Table S4). On nanoparticle tracking analysis, these isolated exosomes had the typical size (≈100 nm; Figure 1G), and electron microscopy revealed the cup-shaped morphology (Figure 1H). The 2 groups of exosomes were then labeled with PKH67 and incubated with human aortic ECs (HAECs) in vitro (Figure 1I). The level of miR-30e-5p was increased in HAECs cocultured with exosomes from adipose tissues or serum of obese/CAD patients (Figure 1J). In line with data from human patients, miR-30e-5p levels were increased in WAT, serum exosomes, and aortic tissues of *ob/ob* mice, *Apoe*^−/−^ mice fed an HFD, and C57BL/6 mice administered adeno-associated virus (AAV)8-*PCSK9*^*D377Y*^ and fed an HFD (Figure 1K through 1M). However, the miR-30e-5p level was not elevated in brown adipose tissue of these 3 mouse models (Figure 1K through 1M). Collectively, results in Figure 1 suggest that the WAT-derived exosomes might transport miR-30e-5p from adipocytes to vascular endothelium in obese humans with CAD, obese mice, and atherosclerotic mice.

**Figure 1.**
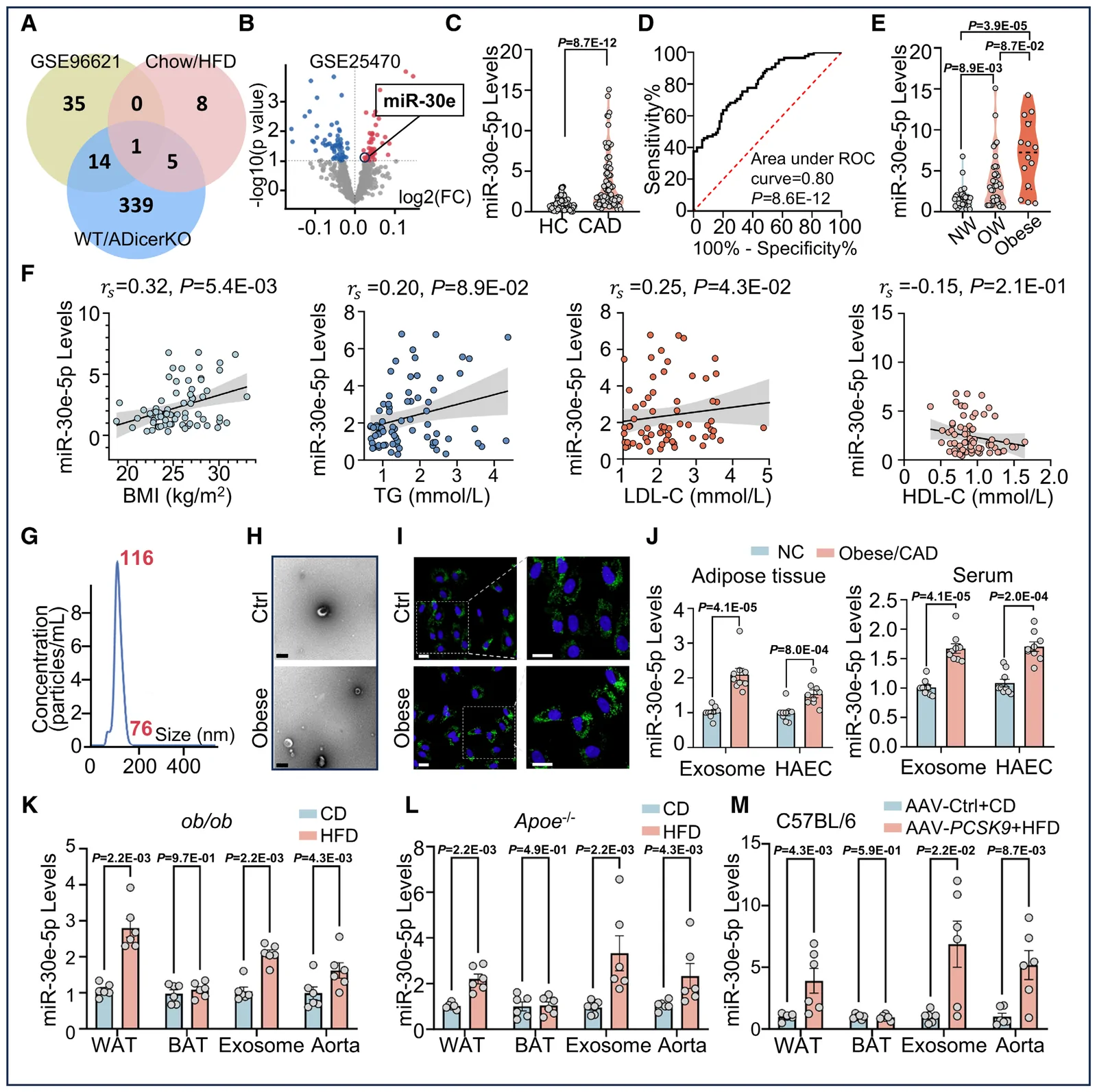
**Elevated levels of adipose-derived microRNA-30e (miR-30e)-5p in obese individuals with coronary artery disease (CAD). A**, Venn diagram demonstrating the intersection of miRNA levels among 3 data sets: miR-30e levels were increased in serum from atherosclerotic human patients (GSE96621, chrome yellow) and high-fat diet (HFD)–fed mice (pink) but decreased in serum exosomes from fat-specific Dicer (ADicer)-knockout mice (blue). **B**, Volcano plot showing increased levels of miR-30e in white adipose tissue (WAT) from obese patients (n=30) and normal controls (NCs, n=26; GSE25470). **C**, Serum was collected from patients with CAD (n=85) and healthy controls (HCs; n=85) registered at the First Affiliated Hospital of Xi’an Jiaotong University. Serum levels of miR-30e-5p were measured by quantitative real‑time polymerase chain reaction (qPCR). The data are fold-change normalized to the mean level of HCs. **D**, Receiver operating characteristic (ROC) curve with sensitivity and specificity of serum levels of miR-30e-5p for differentiating patients with CAD from HCs at diagnosis. **E**, Serum levels of miR-30e-5p in patients with CAD and normal weight (NW; 18.5 ≤ body mass index [BMI] ≤24.9 kg/m^2^, n=33), overweight (OE; 25–29.9 kg/m^2^, n=38), and obesity (≥30 kg/m^2^, n=14). The data are fold-change normalized to the mean of patients with NW. **F**, Correlations between miR-30e-5p levels and BMI, triglycerides (TG), low-density lipoprotein cholesterol (LDL-C), and high-density lipoprotein cholesterol (HDL-C) in patients with CAD. **G**, Size distribution of serum exosomes assessed by nanoparticle tracking analysis. **H**, Electron microscopy images of adipose tissue-derived exosomes, ≈100 nm in diameter. Scale bar: 100 nm. **I**, Serum exosomes were labeled with PKH67 green fluorescence dye, incubated with human aortic endothelial cells (HAECs) for 24 h, and then fixed for confocal imaging. Nuclei were counterstained with DAPI (4′,6‑diamidino‑2‑phenylindole; blue). Scale bar: 20 µm. **J**, HAECs were incubated with exosomes isolated from subcutaneous adipose tissues or serum from obese patients with CAD or nondiseased controls for 24 h. n=9, exosomes from each patient (3 patients per group) were divided into 3 equal parts for coculture experiments. qPCR analysis of miR-30e-5p levels in exosomes and cocultured HAECs. **K** through **M**, Levels of miR-30e-5p in WAT, brown adipose tissue (BAT), serum exosomes, and aortic intima from *ob/ob* mice fed a chow diet or HFD for 8 weeks (**K**); *Apoe*^−/−^ mice fed a chow diet or HFD for 12 weeks (**L**); and C57BL/6 mice injected with adeno-associated virus (AAV)8-control (Ctrl) virus via the tail vein and fed a chow diet (AAV8-Ctrl+CD) or administered AAV8-*PCSK9*^*D377Y*^ virus and fed an HFD (AAV8-*PCSK9*+HFD) for 12 weeks (**M**). Data are mean±SEM from 6 mice per group. Data were analyzed by Mann-Whitney *U* test between the 2 indicated groups (**C**, **J**, **K**, **L**, and **M**). Data were analyzed by Kruskal-Wallis with Dunnett post hoc test among multiple groups in (**E**). Spearman analysis revealed correlations between miR-30e-5p and BMI and TG, LDL-C, and HDL-C levels in (**F**). *P* values are indicated in the figure. The exact *P* values, statistical analyses, post hoc corrections, and sample sizes (n) are listed in Table S9.

### MiR-30e-5p Targets SLC7A11

Having established that adipose tissue-derived miR-30e-5p might be an endocrine-like miRNA regulating adipose-vessel crosstalk, we then investigated the downstream target of miR-30e-5p. For an unbiased screening, we performed RNA sequencing analysis of HAECs transfected with miR-30e-5p or control miRNA. Gene Ontology analysis of differentially regulated pathways showed that the miR-30e-5p level was correlated with nitric oxide biosynthetic process, glutathione metabolic process, regulation of fat cell differentiation, cellular oxidant detoxification, lipids and atherosclerosis, and SLC-mediated transmembrane transport (Figure 2A). The heat map shown in Figure 2B reveals that miR-30e-5p overexpression downregulated genes involved in oxidant detoxification, glutathione metabolic process, and nitric oxide biosynthesis. In addition, miR-30e-5p overexpression affected genes involved in lipid and atherosclerosis as well as SLC-mediated transmembrane transport. Markedly, miR-30e-5p downregulated atheroprotective genes (eg, *SOD2*) and upregulated atheroprone genes (eg, *NOX4*) involved in lipid and atherosclerosis. For SLC family transporters, miR-30e-5p downregulated *SLC7A11* and upregulated *SLC2A3* (GLUT3), which have been shown to affect cellular redox state.^30^ Using TargetScan, we predicted that miR-30e-5p could target the 3′-UTR of *SLC7A11* mRNA (Figure S2A). SLC7A11 level was reduced in dysfunctional ECs due to lipid accumulation (GSE246083) and in ECs exposed to atheroprone flow (GSE118717).^31,32^ These in silico analyses showing miR-30e-5p targeting *SLC7A11* were depicted by Venn analysis in Figure 2C. According to the functional roles of SLC7A11 in cell death and the glutathione redox system (Figure 2D), we theorized that miR-30e-5p targets the 3′-UTR of *SLC7A11* mRNA (Figure S2A). Of note, miR-30e-5p and its targeted sequences in the 3′-UTR of *SLC7A11* mRNA are highly conserved in various mammalian species, including human, mouse, rat, chimp, rhesus, pig, and cow (Figure S2A). We then constructed luciferase reporters fused with the *SLC7A11*-3′-UTR (wild-type) or mutated *SLC7A11*-3′-UTR (MT) to test whether miR-30e-5p directly targets the *SLC7A11* mRNA (Figure 2E). Overexpression of miR-30e-5p suppressed the *SLC7A11*-3′-UTR (wild-type) luciferase activity but not MT activity (Figure 2E). Furthermore, HAECs transfected with miR-30e-5p had lower protein and mRNA levels of SLC7A11 (Figure 2F and Figure S2B). In contrast, inhibition of miR-30e-5p increased levels of SLC7A11 and its mRNA, in a *yin-yang* manner (Figure 2G and Figure S2C). Furthermore, miR-30e-5p and *SLC7A11* mRNA levels were enriched in Ago1 and Ago2, namely, the miRNA-induced silencing complexes, in HAECs transfected with miR-30e-5p (Figure 2H), so *SLC7A11* mRNA was a bona fide target of miR-30e-5p in ECs. Because SLC7A11 regulates the exchange of glutamate and cystine, we analyzed metabolites in HAECs cocultured with adipocytes that were stimulated with palmitate and transfected with or without miR-30e-5p inhibitor. As anticipated, the cocultured HAECs and the secreted exosomes had an increased level of miR-30e-5p, and miR-30e-5p inhibitor abolished this increase (Figure S2D). The untargeted metabolomics depicted by KEGG showed that glutamate and glutathione metabolism were significantly enriched (Figure 2I). In line with these metabolomic changes, HAECs cocultured with exosomes isolated from palmitate-stimulated adipocytes showed an increased level of glutamate and a decreased level of cystine. These glutamate/cystine alterations were partially restored by treatment with the miR-30e-5p inhibitor, despite not being significantly different (Figure 2J). Importantly, incubation of HAECs with serum from obese patients with CAD also led to increased glutamate and decreased cystine levels (Figure 2K). In line with these results, HAECs treated with serum from obese patients with CAD showed reduced cystine uptake, which was rectified by miR-30e-5p inhibition (Figure 2L). Furthermore, given that miR-30e-5p targets *SLC7A11* mRNA, adenovirus (Ad)-mediated SLC7A11 overexpression rescued the cystine uptake by ECs (Figure 2M).

**Figure 2.**
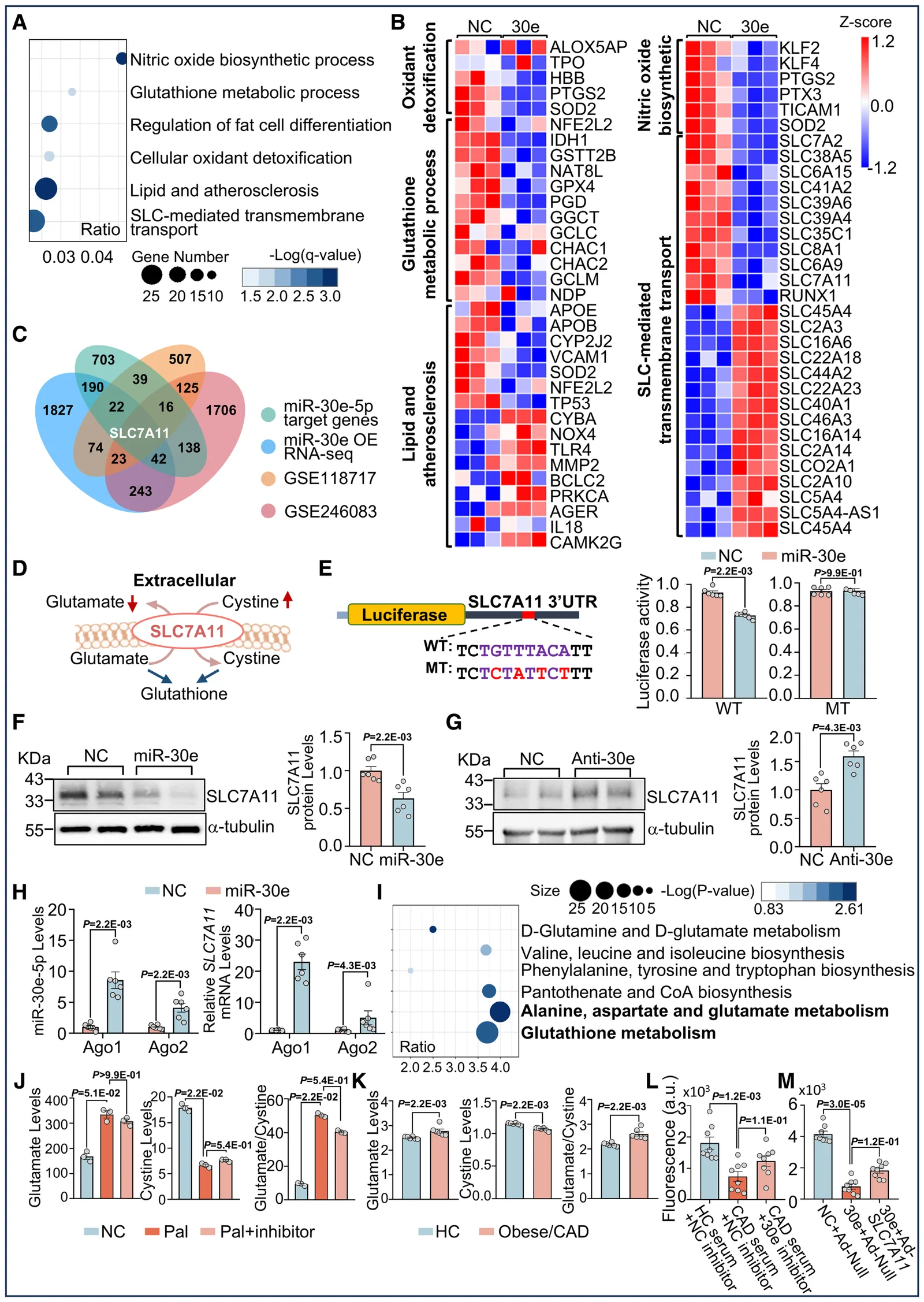
**MicroRNA-30e (miR-30e)-5p targets solute carrier family 7 member 11 (SLC7A11). A** and **B**, Human aortic endothelial cells (HAECs) were transfected with miR-30e-5p or control miRNA (normal control [NC]) for 48 h (n=3). RNAs were isolated and subjected to RNA sequencing (RNA-seq) analysis. Gene Ontology enrichment was delineated by Metascape for the 1690 differentially expressed genes with the cutoff of log2-fold-change >1 or <−1, *p*adj <0.1, and plotted according to gene ratio, gene number and −log (*q* value) of every term (**A**). Heat map comparison of log2-fold-change of the indicated differentially expressed genes according to *Z* scores (**B**). **C**, Venn diagram showing suppressed SLC7A11 as a target of miR-30e-5p (https://www.targetscan.org/vert_80/, light green) in lipid-accumulated endothelial cells (ECs; GSE246083, pink), ECs exposed to atheroprone flow (GSE118717, orange), and miR-30e-5p overexpression (miR-30e OE RNA-seq, blue). **D**, Graphic presentation of the SLC7A11-mediated glutamate/cystine metabolism. **E**, HEK293T cells were cotransfected with miR-30e-5p and a dual luciferase reporter fused with the 3′-UTR of *SLC7A11* (Luc-*SLC7A11*-3′-UTR-wild-type [WT]) or a mutant (MT) reporter with the miR-30e-5p binding site mutated (Luc-*SLC7A11*-3′-UTR MT). The firefly and Renilla luciferase activities were measured (n=6). **F** and **G**, HAECs were transfected with miR-30e-5p or control miRNA (**F**), and miR-30e-5p inhibitor (Anti-30e) or control miRNA (**G**). SLC7A11 was measured by western blot analysis (n=6). **H**, HAECs were transfected with miR-30e-5p or NCs. Ago1 or Ago2 were immunoprecipitated from the transfected ECs. The miRNA-induced silencing complexes (miRISC)-associated miR-30e-5p and *SLC7A11* mRNA were quantified by quantitative real‑time polymerase chain reaction (qPCR) and normalized to levels of Ago1 or Ago2 protein (n=6). **I** and **J**, 3T3L1-derived adipocytes were incubated with or without palmitate (400 μmol/L) for 24 h (Pal; NC). The palmitate-incubated adipocytes were also transfected with miR-30e-5p inhibitor (Pal+inhibitor). n=3 in each of the 3 cell groups. HAECs were incubated with exosomes isolated from the 3 groups of adipocytes, followed by untargeted metabolomic analysis. **I**, Kyoto Encyclopedia of Genes and Genomes (KEGG) enrichment pathways of metabolic variations among NCs, Pal, and Pal+inhibitor groups, *P*<0.05. **J**, Changes in glutamate, cystine, and the glutamate/cystine ratio among these 3 groups. **K**, HAECs were incubated with serum from obese patients with coronary artery disease (CAD) or healthy controls (HC). Levels of glutamate (μmol/L) and cystine (μmol/L) in ECs were measured by ELISA (n=6). **L**, HAECs were transfected with miR-30e-5p inhibitor or control miRNA (NC). Transfected cells were then treated with serum from obese patients with CAD or healthy controls. **M**, HAECs were transfected with miR-30e-5p or NCs, then infected with Ad-*SLC7A11* or Ad-Null (10 multiplicity of infection [MOI]). Cystine uptake by HAECs in (**L** and **M**) was assessed accordingly (n=8, 2 technical replicates from 4 different batches of HAECs). Data were analyzed by Mann-Whitney *U* test between the 2 indicated groups (**E**, **F**, **G**, **H**, and **K**). Data were analyzed by Kruskal-Wallis with Dunnett post hoc test among multiple groups (**J**, **L**, and **M**). *P* values are indicated in the figure. The exact *P* values, statistical tests, post hoc corrections, and sample sizes (n) are listed in Table S9.

### SLC7A11 Is Decreased in Experimental Atherosclerotic Mice and Patients With CAD

To explore the crosstalk between adipocytes and vascular endothelium in the context of miR-30e-5p–SLC7A11 and atherogenesis, we performed single-nucleus RNA sequencing of WAT from *Apoe*^−/−^ mice fed an HFD or chow diet. On t-distributed stochastic neighbor embedding (tSNE) analysis, we identified 5 cell clusters, including adipocytes, epithelial cells, fibroblasts, ECs, and immune cells (Figure 3A), according to their distinct gene expression profiles (Figure S3A). HFD increased the number of proinflammatory macrophages, as signified by increased levels of *Tnf*, *Nlrp3*, and *Cd74* (Figure S3B). In adipocytes and ECs, HFD reduced the *Slc7a11* mRNA level (Figure 3B and 3C). Pseudotime trajectory analysis demonstrated that *Slc7a11* expression was higher in early stages of adipocyte and EC subpopulations (state 1) and decreased afterward to the lowest level in state 5. Moreover, atherosclerotic tissues had the highest level of state 5 cells (Figure 3D and 3E). These results suggest SLC7A11 suppression during the atherogenic course and an inverse association between SLC7A11 abundance and EC homeostasis. In line with this notion, mRNA and protein levels of Slc7a11 were reduced in the aortic intima of atherosclerotic *Apoe*^−/−^ mice (Figure 3F). Slc7a11 level reduction in aortic intima was recapitulated in C57BL/6 mice administered AAV8-*PCSK9*^*D377Y*^ and fed an HFD (Figure 3G). For human atherosclerosis, we performed immunostaining experiments to examine the level of SLC7A11 in atherosclerotic plaque from patients who underwent carotid endarterectomy (demographics in Table S5). Shown in Figure 3H, SLC7A11 expression was reduced in atherosclerotic lesions as compared with nondiseased specimens. To explore changes in metabolites resulting from the differential expression of SLC7A11, we performed untargeted metabolomics analysis of serum from patients with CAD and nondiseased controls (normal controls) in a discovery cohort (demographics in Table S6). Kyoto Encyclopedia of Genes and Genomes (KEGG) enrichment analysis revealed that glutamate metabolism, cysteine and methionine metabolism, and glutathione metabolism varied the most between patients with CAD and normal controls (Figure 3I). With SLC7A11 mediating the extracellular influx of cystine at the expense of intracellular glutamate efflux, we studied the serum metabolites in a validation cohort (demographics in Table S7). The lower level of glutamate associated with a higher level of cystine was confirmed in patients with CAD in the discovery and validation cohorts (Figure 3J and 3L). However, the serum level of glutamate was negatively correlated with BMI (Figure 3K and 3M). Moreover, receiver operating characteristic curves in Figure 3N showed that patients with glutamate levels <37.93% and 31.82% of those in normal controls might have CAD, with sensitivity 41.38% and 40.91% and specificity 96.55% and 90.91% in the discovery and validation cohorts, respectively (area under the receiver operating characteristic curve: 0.68 in the discovery cohort; 0.69 in the validation cohort). Taken together, data in Figure 3 suggest that SLC7A11 was downregulated in the vascular endothelium in patients with CAD as well as mice with experimental atherosclerosis, thus decreasing glutamate level but increasing cystine level in the circulation.

**Figure 3.**
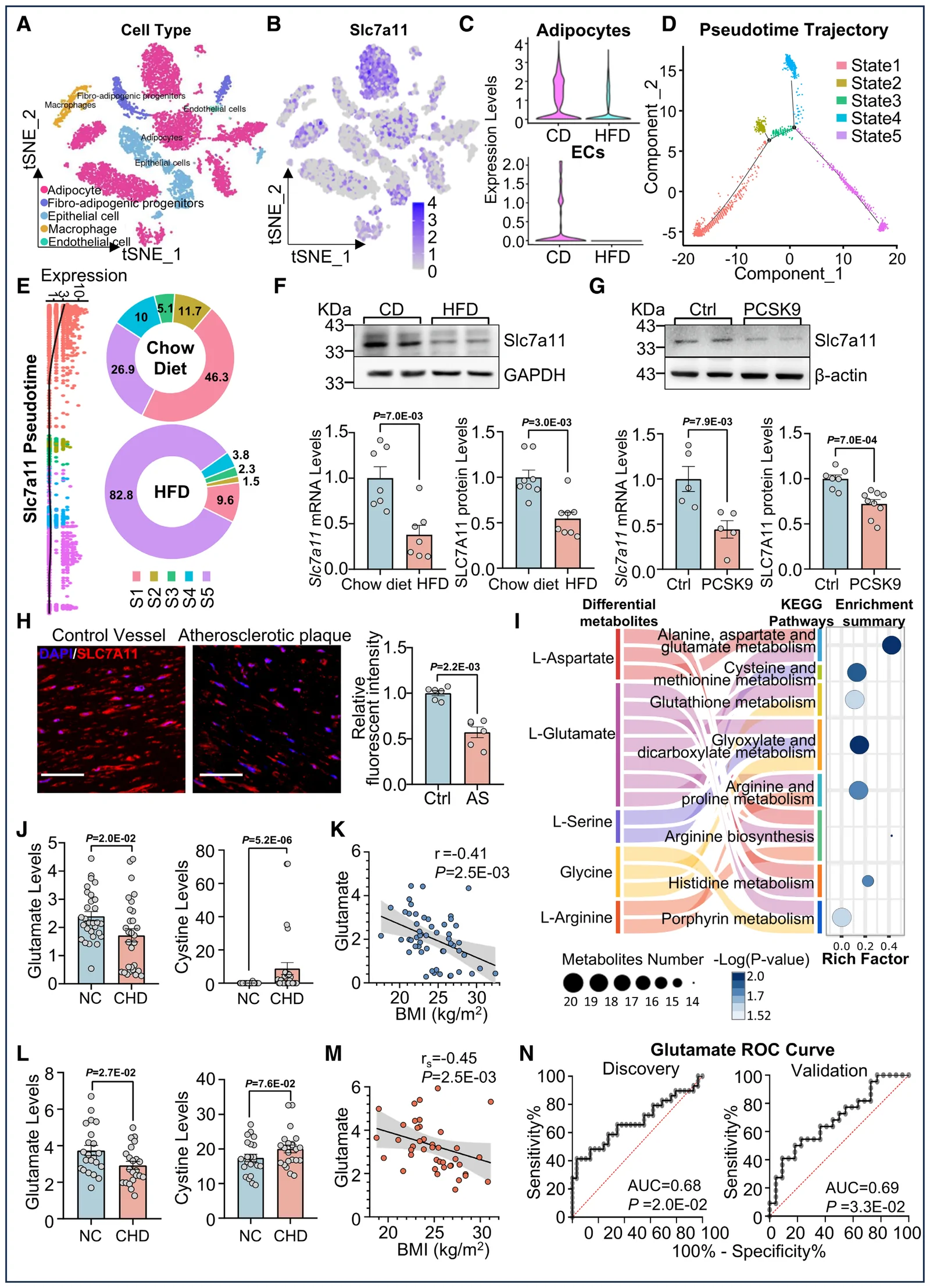
**Solute carrier family 7 member 11 (SLC7A11) is decreased in experimental atherosclerotic mice and patients with coronary artery disease (CAD). A** through **E**, Eight-week-old male *Apoe*^−/−^ mice were fed a high-fat diet (HFD) or chow diet for 12 weeks. Single-nucleus RNA sequencing (snRNA-seq) was conducted on epididymal white adipose tissue (eWAT) collected from 3 animals in each of the 2 groups, which generated transcriptomics from 9540 heterogeneous cells. **A**, t-distributed stochastic neighbor embedding (tSNE) clustering showing 5 annotated cell types in eWAT. **B**, Biaxial scatter plot displaying levels of *Slc7a11* in these 5 cell types, with gray and purple representing low and high levels of *Slc7a11*. **C**, Violin plots showing levels of *Slc7a11* in annotated adipocytes and endothelial cells (ECs). **D**, Cell development pseudotime trajectory plot of nuclei from eWAT, showing 5 evolving stages. **E**, The percentage of cells at each of the 5 stages and the vertical plot on the left reveals the dynamics of *Slc7a11* expression at different stages, with each point as a single nucleus. **F**, Slc7a11 protein and mRNA levels in the aortic intima in *Apoe*^−/−^ mice fed an HFD or chow diet (n=7 for quantitative real‑time polymerase chain reaction [qPCR]; n=8 for Western blot). **G**, Eight-week-old male C57BL/6 mice were administered a single dose of adeno-associated virus (AAV)8-*PCSK9*^D377Y^ (1×10^11^ vg/mouse) by tail vein injection and fed an HFD (PCSK9; n=5 for qPCR; n=9 for Western blot). The second set of mice received AAV8-control (Ctrl) (1×10^11^ vg/mouse) and were fed a chow diet (Ctrl) for 12 weeks (n=5 for qPCR; n=7 for Western blot). Aortic intima protein and mRNA levels of Slc7a11 were assessed. **H**, Representative immunofluorescence staining of SLC7A11 in human carotid atherosclerotic plaques obtained from carotid endarterectomy and control abdominal vessels from autopsy. Specimens were counterstained with DAPI (4′,6‑diamidino‑2‑phenylindole; blue). n=6 (2 technical replicates from 3 biological replicates for each sample, that is, 2 distinct parts for each vessel sample). Scale bar: 50 µm. **I** and **J**, Untargeted metabolomics analysis of serum from patients with CAD and normal controls (NCs) in the discovery cohort (n=29). **I**, Kyoto Encyclopedia of Genes and Genomes (KEGG) pathway-enrichment analysis of differential serum metabolites between CAD patients and nondiseased controls in the discovery cohort (fold change >1.5 or <−1.5, *P*<0.05). The left column lists representative differential metabolites, and the middle column shows the significantly enriched KEGG pathways. Each ribbon links a differential metabolite to the corresponding KEGG pathway in which that metabolite is annotated. The bubble plot on the right summarizes pathway enrichment. Bubble size represents the number of enriched differential metabolites assigned to the pathway, the *x*-axis represents the rich factor, and the color gradient indicates the −log(*P* value), with darker colors representing greater statistical significance. **J**, Variations in glutamate and cystine levels between patients with CAD and NCs. **K**, Correlation between serum glutamate level and body mass index (BMI; n=29). **L** and **M**, Targeted metabolomics analysis of serum glutamate and cystine levels in the validation cohort, confirming the inverse correlation between serum glutamate level and BMI (n=22). **N**, Receiver operating characteristic (ROC) curve with sensitivity and specificity of serum level of glutamate in discovery and validation cohorts, differentiating patients with CAD from NCs. Data were analyzed by Student *t* test (**L**) or Mann-Whitney *U* test (**F**, **G**, **H**, and **J**) between the 2 indicated groups. Spearman analysis in (**K**) and Pearson analysis in (**M**) demonstrate negative correlations between serum glutamate levels and BMI. *P* values are indicated in the figure. The exact *P* values, statistical tests, post hoc corrections, and sample sizes (n) are listed in Table S9.

### MiR-30e-5p–SLC7A11 Regulates Central Carbon Metabolism and Mitochondrial Function

With the established role of SLC7A11 in cystine uptake and biosynthesis, we then explored the effect of SLC7A11 on EC metabolomic changes by using liquid chromatography–tandem mass spectrometry (LC-MS/MS) analysis to compare metabolites in HAECs infected with *SLC7A11* (Ad-*SLC7A11*) versus Ad-Null. SLC7A11 overexpression significantly increased levels of 29 metabolites in ECs versus Ad-Null–infected control cells (Figure 4A; Table S8). Pathway-enrichment analysis revealed that the pathways including central carbon metabolism in cancer, carbon metabolism, ABC transporters, biosynthesis of amino acids, aminoacyl-tRNA biosynthesis, and alanine, aspartate, and glutamate metabolism were significantly changed by SLC7A11 overexpression (Figure 4B). Because glycolysis and the tricarboxylic acid cycle are 2 crucial pathways in central carbon metabolism, we used Seahorse assays to investigate the role of SLC7A11 in regulating EC glycolysis and oxidative phosphorylation. SLC7A11 overexpression in ECs decreased extracellular acidification rate (ECAR) but increased OCR (Figure 4C and 4D). By contrast, SLC7A11 knockdown increased ECAR and decreased OCR (Figure S4A and S4B). Consistent with miR-30e-5p targeting *SLC7A11* mRNA, antisense oligonucleotides against miR-30e-5p increased OCR and decreased ECAR in ECs (Figure S4C and S4D). Furthermore, SLC7A11 overexpression reversed the miR-30e-5p–increased ECAR and miR-30e-5p–decreased OCR (Figure 4E and 4F). These results suggest that the miR-30e-5p–SLC7A11 axis impaired carbon metabolism in ECs. Because of the negative effect of this axis on oxidative phosphorylation, we studied whether it regulates EC mitochondrial function. On confocal imaging using MitoTracker, HAECs transfected with miR-30e-5p showed decreased mitochondria in ECs and increased mitochondrial fragmentation, which was rectified by SLC7A11 overexpression (Figure 4G). These results led us to theorize that this endocrine-like axis causes EC dysfunction. In agreement, SLC7A11 knockdown in ECs increased the expression of *FN1* and *SNAI1* but decreased that of *CDH5* and *NR2F2* (Figure 4H), indicative of augmented endothelial-to-mesenchymal transition. Regarding redox stress, SLC7A11 knockdown increased reactive oxygen species levels in HAECs, and SLC7A11 overexpression reverted the miR-30e–increased reactive oxygen species production (Figure 4I and 4J). Consistently, SLC7A11 deficiency decreased endothelial nitric oxide synthase (eNOS) Ser-1177 phosphorylation, which in turn reduced the level of intracellular nitric oxide, manifesting the reduced eNOS-derived nitric oxide bioavailability (Figure S4H and S4I). In line with this EC functional defect, SLC7A11-deficient HAECs exhibited enhanced migration and proliferation (Figure S4E through S4G), which supports the beneficial effect of SLC7A11 in maintaining EC homeostasis and its EC repair capacity.

**Figure 4.**
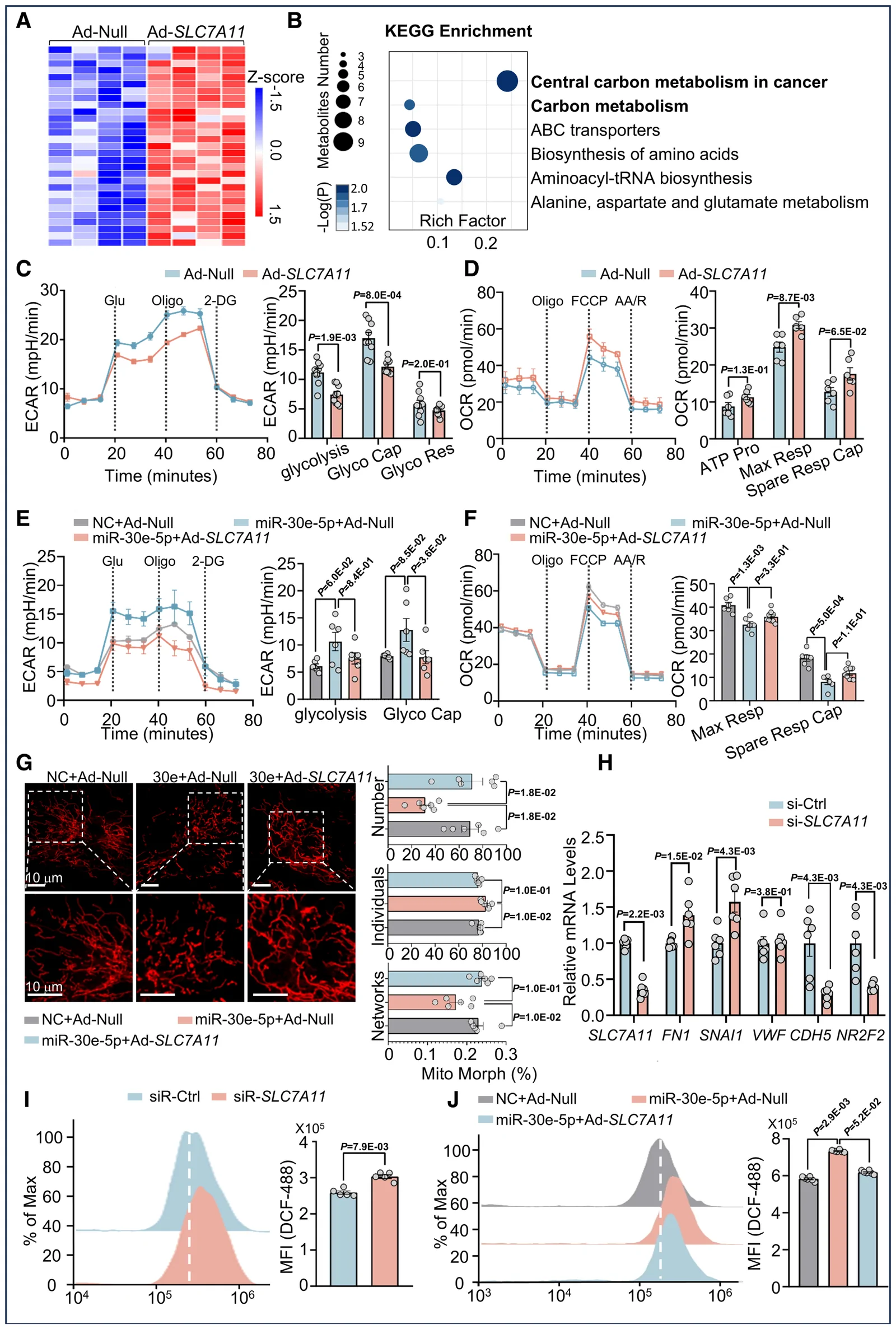
**MicroRNA-30e (miR-30e)-5p–solute carrier family 7 member 11 (SLC7A11) regulates central carbon metabolism and mitochondrial function. A** and **B**, Human aortic endothelial cells (HAECs) were infected with adenovirus (Ad)-*SLC7A11* or Ad-Null for 48 h, then analyzed by untargeted metabolomics (n=4). Fold change ≥1.2 or ≤0.83 and *P*<0.05 were used as the screening criteria. **A**, Heat map showing metabolites differentially regulated by SLC7A11 overexpression. **B**, Kyoto Encyclopedia of Genes and Genomes (KEGG) enrichment analysis of the 29 upregulated metabolites. **C** through **F**, Extracellular acidification rate (ECAR) and oxygen consumption rate (OCR) assays of HAECs infected with Ad-*SLC7A11* (10 multiplicity of infection [MOI]) or Ad-Null (C, n=9 [3 technical replicates from 3 different batches of HAECs]; D, n=6); and transfected with or without normal control (NC) miRNA (NC), miR-30e-5p and with or without Ad-*SLC7A11*, as indicated (**E** and **F**, n=6). **G**, Representative confocal imaging with MitoTracker for mitochondrial morphology in HAECs transfected with NC miRNA or miR-30e-5p and infected with Ad-*SLC7A11* or Ad-Null (n=6, 10 cells counted for each replicate). Scale bar: 10 µm. **H** and **I**, HAECs were transfected with siR-*SLC7A11* or siR-control (Ctrl). Levels of indicated mRNAs were determined in (H, n=6); fluorescence activated cell sorting (FACS) detection of oxidized DCF (2’,7’-Dichlorofluorescein) in (I, n=5). **J**, HAECs were transfected with NC miRNA or miR-30e-5p and infected with Ad-*SLC7A11* or Ad-Null, then analyzed by FACS detection of oxidized DCF (n=6). Data were analyzed by Mann-Whitney *U* test between the 2 indicated groups (**C**, **D**, **H**, and **I**). Data were analyzed by Kruskal-Wallis with Dunnett post hoc test among multiple groups (**E**, **F**, **G**, and **J**). *P* values are indicated in the figure. The exact *P* values, statistical tests, post hoc corrections, and sample sizes (n) are listed in Table S9. 2-DG indicates 2-deoxy-D-glucose; AA/R, antimycin A/rotenone; ATP Pro, ATP production; FCCP, carbonyl cyanide-*p*-(trifluoromethoxy)phenylhydrazone; Glu, glucose; Glyco Cap, glycolytic capacity; Glyco Res, glycolytic reserve; Max Resp, maximal respiration; Oligo, oligomycin; and Spare Resp Cap, spare respiration capacity.

### EC-Specific *Slc7a11* Deficiency Aggravates Atherosclerosis in Mice

To determine the atherogenic effect resulting from the miR-30e–SLC7A11 axis, we created EC-*Slc7a11*^−/−^ mice. Endothelial ablation of *Slc7a11* in this line of mice was validated in isolated pulmonary and aortic ECs (Figure S5A and S5B). To induce atherosclerosis, 8-week-old male and female EC-*Slc7a11*^−/−^ mice (ECKO) and their *Slc7a11*^flox/flox^ wild-type littermates were administered AAV8-*PCSK9*^*D377Y*^ via tail vein injection, then fed an HFD for 12 weeks before being euthanized. Although lipid profiles, including total cholesterol, triglycerides, LDL-C, and high-density lipoprotein cholesterol levels, did not significantly differ among EC-*Slc7a11*^−/−^ and *Slc7a11*^flox/flox^ littermates (Figure S5C), en face Oil-red O staining revealed a significant increase in atherosclerotic lesions in the aortas of EC-*Slc7a11*^−/−^ male and female mice as compared with *Slc7a11*^flox/flox^ littermates (Figure S5D). However, control EC-*Slc7a11*^−/−^ or *Slc7a11*^flox/flox^ mice fed a chow diet for 12 weeks showed little, if any, atherosclerosis (Figure S5D). Increased Oil-red O and Cd68 staining were also evident in the aortic roots of EC-*Slc7a11*^−/−^ mice, thereby reinforcing that *Slc7a11* ablation enhanced the atherosclerosis susceptibility (Figure S5E). Of note, EC-*Slc7a11*^−/−^ mice showed decreased serum level of glutamate and increased level of cystine as compared with *Slc7a11*^flox/flox^ littermates (Figure S5F and S5G), which suggests detrimental effects because of *Slc7a11* deficiency in the vascular endothelium.

### Exogenous miR-30e-5p Exacerbates Atherosclerosis

With the deduced atheroprotective role of SLC7A11 (shown in Figure S5), we then tested whether adipose tissue-derived miR-30e affects aortic levels of SLC7A11 and enhances atherosclerosis. For this, we constructed an AAV9 vector overexpressing a CD63-Flag fusion protein to label the exosomes originated from adipose tissues. This Ad was injected into WAT located in the inguinal area of *Apoe*^−/−^ mice with GW4869 (hinder the secretion of exosomes from adipose tissues) or without. As shown in Figure 5A, these adipocyte-derived exosomes could be uptaken by aortic ECs. However, GW4869 coadministration inhibited this, resulting in a decrease in miR-30e-5p and an increase in SLC7A11 level in the aortic intima (Figure 5B and 5C).

**Figure 5.**
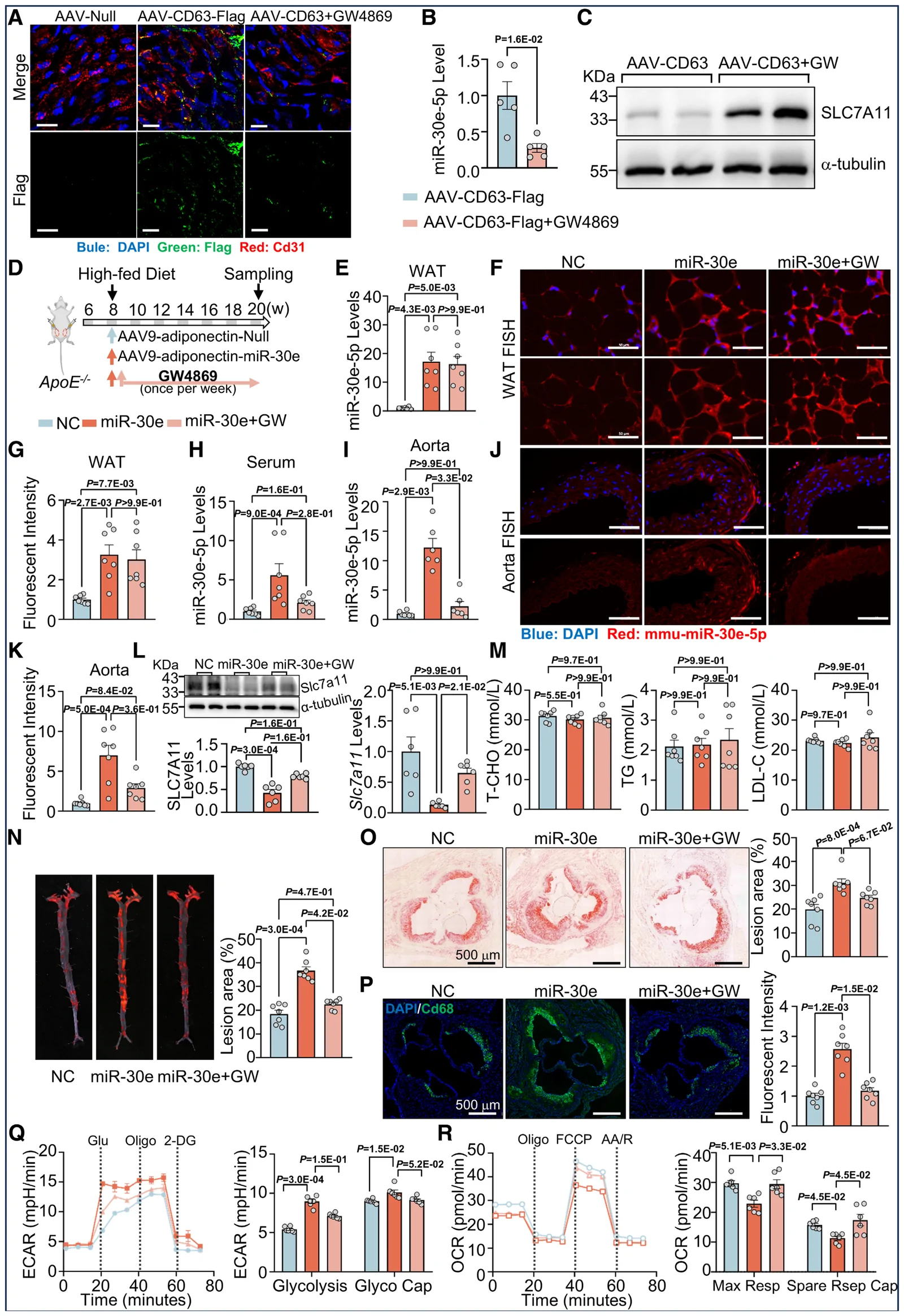
**Exogenous microRNA-30e (miR-30e)-5p exacerbates atherosclerosis. A** through **C**, Male *Apoe*^−/−^ mice at 8 weeks of age were administered 1×10^11^ vg adeno-associated virus (AAV)9-*adiponectin*-CD63-Flag (AAV-CD63-Flag) or AAV9-*adiponectin*-Null (AAV-Null) in white adipose tissue (WAT) in the inguinal area. One day later, mice with WAT overexpressing AAV-CD63-Flag were treated with GW4869 (3 mg/kg) in the inguinal area (AAV-CD63+GW4869). **A**, En face immunostaining of the mouse aorta showing CD63-Flag (green) and CD31 (red) in situ. Scale bar=20 µm. **B**, Levels of miR-30e-5p in the aortic intima were quantified by quantitative real‑time polymerase chain reaction (qPCR) (n=5). **C**, Protein levels of Slc7a11 in aortic intima were assessed by western blot (n=2 per group, each n includes 3 pooled mice). **D**, At 8 weeks of age, male *Apoe*^−/−^ mice fed a high-fat diet (HFD) received 1×10^11^ vg AAV9-*adiponectin*-miR-30e-5p (miR-30e) or AAV9-*adiponectin*-Null (normal control [NC]) in WAT in the inguinal area. One week later, mice with WAT overexpressing miR-30e-5p were administered GW4869 at 3 mg/kg per week in the inguinal area (miR-30e+GW). **E** through **K**, Levels of miR-30e-5p in WAT by qPCR (**E**, n=7) and fluorescence in situ hybridization (FISH; **F** and **G**, n=7), in serum (**H**, n=7), and in aortic intima by qPCR (**I**, n=6) and FISH (**J** and **K**, n=7). Scale bar: 50 µm. **L**, Slc7a11 mRNA and protein levels in mouse aortic intima (n=6). **M**, Levels of total cholesterol (TC), triglycerides (TG), and low-density lipoprotein cholesterol (LDL-C) in the 3 groups of mice (n=7). **N** through **P**, Oil-red O staining of atherosclerosis in aortic specimens and aortic root (**N** and **O**); anti-Cd68 immunostaining (**P**; n=7). **Q** and **R**, Extracellular acidification rate (ECAR) and oxygen consumption rate (OCR) were measured in aortic endothelial cells isolated from 3 groups of mice (n=6 biologically independent cell batches isolated from 6 mice). Data were analyzed by Mann-Whitney *U* test between the 2 indicated groups (**B**), and by Kruskal-Wallis with Dunnett post hoc test among multiple groups (**E**, **G**, **H**, **I**, **K** through **R**). *P* values are indicated in the figure. The exact *P* values, statistical tests, post hoc corrections, and sample sizes (n) are listed in Table S9.

Next, we injected AAV9-*adiponectin*-miR-30e-5p or AAV9-*adiponectin*-Null into WAT located in the inguinal area of *Apoe*^−/−^ mice. The virus recipient mice were then fed an HFD for 12 weeks and treated with or without GW4869 (Figure 5D). The level of miR-30e-5p was significantly elevated in WAT of *Apoe*^−/−^ mice receiving AAV9-*adiponectin*-miR-30e-5p, as compared with mice receiving AAV9-*adiponectin*-Null (Figure 5E through 5G). GW4869 coadministration reduced the circulatory and aortic levels of miR-30e-5p (Figure 5H through 5K), with a complementary increase in *Slc7a11* mRNA and protein in aortas of *Apoe*^−/−^ mice receiving AAV9-*adiponectin*-miR-30e-5p (Figure 5L). All subgroups of mice did not differ in total cholesterol, triglycerides, and LDL-C levels (Figure 5M). At the disease level, AAV9-*adiponectin*-miR-30e-5p administration significantly increased atherosclerosis, compared with mice receiving AAV9-*adiponectin*-Null, and GW4869 coadministration alleviated atherosclerosis (Figure 5N through 5P). Moreover, Seahorse assays showed that WAT-derived miR-30e-5p caused increased ECAR and decreased OCR. As anticipated, GW4869 administration reverted the miR-30e-5p–increased ECAR and miR-30e-5p–decreased OCR in aortic ECs (Figure 5Q and 5R).

To further elucidate the role of miR-30e-5p in WAT-derived exosomes in atherosclerotic disease, we injected WAT-derived exosomes into *Apoe*^−/−^ mice in vivo. As shown in Figure S6, administration of WAT-derived exosomes increased the circulatory and aortic levels of miR-30e-5p, reduced the *Slc7a11* mRNA level in aortas, and aggravated atherosclerosis, as compared with *Apoe*^−/−^ mice receiving PBS. Together, the results in Figure 5 and Figure S6 show that exogenously delivered miR-30e-5p or WAT-derived exosomes, mimicking the obesity-generated miR-30e-5p, aggravate atherosclerosis in *Apoe*^−/−^ mice.

### Inhibition of miR-30e-5p Reduces Atherosclerosis

Complementary to experiments in Figure 5, we used a miR-30e-5p loss-of-function approach to test whether inhibition of miR-30e-5p in adipose tissue could mitigate atherosclerosis by injecting AAV9-*adiponectin*-miR-30e-5p sponge (anti-miR-30e) or AAV9-*adiponectin*-Null (normal control) into WAT of *ob/ob* mice (Figure 6A). Levels of miR-30e-5p were decreased in WAT, blood, and aortic intima. However, levels of the *Slc7a11* mRNA and protein were increased in aortas of *ob/ob* mice receiving AAV9-*adiponectin*-anti-miR-30e, as compared with mice receiving AAV9-*adiponectin*-Null (Figure 6B through 6I). This AAV9-*adiponectin*-miR-30e-5p antagonism significantly alleviated atherosclerosis but had little effect on lipid profile (Figure 6J through 6M). Moreover, miR-30e inhibition in WAT decreased ECAR and increased OCR in aortic ECs isolated from *ob/ob* mice (Figure 6N and 6O). Inhibition of the secretion of adipocyte exosomes by local injection of GW4869 recapitulated the anti-atherosclerotic effect of AAV9-*adiponectin*-miR-30e-5p sponge (Figure 6).

**Figure 6.**
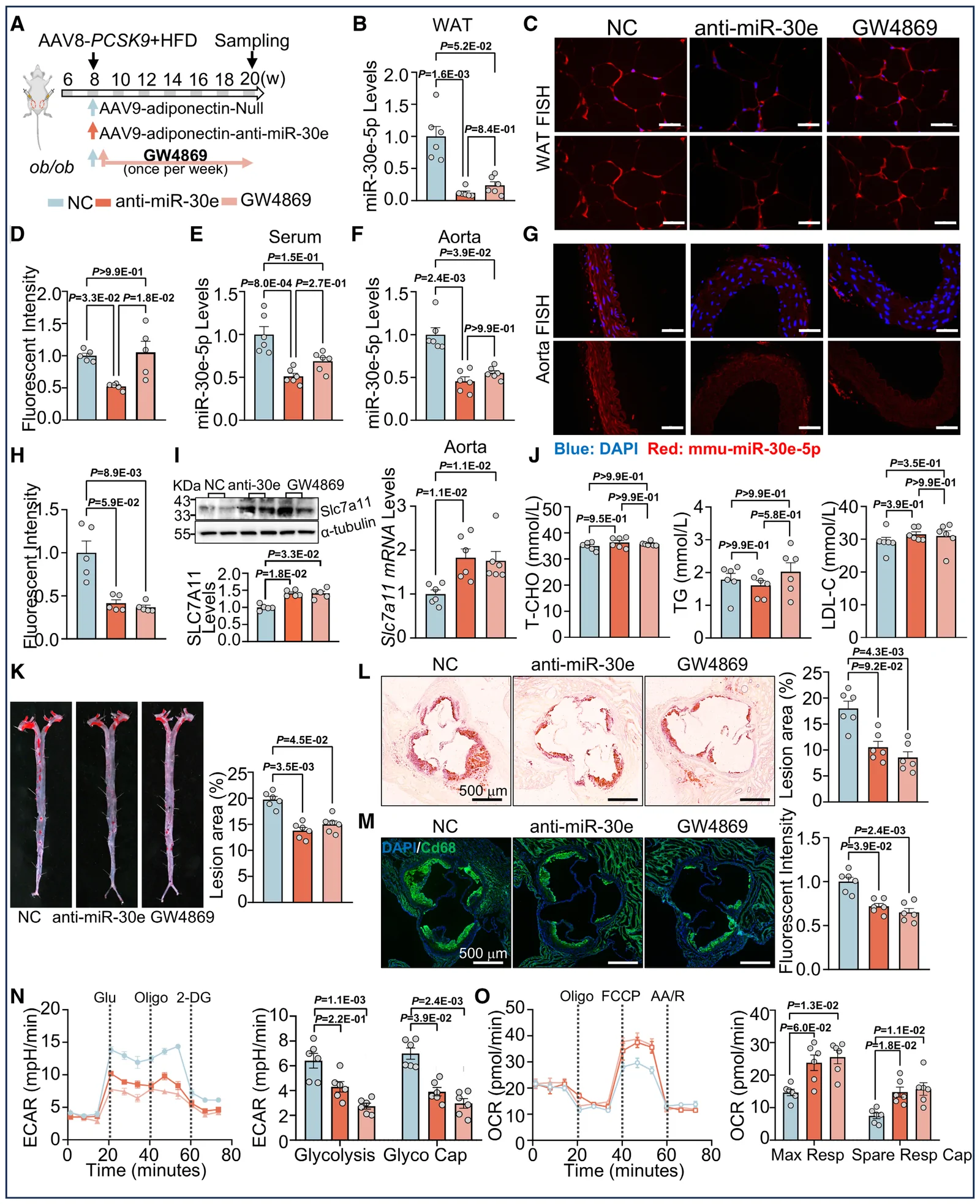
**Inhibition of microRNA-30e (miR-30e)-5p reduces atherosclerosis. A**, Eight-week-old *ob/ob* mice were administered a single dose of adeno-associated virus (AAV)8-*PCSK9*^D377Y^ (1×10^11^ vg/mouse) by tail vein injection and fed a high-fat diet (HFD) to induce atherosclerosis. Mice received 1×10^11^ vg AAV9-*adiponectin*-miR-30e-5p sponge (anti-miR-30e) or AAV9-*adiponectin*-Null (normal control [NC]) in white adipose tissue (WAT) in the inguinal area. One week later, mice with AAV9-*adiponectin*-Null (NC) were administered GW4869 at 3 mg/kg per week in the inguinal area (GW4869). **B** through **H**, Levels of miR-30e-5p in WAT by quantitative real‑time polymerase chain reaction (qPCR) (**B**, n=6) and fluorescence in situ hybridization (FISH; **C** and **D**, n=5), in serum (**E**, n=6), and in aortic intima by qPCR (**F**, n=6) and FISH (**G** and **H**, n=6). Scale bar: 50 µm. **I**, Solute carrier family 7 member 11 (Slc7a11) mRNA and protein levels in mouse aortic intima (n=5 for qPCR; n=6 for Western blot). **J**, Levels of total cholesterol (TC), triglycerides (TG), and low-density lipoprotein cholesterol (LDL-C) in the 3 groups of mice (n=6). **K** and **L**, Oil-red O staining of atherosclerosis in aortic specimens and aortic root (n=6); (**M**) anti-Cd68 immunostaining of the aortic root of the 3 groups of mice (n=6). **N** and **O**, Extracellular acidification rate (ECAR) and oxygen consumption rate (OCR) were measured in aortic endothelial cells isolated from 3 groups of mice (n=6 biologically independent cell batches isolated from 6 mice). Data were analyzed by Kruskal-Wallis with Dunnett post hoc test among multiple groups (**B**, **D**, **E**, **F**, **H** through **O**). *P* values are indicated in the figure. The exact *P* values, statistical tests, post hoc corrections, and sample sizes (n) are listed in Table S9.

For translational relevance, we injected miR-30e-antagomir encapsulated in lipid nanoparticles (LNP-anti-miR-30e-5p) to WAT of *ob/ob* mice (Figure S7A). This administration lowered the level of miR-30e-5p in the injected adipose tissues and also in circulation and aortas (Figure S7B through S7D). It significantly increased the expression of *Slc7a11* in the aortic intima, with attendant reduction of atherosclerosis (Figure S7E through S7H). Because *ob/ob* mice administered LNP-anti-miR-30e-5p or LNP-Control (Ctrl) did not significantly differ in lipid profile or body weight (Figure S7I and S7J), the mechanism by which LNP-anti-miR-30e-5p reduced atherosclerosis would be independent of hyperlipidemia. Rather, LNP-anti-miR-30e-5p administration decreased the circulatory level of glutamate and increased that of cystine as compared with LNP-Ctrl administration (Figure S7K). Altogether, results from animal models with EC ablation of SLC7A11 (Figure S5), miR-30e-5p gain-of-function (Figure 5; Figure S6), and miR-30e-5p loss-of-function (Figure 6; Figure S7) indicate that the endocrine-like miR-30e-5p–SLC7A11 axis would contribute to obesity-aggravated atherosclerosis in rodents.

## Discussion

This study elucidates a pathophysiological causality between obesity and atherosclerosis via an endocrine-like mechanism involving the adipocyte-derived miR-30e-5p and its target SLC7A11 in the vascular endothelium. The major findings are (1) elevated circulating levels of miR-30e-5p in obese individuals with CAD; (2) decreased SLC7A11 levels in patients with CAD and experimental atherosclerotic mice; (3) miR-30e-5p directly targeted the 3′-UTR of *SLC7A11* mRNA, resulting in dysfunctional ECs, including dysregulated thioreduction, increased glycolysis, reduced oxidative phosphorylation, and impaired mitochondrial function; and (4) ablation of *Slc7a11* in endothelium aggravated, but inhibition of miR-30e-5p in adipose tissue alleviated, atherosclerosis in mouse models. From these findings, we propose that miR-30e-5p targeting SLC7A11 links the maladapted adipose tissue with atherosclerosis in the arterial wall. Thus, the miR-30e-5p–SLC7A11 axis might be integral to the cardiovascular-kidney-metabolic syndrome. This notion supports the emerging view that adipose tissue, traditionally considered an energy storage depot, is an active endocrine organ that affects vascular health via secreted factors.^33^

In diet-induced obesity, expanded adipose tissues become deleterious, imposing inflammation and redox stress on the vasculature, thus augmenting atherosclerosis.^9^ Conceptually, adipose tissue constitutes a depot of circulating exosomal miRNAs, a novel mode of cell-to-cell communication regulating gene expression in distant tissues.^12^ Our experiments involving human patients demonstrated a significant elevation of adipose-derived miR-30e-5p in obese individuals with CAD, which was associated with the progression of atherosclerosis. This association was consistent with results from animal experiments revealing increased levels of miR-30e-5p in WAT, circulating exosomes, and aortic tissue in obese rodents. In line with our findings, a previous study showed an increase in miR-30e-5p levels in circulating extracellular vesicles in *db/db* mice, which coincided with coronary microvascular dysfunction and rarefaction.^20^ With obesity significantly increasing the risk of cardiovascular impairments, miR-30e-5p may be multifaceted in the cardiovascular system. Furthermore, our results show that miR-30e-5p–promoted atherosclerosis was independent of LDL-C levels, so miR-30e-5p may directly act on the vascular endothelium rather than affecting liver lipid metabolism to regulate atherosclerosis.

The biological functions of miRNAs are often due to their regulation of multiple targets within the same functional network. We theorize that the adipose tissue-derived miR-30e-5p elicits a detrimental network to disrupt the redox balance (via SLC7A11) and mitochondrial homeostasis (via p53 and BECN1) in ECs.^34,35^ Other miR-30e targets that might also affect EC functions include adrenoceptor α2A and α2B, PLCγ1 (phospholipase C γ1), and *Negr1* (neuronal growth regulator 1).^17,36,37^ Data from our single-nucleus RNA sequencing (Figure 3A) indicated that the levels of adrenoceptor α2A and α2B were low in all cell clusters and almost undetectable in ECs (Figure S3C). PLCγ1, a direct effector of VEGFR2 (vascular endothelial growth factor receptor 2) and other receptor tyrosine kinases, has an atherogenic effect.^37,38^ However, our single-nucleus RNA sequencing data showed that PLCγ1 levels were elevated in adipocytes but decreased in ECs under an HFD (Figure S3C). Of note, *Negr1* variants are associated with BMI and obesity.^39,40^ Genetic ablation of *Negr1* in rodents promoted adiposity and increased plasma glucose and insulin levels.^41^ A recent human population study found adiposity and type II diabetes linked to lower serum levels of NEGR1 (neuronal growth regulator 1).^42^ Consistently, our data revealed that an HFD reduced the level of *Negr1* in both adipocytes and ECs (Figure S3C). Obesity can increase other atherosclerotic risk factors, including diabetes, hypertension, dyslipidemia, and chronic kidney disease.^43–46^ Providing that NEGR1 has a role in BMI and neuropsychiatric regulation, further studies on miR-30e regulation of NEGR1 in terms of chronic kidney disease syndromes are warranted.

With its enzymatic functions of cystine uptake and glutathione biosynthesis, an optimal level of SLC7A11 protects cells against redox and ferroptotic stresses. A previous study showed that the ferroptosis inhibitor ferrostatin-1 reduced iron accumulation and increased the level of Slc7a11 in the aorta of atherosclerotic *Apoe*^−/−^ mice.^24^ Genetic ablation of *IL-17D* (Interleukin-17D) increased the expression of SLC7A11 and decreased atherosclerosis in *Apoe*^−/−^ mice.^47^ Several other miRNAs, such as miR-144-3p, miR-181, and miR-429, can also target *SLC7A11* mRNA.^47–49^

Our finding that miR-30e-5p targeting *SLC7A11* mRNA exacerbated atherosclerosis is novel and significant in several ways. First, the increase in atherosclerosis in *ob/ob* mice administered miR-30e-5p and EC-*Slc7a11*^−/−^ mice recapitulated the causality between obesity-increased miR-30e-5p, EC dysfunction, and atherosclerosis in humans. Second, downregulation of the miR-30e-5p–SLC7A11 axis caused a metabolic shift from glycolysis to mitochondrial oxidative phosphorylation, which enhanced mitochondrial function in ECs. Third, in addition to miR-30e-5p, proinflammatory cytokine TNF-α and oscillatory flow also decreased the level of SLC7A11 in ECs (Figure S8A). These results suggest that other atheroprone factors synergize with obesity to reduce SLC7A11 levels in the vascular endothelium.

The present study has several limitations. Although we established a link between miR-30e-5p and SLC7A11 in the pathogenesis of obesity-related atherosclerosis, the upstream mechanism by which obesity increases the level of miR-30e-5p remains unknown. Previous studies have shown that p53, acting as a transcription factor, can transactivate miR-30e in tumor cells.^50^ Coincidentally, p53 levels are increased in WAT of obese rodents and humans.^51,52^ If indeed the miR-30e-5p–SLC7A11 axis is regulated by p53, the precise cell subcluster in adipose tissue where this occurs deserves further elucidation. With the efficacy of the LNP-delivered anti-miR-30e-5p to reduce atherosclerosis, the translational potential of this preclinical study requires validation in clinical trials. With other miR-30e targets, the off-target effects associated with the antisense RNA approach also need to be considered. Nonetheless, this multiomics study elucidates an adipose-vessel crosstalk involving adipocyte-derived miR-30e-5p and its targeted SLC7A11 in vascular ECs. This endocrine-like axis, linking adipose tissue, endothelial dysfunction, and atherosclerosis, may explain in part the ramifications of obesity-caused atherosclerosis.

## Article Information

### Acknowledgments

The authors thank Drs Meihe Li, Yuanyuan Wei, Wenbo Yang, Yuanming Xing, Baochang Lai, and Juan Zhou at Xi’an Jiaotong University for their technical assistance and consultation. The authors thank Metabo-Profile Biotechnology (Shanghai) Co., Ltd for their metabolomics analysis.

### Author Contributions

C. Wang, J. She, X. Qian, Z.-Y. Yuan, and J.Y.-J. Shyy: conceived of the study and designed the experiments; C. Wang, J. She, X. Qian, H. Wu, W. Hao, X. Liang, Y. Liu, X. Zhuo, K. Deng, Y. Luo, H. Li, Y. Lei, S. Gao, X. Yuan, T. Bai, and Y. Xiong: conducted the experiments; C. Wang, J. She, F. He, X. Dai, S. He, and H. Liu: conducted in silico analysis; C. Wang, J. She, X. Qian, H. Wu, N. Guo, J. Zhang, Z.-Y. Yuan, and J.Y.-J. Shyy: analyzed the data and provided the discussion; and C. Wang, J. She, X. Qian, Z.-Y. Yuan, and J.Y.-J. Shyy: wrote the manuscript.

### Disclosures

None.

### Supplemental Material

Expanded Materials & Methods

Tables S1–S9

Figures S1–S8

Major Resources Table

Uncropped Western Blots

References 53–60

## Supplementary Material
