# Supplementary material for: Obesity Increases Atherosclerosis Susceptibility via an Intertissue miR-30e-SLC7A11 Axis
Source: Circ Res. 2026 Sep 11;139(9):e328493. doi: 10.1161/CIRCRESAHA.126.328493 (PMC13649275; doi:10.1161/CIRCRESAHA.126.328493)
Supplement: Supplementary file 1 [file res-139-e328493-s001.pdf]

Supplemental materials

Expanded Materials and Methods

Online Tables S1-S9

Online Figures S1-S8

Major Resources Table

Uncropped Western Blots

References[53-60]

Materials and Methods

Cell cultures and reagents

Human aortic endothelial cells (HAECs) were obtained from iCell Bioscience (HUM-iCell-c002) and cultured in EC growth medium (PriMed-icell-002) supplemented with 5% fetal bovine serum (FBS), 1% (v/v) penicillin/streptomycin, and 1% EC growth factors (demographics in Table S1). Murine lung and aortic ECs were cultured in EC medium (ECM; ScienCell 1001) supplemented with 5% FBS, 1% (v/v) penicillin/streptomycin, and 1% EC growth factors. Human embryonic kidney 293T (HEK293T) cells and 3T3-L1 murine pre-adipocytes were obtained from American Type Tissue Culture Collection (ATCC) and maintained in Dulbecco's modified Eagle′s medium (DMEM) supplemented with 10% FBS and 1% (v/v) penicillin/streptomycin. 3T3-L1 cells were propagated and differentiated according to standard procedures. Briefly, cells were incubated for 48 hr in DMEM supplemented with 10 μg/mL insulin, 0.5 mM 1-methyl-3-isobutyl-xanthine (IBMX) and 1 μM dexamethasone (Sigma). On day 3, the medium was changed to post-differentiation medium supplemented only with 10 μg/mL insulin until day 10. Fully differentiated 3T3-L1 mature adipocytes identified by lipid droplets in the cytoplasm and Oil-Red-O staining were treated with palmitate (400 mM) for 24 hr. Primary cultures of cells with no more than 5 passages were used in all experiments. All EC cultures were maintained at 37°C in 95% air and 5% CO_2.

Endothelial cell isolation

Lung tissues from EC-*Slc7a11*$^{-/-}$ mice and their wild-type littermates were minced and proteolytically digested with dispase (4 Units/mL) and DNase (100 Units/mL) for 1 hr in Hams medium without serum. Cell suspensions were filtered

through 70 μm cell strainers and centrifuged at 1200 rpm for 5 min, and then incubated with anti-CD144 pre-conjugated Dynabeads for 40 min. ECs were subsequently purified by magnetic separation and seeded on the dish coated with fibronectin. Lung ECs were grown up to 90% confluence and lysed for western blot analysis. Murine aortae were isolated and washed 3 times with ice-cold PBS, and then cut open, incubated with 0.2% collagenase type I at 37°C for 30 min. ECs were collected using a cell scraper and rinsed with DMEM containing 5% serum to arrest the digestion process. The procedures of cell purification were like those of murine lung endothelial cells. Cells were used immediately after reaching confluency or passaged only once.

Exosomes isolation, labeling, and analysis

Cells, dead cells, and cell debris were removed from the conditioned media by centrifugation at 300*g*× for 5 min, 2000*g*× for 15 min and 10,000*g*× for 30 min, respectively. After centrifugation, the supernatant was ultracentrifuged at 120,000*g*× at 4°C for 70 min to pellet the small vesicles (Beckman Optima L-100XP, SW40Ti). The serum was collected and diluted with PBS in a 1:1 ratio, then ultracentrifuged at 200,000*g*× at 4°C for 2 hr. The pellet was washed with PBS and filtrated through a 0.22-μm filter. White adipose tissue（WAT) samples were processed as described with some modifications to obtain tissue exosomes.[1] Briefly, 3 g WAT was washed several times in PBS to eliminate any contaminants and cut into 1-mm^3 pieces. The pieces were then incubated in 0.1% collagenase I at 37˚C for 2-3 hr until the cell suspension became milky. Dulbecco's modified eagle medium (DMEM) supplemented with 10% exosome-free FBS was added to terminate the digestion. The supernatant was collected after centrifugation at 300*g*× for 5 min. Adipose tissue-derived extracellular vesicles (EVs)

were isolated by differential ultracentrifugation as described above. For electron microscopy analysis, EVs were fixed in 4% paraformaldehyde at 4°C for 1 hr, then placed on a formvar-coated grid and negatively stained with 2% (w/v) uranyl acetate. Sections were observed by transmission electron microscopy (HITACHI, HT7800). For nanoparticle tracking analysis (NTA) of EVs, isolated exosome samples were appropriately diluted with 1×PBS buffer to measure the particle size and concentration. NTA measurement was recorded and analyzed at 10 positions. Particle sizes of exosomes were analyzed by nanoparticle tracking analysis with a ZetaView PMX110 analyzer (Particle Metrix, Meerbusch, Germany) and the corresponding software ZetaView 8.04.02 SP2. For co-culture experiments, exosomes with a concentration of 1×10^9 particles/mL, or a protein concentration of 40 μg/mL were co-cultured with HAECs. For EV uptake, 2×10^5 ECs were incubated with 2 μL PKH67 (Sigma-Aldrich)-labeled EVs in 24-well plates for 24 hr at 37°C in 95% air and 5% CO_2. The samples were counterstained with DAPI, then mounted with fluorescent mounting medium. An Olympus IX81 fluorescence microscope was used to acquire fluorescence images.

Human patients and serum samples

All clinical samples were obtained at the First Affiliated Hospital of Xi’an Jiaotong University with informed consent and institutional ethics committee approval (NO. LLSBPJ-2024-483). For the miR-30e-5p detection and metabolomics exploration, serum samples were collected from patients diagnosed of coronary artery disease according to the 2023 AHA/ACC/ACCP/ASPC/NLA/PCNA Chronic Coronary Artery Disease Management Guideline.[2] The exclusion criteria comprised abnormal liver/kidney function, tumor, pregnancy, and other factors deemed unsuitable for inclusion by the researchers. A group of age- and sex-matched healthy subjects were

recruited from the First Affiliated Hospital of Xi'an Jiaotong University as well. Blood samples were taken from patients or healthy controls after 12-hr overnight fasting (Table S3, S6, S7). Serum was collected by centrifugating whole blood at 1500g× for 10 min and quickly frozen at -80°C for storage. Human subcutaneous WAT was obtained from the anterior chest of patients who underwent pacemaker implantation surgery (Table S4). Atherosclerotic plaques were obtained from patients who underwent a carotid endarterectomy (CEA) procedure. All patients had a diagnosis of cerebral infarction before surgery accompanied by clinical symptoms such as dizziness or cognization dysfunction (Table S5).

MiR mimics and inhibitor transfection

The miR-30e-5p mimics (5'-UGUAAACAUCCUUGACUGGAAG-3'; 5'-UCCAGUCAAGGAUGUUUACAUU-3') and inhibitors (5'-CUUCCAGUCAAGGAUGUUUACA-3') were purchased from Genepharma (Shanghai, China). HAECs at 70% confluence were transfected with 40 nM mimics or inhibitors by using lipofectamine 2000 (Invitrogen) in Opti-MEM medium (Gibco) for 6 hr, then changed to complete growth medium. At 48 hr after transfection, cells were lysed or used for other experiments. Equal amounts of GMR-miRTM miRNA controls were used as controls for mimic or inhibitor experiments.

siRNA transfection, adenovirus construction and infection

The double transfection method was used in siRNA transfection experiments. *SLC7A11* siRNA (siR-*SLC7A11*) or scramble control RNA (siR-Ctrl) (Santa Cruz Biotechnology, sc-76933) was transfected into HAECs at 40 nM with lipofectamine 2000 (Invitrogen) followed by a second transfection on the next day. For *SLC7A11* adenovirus (Ad-*SLC7A11*) construction, human *SLC7A11* cDNA was amplified and inserted into the pAdEasy-EF1-MCS-3flag-CMV-shuttle vector, then the virus was

created from the *SLC7A11*-pCMV-shuttle construct by using AdEasy-1 Expression Systems (Hanbio Co. Ltd., Shanghai, China). HAECs were infected with recombinant adenovirus Ad-*SLC7A11* or Ad-Null at 10 multiplicities of infection (MOI) for 48 hr.

Luciferase reporter plasmids, transfection, and luciferase assay

The 3′ untranslated region (3′-UTR) of human the *SLC7A11* gene containing the putative miR-30e-5p targeting sites was synthesized by Shanghai Personal Biotechnology and subcloned into the pmirGLO dual-luciferase miRNA target expression vector (Promega) to obtain Luc-*SLC7A11*-3′-UTR-WT. Mutant constructs were further created with a "TCTATTCT" to "TGTTTACA" replacement in Luc-*SLC7A11*-3′-UTR-mut. The various reporter constructs were transfected into HEK293T cells by using lipofectamine 2000 (Invitrogen). At 24 hr after transfection, cells were lysed for luciferase activity measurement with the Dual-Glo Luciferase Reporter Assay Kit (Promega).

Ago1 and Ago2 immunoprecipitation

HAECs (1.5×10^7 cells/15cm dish) were lysed in a buffer containing 50 mmol/L Tris–HCl pH 7.5, 150 mmol/L NaCl, 0.1% NP-40, 1 mmol/L EDTA, and 100 U/μL RNase inhibitor. An amount of 1 mL lysates was incubated overnight with 100 μL protein G Dynabeads (Invitrogen) conjugated with 5 μL anti-Ago1 (Cell Signaling 5053) or anti-Ago2 (Cell Signaling 2897) at 4°C. The immunoprecipitated RNAs and input RNAs were then extracted by using Trizol (Invitrogen) for qPCR detection. The list of primers used for Ago1 and Ago2 immunoprecipitation is in Supplemental Table S2.

Western blot analysis and q-PCR

For western blot analysis, cells or tissues were lysed in the RIPA buffer supplemented with protease inhibitors (Thermo Scientific). Twenty-five micrograms of

lysates were separated by SDS-PAGE and subsequently transferred onto PVDF membranes. Membranes were blocked with 5% BSA in Tris-buffered saline with 0.1% Tween 20 (TBST) for 2 hr at room temperature, and immunoblotted with primary antibodies at 4°C overnight as indicated. The antibodies used were anti-SLC7A11 (1:800; Cell Signaling, 12691), anti-eNOS (1:1000, BD 610296), anti-p-eNOS S1177 (1:500, Cell Signaling, 9570), anti-β-actin (1:1000; Santa Cruz, sc-47778), anti-GAPDH (1:10000; Abways, AB0037), and anti-α-tubulin (1:20000; Affinity Biosciences, AF7010). The next day, membranes were washed three times for 10 minutes each with TBST, and then incubated with the appropriate horseradish peroxidase-conjugated secondary antibody (anti-rabbit, Jackson, 111-035-045; anti-mouse, Jackson, 515-035-003) for one hour at room temperature. After another round of TBST washing, blots were visualized using enhanced chemiluminescent (ECL) reagents and images were captured using the ChampChemi i610 (SinSage Technology Co., Ltd, China). Protein levels were normalized to those of β-actin, α-tubulin or GAPDH. All raw data from each group were normalized to the mean value of the control group, which was set to be 1.

Total RNA was extracted by using TRIzol reagent (Invitrogen). The mRNA level was measured by using SYBR Green (Bio-Rad) with 18S or β-actin as internal controls. Primer sequences for qPCR are in Supplemental Table S2. For miRNA quantification, the TaqMan MicroRNA Assay followed the manufacturer's protocol (Life Technologies). U6 was detected as the internal control in cultured cells or tissues from animals. To quantify the miR-30e-5p level in serum, a *C. elegans* miR (i.e., Cel-miR-39) was added at 2 nM before RNA extraction as a spike-in control. Quantitative real-time PCR (RT-qPCR) was performed on a Bio-Red CFX Connect real-time PCR detection system. The $2^{-\Delta\Delta Ct}$ method was applied to calculate the target gene expression.

All raw data from each group were normalized to the mean value of the control group, which was set to be 1.

Assessment of extracellular acidification rate and oxygen consumption rate

Extracellular acidification rate (ECAR) and oxygen consumption rate (OCR) were assessed with a Seahorse XF96 flux analyzer (Agilent Technologies). HAECs and murine aortic endothelial cells were seeded at 2×10^4 cells per well in 96-well Seahorse assay plates. Following the manufacturer's instructions, oligomycin (1.5 μM), FCCP (1 μM), and rotenone/antimycin A (R/A) (0.5 μM) were injected sequentially to measure OCR. For the glycolysis stress assay, cells were exposed to glucose (10 mM), oligomycin (1 μM) and 2-deoxy-D-glucose (2-DG; 50 mM), then the ECAR was recorded. After the Seahorse analysis, cells were washed with PBS and stained with DAPI for cell counting. The High-Content Analysis System (Operetta CLS) was used to count cell numbers in each well.

Determination of intracellular reactive oxygen species (ROS)

To measure ROS production, approximately 9×10^5 cells per well in a 6-well plate were washed with PBS once, then stained with 10 μM DCFH-DA (2,7-dichlorodi -hydrofluorescein diacetate) for 30 min at 37°C. After washing the cells 3 times with serum-free medium to fully remove the remaining DCFH-DA, cells were collected and the expression of DCF was detected by fluorescence activated cell sorter (FACS). We established a suitable threshold for fluorescence intensity and compared the ratio of cells exhibiting a positive signal at the designated threshold to the overall cell count.

Cystine uptake assay

Cystine uptake was measured by using the cystine uptake assay kit (UP05, Dojindo Laboratories) according to the manufacturer's instructions. Fluorescence was

measured using the microplate reader. Briefly, HAECs were seeded at 2×10^4 cells per well in 96-well microplates, cells at 70% confluence were transfected with 60 nM miR-30e-5p inhibitors or control miRNA (NC) using Lipofectamine 2000 (Invitrogen) in Opti-MEM medium for 6 hr and then changed to complete growth medium containing 20% CAD sera for 48 hr. Cells were washed with the cystine- and serum-free medium three times and incubated in a cystine- and serum-free medium at 37°C with 5% CO_2 in a humid atmosphere for 5 min. After discarding the medium, cells were incubated with a medium containing cystine analog under the same conditions for 30 min. After washing the cells with cold PBS three times, cells were lysed with cold methanol and incubated with fluorescent probe at 37°C for 30 min. Then, fluorescence intensity was measured by using a microplate reader (Ex/Em=490/535 nm). The blank for the assay was measured by the same procedure but without using cystine analog.

Mito-Tracker staining

MitoTracker Red CMXRos (Beyotime, Shanghai) was used to specifically label the bioactive mitochondria. The storage solution of MitoTracker Red CMXRos was prepared at 200 μM. Before staining, the storage solution was diluted at a ratio of 1:1000 with prewarmed HBSS at 37°C to prepare the working solution. HAECs were seed onto μ-8 well-slides (ibidi, Germany) at 2×10^4 cells/well and incubated with the MitoTracker Red CMXRos working solution at 37°C for 30 min. The MitoTracker Red CMXRos working solution was removed and fresh prewarmed cell culture medium was added. Images were acquired by using a Leica STELLARIS 5 confocal laser scanning microscope. The number of mitochondria, individual mitochondria, and mitochondrial networks were analyzed by ImageJ.

Measurement of nitric oxide

Intracellular nitric oxide (NO) levels were detected by fluorescent 3-Amino,4 aminomethyl 2',7'-difluorescein, diacetate (DAF-FM-DA) dye (Beyotime, S0020S). HAECs were seeded on the μ-8 well-slides (ibidi, Germany) at 4×10^4 cells per well. Following overnight incubation at 37°C, the culture medium was replaced with 5 μM DAF-FM DA, followed by incubation at 37°C for 20 min. Then, the cells were washed with PBS three times. After washing, samples were mounted with mounting medium supplemented with DAPI (Beyotime Biotechnology P0131) and observed under a confocal microscope (Leica) at 495 nm excitation and 515 nm emission filters. For assessment of fluorescence intensity of DAF-FM DA, ECs were seeded in a black 96-well plate with 5×10^4 cells per well and processed identically. Fluorescence intensity was measured by a fluorescence spectrophotometer (ex. 495 nm and em. 515 nm).

EC functional assays

For EdU incorporation assay, 4×10^4 HAECs/well were cultured and treated on μ-8 well-slides (ibidi, Germany). According to the manufacturer's instructions of the BeyoClick™ EdU-555 Cell Proliferation Assay Kit (Beyotime Institute of Biotechnology, China), the EdU solution was added to the cell culture medium at a final concentration of 10 μM and incubated for 2.5 hr. After fixation and permeabilization, the Click reaction was performed to detect EdU incorporation, and the nuclei were counterstained with DAPI. Images were captured under a fluorescence microscope, and the percentage of EdU-positive cells was calculated from randomly selected fields. Cell migration was assessed with 8-μm transwell inserts (Corning). HAECs (5×10^4 cells per insert) were seeded into the upper chamber with complete medium in the lower chamber. After 24 hr, migrated cells were fixed with 4% PFA, stained with 0.1% crystal violet, and counted from six random fields per membrane. For wound healing assay, HAECs were seeded into 6-well plates at a density of 1×10^6 cells per well and grown to a

confluent monolayer. A straight scratch wound was generated by manually dragging a sterile P200 pipette tip perpendicularly across the bottom of each well. Detached cells were removed by washing gently twice with PBS. The wound area was marked for consistent field acquisition. Phase-contrast images were taken at 0 and 8 hr post-wounding using an inverted microscope equipped with a digital camera. Three non-overlapping fields per well were captured. Wound closure was analyzed using ImageJ.

Untargeted metabolomics

Quantification of serum metabolites was performed by Metabo-Profile (Shanghai) using previously published methods with minor modifications.[3-5] Briefly, the untargeted metabolomics was performed on an XploreMET platform-based Agilent 7890B gas chromatograph. The sample preparation, instrumentation, metabolic annotation and data analysis procedure involved published methods with minor modification.[6-8] Cell samples were prepared and analyzed in LC/MS platforms by BGI Genomics. In short, 1×10^7 cells were crushed in 140 μL of 50% water/methanol solution, then centrifuged at 3000*g*× for 20 min at 4°C. The supernatant was collected and diluted in 140 μL HM400 diluent (HM400 metabolites kit) and centrifuged at 18,000*g*× for 10 min at 4°C, and metabolites was taken for LC-MS/MS analysis. A quality control (QC) sample was prepared by pooling the same volume of each sample to evaluate the reproducibility of the whole LC-MS analysis. The sample extracts were analyzed by using Waters UPLC I-Class Plus (Waters, USA) equipped with QTRAP 6500 Plus (SCIEX, USA). Chromatographic conditions: Samples injected in the BEH C18 (2.1 mm x 10 cm, 1.7 μm, waters) were eluted for multiple reaction monitoring (MRM) with the following LC gradient: phaseUltra pure water (mobile phase A) and acetonitrile (mobile phase B). The gradient conditions were as follows: 0-1 min, 5%

mobile phase B; 1-5 min, 5-30% mobile phase B; 5-9 min, 30-50% mobile phase B; 9-11 min, 50-78% mobile phase B; 11-13.5 min, 78-95% mobile phase B; 13.5-16 min, 95-100% mobile phase B; 16.1-18 min, 5% mobile phase B. With flow rate at 0.4 mL/min, the column temperature was set at 40°C. Mass spectrum conditions: For the QTRAP 6500 Plus equipped with an EST Turbo Ion-Spray interface, the source parameters were set as follows: source temperature: 500°C; ion spray voltage (IS): 4500 V (positive mode) or -4500 V (negative mode); ion source gas I (GS1), gas II (GS 2) and curtain gas (CUR) set at 40, 40 and 20 psi, respectively. MRM methods were set at schedule mode with MRM transitions, collision energy (CE), declustering potential energy (DP) and retention time for target metabolites. Data analysis, including principal component analysis and hierarchical clustering was performed using R. Metabolic pathway enrichment analysis of differential metabolites based on the KEGG database revealed significantly altered metabolic pathways.

Library preparation, RNA-sequencing, and data analysis

HAECs were transfected with miR-30e-5p mimic or control mimic (40 nM) for 48 hr, and total RNA was extracted and purified with TRIzol reagent (Invitrogen) following the manufacturer's procedure. The RNA amount and purity of each sample was quantified by using NanoDrop ND-1000. The RNA integrity was assessed by using Bioanalyzer 2100 (Agilent) with RIN number >7.0, and confirmed by electrophoresis with denaturing agarose gel. Poly(A) RNA was purified from 1 μg total RNA with Dynabeads Oligo (dT)25-61005 (Thermo Fisher) and two rounds of purification. Then poly(A) RNA was fragmented into small pieces by using Magnesium RNA Fragmentation Module (NEB) under 94°C for 5-7 min. Then the cleaved RNA fragments were reverse-transcribed to create the cDNA by using SuperScript II Reverse Transcriptase (Invitrogen), which was then used to synthesize U-labeled second-

stranded DNAs with *E. coli* DNA polymerase I (NEB), RNase H (NEB) and dUTP Solution (Thermo Fisher). An A-base was then added to the blunt ends of each strand, preparing them for ligation to the indexed adapters. Each adapter contained a T-base overhang for ligating the adapter to the A-tailed fragmented DNA. Single- or dual-index adapters were ligated to the fragments, and size was selected using AMPureXP beads. After the heat-labile UDG enzyme (NEB) treatment of the U-labeled second-stranded DNAs, the ligated products were amplified with PCR with the following conditions: initial denaturation at 95°C for 3 min; 8 cycles of denaturation at 98°C for 15 sec, annealing at 60°C for 15 sec, and extension at 72°C for 30 sec; then final extension at 72°C for 5 min. The average insert size for the final cDNA library was 300±50 bp. Finally, we performed the 2×150-bp paired-end sequencing (PE150) on an illumina Novaseq 6000 (LC-Bio Technology, Hangzhou, China) following the vendor's recommended protocol. Fastp software (https://github.com/OpenGene/fastp) was used to remove the reads that contained adaptor contamination, low-quality bases and undetermined bases with default parameter. Then sequence quality was verified by using fastp software. We used HISAT2 (https://ccb.jhu.edu/software/hisat2) to map reads to the reference genome of *Homo sapiens* GRCh38. The mapped reads of each sample were assembled by using StringTie (https://ccb.jhu.edu/software/stringtie) with default parameters. Then, all transcriptomes from all samples were merged to reconstruct a comprehensive transcriptome by using gffcompare (https://github.com/gpertea/gffcompare/). After the final transcriptome was generated, StringTie was used to estimate the expression levels of all transcripts. by calculating FPKM (FPKM = [total_exon_fragments / mapped_reads(millions)×exon_length(kB)]). DESeq2 R package was used to analyze the differential expression of two groups. The gene counts obtained from RNA-Seq were screened by using the R package DESseq2

to screen differentially expressed genes (DEGs) between NC mimics and miR-30e-5p (log2FoldChange >1 or <-1, padj < 0.1). Enrichment analysis of DEGs was conducted using the online tool Meatscape (https://metascape.org). The program was run with default settings, with significant Gene Ontology (GO) terms and pathways identified ranking by -Log(p-value) (obtained by hypergeometric test). A Venn diagram was created using the OmicStudio tools at https://www.omicstudio.cn/tool.

Single-nucleus RNA-sequencing (snRNA-seq) analysis

Nuclei were isolated by using Nuclei EZ lysis buffer (NUC-101; Sigma-Aldrich) supplemented with protease inhibitor (Roche) and RNase inhibitor (Promega). Frozen mouse WAT was thawed on ice, minced to 1 mm^3 and homogenized by using a Dounce homogenizer in the ice-cold Nuclei EZ lysis buffer for 5-10 min, and 4% BSA was added to stop the reaction. The minced adipose tissue was filtered through a 40-μm cell strainer and centrifuged at 500*g*× for 5 min at 4 °C. The pellet was washed twice with lysis buffer, then resuspended in nuclei suspension buffer (1× PBS,0.07% BSA, and 0.1% RNase inhibitor), and counted by using a haemocytometer/Countess II Automated Cell Counter; the concentration adjusted to 700-1200 cells/μL. Single-nuclei suspensions were loaded to 10× chromium to capture 8000 single cells according to the manufacturer's instructions of the 1× Genomics Chromium Single-Cell 3' kit (V3). The following cDNA amplification and library construction steps were performed according to the standard protocol. Quality control and data processing were performed by LC-Bio Technology (Hangzhou, China). 10× genomics raw data were processed by using the Cell Ranger Single-Cell Software Suite (release 2.0), including using cellranger mkfastq for demultiplexing raw base call files into FASTQ files and then using cellranger count for alignment, filtering, barcode counting, and unique molecular identifier (UMI) counting. The reads were aligned to the GRch38.96 reference genome

by using a prebuilt annotation package downloaded from the 10× Genomics website. The outputs from different lanes were finally aggregated by using cellranger aggr with the default parameter settings. Cells were flagged as poor-quality if 1) the number of expressed genes was < 500 and 2) 25% or more UMIs mapped to mitochondrial genes. Cells meeting the latter threshold were usually nonviable or apoptotic. Genes with high variation were identified by using FindVariableFeatures. Principal component analysis (PCA) for dimensional reduction was performed with the use of Seurat function RunPCA. FindNeighbors and FindClusters functions were used in the Seurat package for cell clustering analysis and the 2D map displayed by using tSNE. The FindAllMarkers function in Seurat was used to identify unique cluster-specific marker genes. Clusters were identified with the dominant expression pattern of cell markers from the online database and published literature. Putative clusters with common canonical markers were integrated into one cluster. Violin plots, heatmaps, and cluster annotations were created in Seurat with the corresponding footnotes. R package clusterProfiler was used for enrichment analyses. Marker genes detected in >10% cells in a cluster with average log-fold-change > 0.26 were used in the analysis.

Adeno-associated virus (AAV) recombination and infection, and GW4869 administration in WAT

AAV9-*adiponectin*-CD63-3xFlag, AAV9-*adiponectin*-miR-30e-5p and AAV9-*adiponectin*-miR-30e-5p sponge were constructed, amplified, and purified by HANBIO Biotechnology Co. Ltd (Shanghai, China). Briefly, mus CD63, mus-miR-30e-5p sequence (GTACAAATAACCAGTCAGAAAACCTGTTGGAGAAGTGGGCATCCTAATT GACATTAATTCTGACCCTGCATGAGGGTTTTTGGGTTAGCAAGCCCTGGGT CACCTCCTTATGGGAGCAGGTTGTGCCTCACTGAAGCTCTTTATTTTTCTG

GGCAGTCTTTGCTACTGTAAACATCCTTGACTGGAAGCTGTAAGGTGTTGAGAGGAGCTTTCAGTCGGATGTTTACAGCGGCAGGCTGCCACAGTCGTCCCCGGCTGCGCTCATCGGCTTTGAGACATTGCAGGTTTGTGCCCATGAACTTGTCTCCTTTTCTTGGGTTACTTCCTTTCTCTCTGAGAAGTCACAAGAACTCAGACTGTCTCTCTTCGTTTGGAGCTGATAATACCATGAG) and mus-miR-30e-5p sponge (CTTCCAGTCATCCTGTTTACAtatacCTTCCAGTCATCCTGTTTACAacatcCTTCCAGTCATCCTGTTTACAtcttcaCTTCCAGTCATCCTGTTTACA) were cloned into the pHBAAV-adiponectin-T2A-ZsGreen vector respectively, followed by sequencing confirmation, then the shuttle vector was packaged into capsids from AAV serotype 9. Viral particles were purified and then titered by qRT-PCR analysis. 1×10^11 vg of AAV9-*adiponectin*-CD63-3xFlag, AAV9-*adiponectin*-miR-30e-5p or AAV9-*adiponectin*-miR-30e-5p sponge was injected into the WAT in the inguinal area of mice at multiple spots using Hamilton syringe. The control mice were injected with the same amount of AAV9-*adiponectin*-NC vector. For GW4869 *in vivo* treatment, 50 μL of GW4869 (3 mg/kg/wk) (MCE, 6823-69-4) was also locally injected into the WAT in the inguinal area of mice at multiple spots using Hamilton syringe.

Animal studies, measurements, and histology

Animal experiments were approved by the Institutional Animal Ethics Committee of Xi'an Jiaotong University (NO. 2022-170). All rodents were kept on a 12-hr light/dark cycle and fed *ad libitum* with chow diet at room temperature. To generate endothelium-specific *Slc7a11* knockout mice (EC-*Slc7a11*$^{-/-}$), the floxed *Slc7a11* allele homozygous mice (*Slc7a11*$^{flox/flox}$) (Cyagen Biosciences Inc, Suzhou, China) were crossed first with the VE-Cad-CreERT2 line on the C57BL/6 background. Six-week-old *Slc7a11*$^{flox/flox+}$Cre^{+} or their littermates (*Slc7a11*$^{flox/flox+}$Cre^{-}) mice were

intraperitoneally injected with tamoxifen (Sigma-Aldrich, 20 mg/mL, 75 mg/kg body weight) once a day for 5 days. EC-*Sla7a11*$^{-/-}$ mice were viable and fertile. *Slc7a11* deletion was verified by detecting Slc7a11 level in lung and aortic ECs by western blot analysis. Male and female mice at 8-9 weeks old fed an high-fat diet (HFD) (Research Diets, D12109C) or chow diet were administered AAV8-Null or AAV8-*PCSK9*^{D377Y} (1×10^{11}vg/mouse) by tail vein injection. After 12 weeks, mice were euthanized and blood was collected from the retro-orbital veins. Serum lipid profile, including total cholesterol (TC), triglycerides (TG), low-density lipoprotein cholesterol (LDL-C), and high-density lipoprotein cholesterol (HDL-C), were measured according to the manufacturer's manuals (Nanjing Jiancheng Biotechnological). Subsequently, the aortic tree was flushed with 10 mL PBS through the left ventricle, then perfused with 4% paraformaldehyde. The entire aortic trees were opened longitudinally, stained with Oil-red O and digitally scanned. The *en face* lesion area was assessed by using WinROOF 6.5 (Mitani Co., Fukui, Japan). For *en face* immunostaining, the aorta was permeabilized in Triton X-100 (0.02%) in PBS for 1 hour, followed by blocking in 5% BSA in PBS for 1 hour at room temperature. Samples were incubated with primary antibodies against CD31 (1:50, Invitrogen, 14-0311-82) and Flag (1:100, Proteintech, 20543-1-AP) for 24 hr at 4°C. The aorta was then incubated with goat anti-rabbit Alexa Fluor 488 (1:200; Servicebio GB25303) and donkey anti-mouse Cy3 (1:200; Servicebio GB21401) for 2 hr at room temperature. Aorta segments were cut to expose the endothelium layer, and mounted with mounting medium supplemented with DAPI (Beyotime Biotechnology P0131). The isotype-matched IgGs (Rabbit mAb IgG, Cell Signaling, 3900S; Mouse mAb IgG, Cell Signaling,61656S) were used to validate antibody specificity. The secondary antibody alone was used as negative control to distinguish non-specific background signals. Images were taken using a confocal

microscope (Stellaris 5; Leica) and LAS X software (Leica). Each heart was cut in a plane between the lower tips of the right and left atria. The upper portion was embedded in OCT compound. Then, the aortic root was sectioned (5 µm) serially from the start of the aortic valve to the start of the ascending aorta until the valve cusps were no longer visible. Frozen sections of hearts were stained with Oil-red O, and observed under a NanoZoomer Slide Scanner. MiR-30e-5p antagomir and agomir were designed and synthesized by Genepharma (Shanghai, China). One week before HFD feeding, male and female *Apoe*$^{-/-}$ mice at 8 weeks old received tail-vein injections of negative control-miR or miR-30e-5p-agomir/antagomiR at 16 mg/kg body weight. The second and third doses of agomir/antagomiR were given after 4 weeks and 8 weeks of HFD feeding, respectively. The mice were euthanized at the end of the 12th week after HFD feeding. Fluorescence *in situ* hybridization (FISH) assay was performed using the FISH Kit (H0101, GenePharma, China) according to the manufacturer's instructions. Briefly, tissue samples were paraffin-embedded and cut into 5-µm sections. The slides were then heated at 65°C for 2 hr, followed by de-paraffinization, hydration and antigen retrieval. Subsequently, slides were covered in 20 µg/mL Proteinase K, incubated at 37 °C for 30 min, and washed 3 times in PBS. After pre-hybridization, Cy3-labelled-mmu-miR-30e-5p RNA probes from Sangon Biotech (5'-CUUCCAGUCAAGGAUGUUUACA-3') were hybridized in the prepared hybridization buffer overnight at 37°C. The hybridized slides were washed with 2× SSC, 1× SSC, 0.5× SSC, then stained with DAPI. Staining results were captured by microscopy. The lipid nanoparticles (LNPs) were prepared and synthesized by APExBIO Technology LCC using NanoAssemblr Ignite (Precision Nanosystems, Canada). Briefly, the lipid components (DLin-MC3-DMA, DSPC, cholesterol, and PEG2000-DMG) were fully dissolved in ethanol at a molar ratio of 50:10:38.5:1.5 to a

total lipid concentration of 25 mM total lipid. The miR-30e-5p-antagomiR was dissolved in 25 mM sodium acetate buffer (pH=4). The two solutions were mixed by using a microfluidic chip at a total flow rate of 12 mL/min and a flow rate ratio of 3:1 (aqueous: organic phase, v/v). The formulations were then dialyzed and concentrated. The supernatants were filtered through 0.22-μm sterile polyethersulfone membranes and stored at 4°C for use within 1 month. Eight-week-old male *ob/ob* mice were purchased from, injected with AAV8-$PCSK9^{D377Y}$ via tail vein and fed an HFD to induce atherosclerosis. One week before AAV8-$PCSK9^{D377Y}$ administration, miR-30e-5p antagomir or control antagomir packed in lipid nanoparticles (LNP-anti-miR-30e-5p/LNP-Ctrl) was injected into WAT in the inguinal area of mice at 2 mg/kg body weight. Subsequently, mice were treated with LNP-anti-miR-30e-5p or LNP-Ctrl once a week and euthanized at the end of week 8 after HFD feeding.

ELISA

Glutamate and cystine levels in endothelium and serum were measured by using a human or mouse glutamate and cystine detection kit according to the manufacturer's protocols (FANKEW, Shanghai). Briefly, 1×10^7 cells were lysed in the 400 μL RIPA buffer supplemented with protease inhibitors (Thermo Scientific). An amount of 50 μL lysates or diluted serum samples were incubated in precoated plates for 30 min at 37°C. After 30 min of incubation with horseradish peroxidase–conjugated secondary antibody, TMB substrate solution was added and samples were incubated further for 10 min before stop buffer was added. Plates were read immediately by using a microplate reader (BioTeK) set at 450 nM.

Statistics

All statistical analyses were performed with GraphPad Prism 9. Initially, the datasets were analyzed for normality with the Shapiro-Wilk test (P>0.05) and equal variance with the Brown-Forsythe test (P>0.05). No normality assumption was made via the Central Limit Theorem. For data with normal distribution, two-tailed Student *t*-test was then used to compare two groups and one-way ANOVA with Bonferroni post *hoc* test for multiple groups. Nonparametric data and groups with n<10 were analyzed using the Mann-Whitney *U* test for 2 groups or the Kruskal-Wallis test for >2 groups. Receiver Operating Characteristic (ROC) analysis was used to evaluate the overall accuracy of serum levels of miR-30e-5p and glutamate in distinguishing between CAD and normal controls. The areas under the curve (AUC) were calculated using the Wilson/Brown method, with higher values indicating greater predictive accuracy. For correlation analyses, potential outliers were identified using the ROUT method (Q=1%) in GraphPad Prism. Pearson or Spearman's rank correlation analyses were then conducted after excluding influential outliers. Data are expressed as mean±SEM; P<0.05 was statistically significant. Statistical details of the experiments can be found in the Table S9. No experiment-wide multiple test correction was applied. Images with the value closest to the average value were selected as representative ones.

Supplemental Table 1. Information on healthy donors used for isolating HAECs.

	Donor 1	Donor 2	Donor 3	Donor 4	Donor 5	Donor 6
Age (years)	32	26	41	28	37	25
Sex	Female	Female	Female	Male	Male	Male

Supplemental Table 2. Primers used for qPCR.

Sites	Forward 5′–3′	Reverse 5′–3′
**Human**		
β-actin	CATGTACGTTGCTATCCAGGC	CTCCTTAATGTCACGCACGAT
SLC7A11	GGTCCATTACCAGCTTTTGTACG	AATGTAGCGTCCAAATGCCAG
18S	CTTTGGTCGCTCGCTCCTC	CTGACCGGGTTGGTTTTGAT
FN1	CGGTGGCTGTCAGTCAAAG	AAACCTCGGCTTCCTCCATAA
SNAI1	GCTGCAGGACTCTAATCCAGA	ATCTCCGGAGGTGGGATG
VWF	AGTGAGCCTCTCCGTGTATC	TCACCGGACAGCTTGTAGTA
CDH5	GTTCACCTTCTGCGAGGATA	GTAGCTGGTGGTGTCCATCT
NR2F2	CGGGTGGTCGCCTTTATGG	ACAGGCATCTGAGGTGAACAG
**Mouse**		
β-actin	GGCTGTATTCCCCTCCATCG	CCAGTTGGTAACAATGCCATGT
Slc7a11	GGCACCGTCATCGGATCAG	CTCCACAGGCAGACCAGAAAA
**Ago-IP**		
SLC7A11	ACTGGGAAGAAGCTCATAGTGG	TGTAAACAGAGGCTATCAAGGTGA

Supplemental Table 3. Clinical characteristics of patients with coronary artery disease (CAD) and healthy controls used in Figure 1.

	Healthy controls (N=85)	CAD patients (N=85)	*p*-value
Age (years)	53.68±13.85	57.21±11.12	9.1×10^(-2)
Sex (male)	55 (64.71%)	61 (71.76%)	3.2×10^(-1)
SBP (mmHg)	120.17±12.62	123.18±14.34	1.5×10^(-1)
DBP (mmHg)	69.10±10.74	78.13±10.86	1.5×10^(-7)
BMI (kg/m^2)	22.78±2.59	26.21±3.12	2.6×10^(-12)
TC (mmol/L)	4.13±0.89	3.78±1.02	9.9×10^(-2)
TG (mmol/L)	1.20±0.64	2.16±1.07	3.3×10^(-7)
LDL-C (mmol/L)	2.38±0.74	2.30±1.09	3.3×10^(-1)
HDL-C (mmol/L)	1.28±0.31	1.00±0.46	9.3×10^(-11)
Glucose (mmol/L)	4.58±0.70	6.16±1.92	1.4×10^(-14)

Data are mean ± SD or number (%). SBP, systolic blood pressure; DBP, diastolic blood pressure; BMI, body mass index; TC, total cholesterol; TG, triglycerides; LDL-C, low-density lipoprotein cholesterol; HDL-C, high-density lipoprotein cholesterol.

Supplemental Table 4. Clinical characteristics of the patients used for isolating adipose exosomes.

	Patient 1	Patient 2	Patient 3	Patient 4	Patient 5	Patient 6
Age (years)	53	82	54	76	65	65
Sex	Female	Male	Male	Female	Male	Male
BMI (kg/m^2)	28.47	29.07	27.00	22.81	22.10	23.44
SBP/DBP (mmHg)	159/133	116/74	130/86	116/73	132/84	109/64
CAD	Y	Y	Y	N	N	N
Hypertension	Y	Y	N	Y	N	Y
Diabetes	Y	N	N	N	N	N
Diagnosis	SSS	SA	III°AVB	SSS	III°AVB	SSS
TC (mmol/L)	4.51	4.33	2.85	3.69	4.18	2.99
TG (mmol/L)	2.67	1.34	1.64	1.0	1.28	1.16
LDL-C (mmol/L)	2.74	2.85	1.65	2.08	2.35	1.52
HDL-C (mmol/L)	1.02	0.93	0.66	1.03	1.11	1.14
HbA1c (%)	7.8	6.0	5.9	6.0	6.0	5.7

CAD, coronary artery disease; HbA1c, glycosylated hemoglobin, type A1c; SSS, sick sinus syndrome; SA, sinus arrest; AVB, atrio-ventricular block.

Supplemental Table 5. Clinical characteristics of the patients used in Figure 3H.

	Patient 1	Patient 2	Patient 3
Age (years)	71	60	75
Sex	Male	Male	Female
BMI (kg/m^2)	24.67	25.83	25.95
SBP/DBP (mmHg)	130/80	211/82	137/76
Surgery	CEA	CEA	CEA
Hypertension	N	Y	Y
Diabetes	N	Y	N
Diagnosis	Stroke	Stroke	Stroke
TC (mmol/L)	2.04	4.08	3.33
TG (mmol/L)	0.68	1.43	1.75
LDL-C (mmol/L)	0.93	2.73	1.74
HDL-C (mmol/L)	0.67	0.89	0.97
Glucose (mmol/L)	7.5	5.45	3.07

CEA, carotid endarterectomy.

Supplemental Table 6. Clinical characteristics of the patients and healthy controls in the discovery cohort used in Figure 3.

	Healthy controls (N=29)	CAD patients (N=29)	*p*-value
Age (years)	52.39±15.88	59.92±10.37	5.0×10^(-2)
Sex (male)	13 (44.83%)	19 (65.52%)	1.9×10^(-1)
SBP (mmHg)	121.61±12.46	129.84±20.91	1.4×10^(-1)
DBP (mmHg)	71.75±11.27	71.00±9.92	5.4×10^(-1)
BMI (kg/m^2)	22.68±2.11	25.82±2.90	3.3×10^(-5)
TC (mmol/L)	4.29±1.12	3.86±0.89	1.5×10^(-1)
TG (mmol/L)	1.45±0.76	1.79±1.12	3.1×10^(-1)
LDL-C (mmol/L)	2.50±0.85	2.27±0.75	3.4×10^(-1)
HDL-C (mmol/L)	1.17±0.35	0.95±0.22	1.0×10^(-2)
HbA1c (%)	5.50±0.40	6.02±0.76	7.0×10^(-3)

Data are mean ± SD or number (%).

Supplemental Table 7. Clinical characteristics of the patients and healthy controls in the validation cohort used in Figure 3.

	Healthy controls (N=22)	CAD patients (N=22)	*p*-value
Age (years)	62.05±12.73	66.50±7.74	0.19
Sex (male)	9 (40.91%)	13 (59.09%)	0.37
SBP (mmHg)	125.05±16.76	132.50±15.01	0.13
DBP (mmHg)	77.86±13.38	79.27±13.48	0.73
BMI (kg/m^2)	25.04±2.77	24.75±3.10	0.75
TC (mmol/L)	3.75±0.78	4.05±1.21	0.35
TG (mmol/L)	1.15±0.34	1.71±0.97	0.07
LDL-C (mmol/L)	2.23±0.60	2.43±1.05	0.46
HDL-C (mmol/L)	1.11±0.26	1.06±0.29	0.56
HbA1c (%)	5.53±0.59	5.69±0.57	0.18

Data are mean ± SD or number (%).

Supplemental Table 8. Elevated metabolites in HAECs infected with Ad-*SLC7A11* versus Ad-Null.

Metabolite	Fold.Change	*P*-value
β-(m-hydroxyphenyl)hydracrylic acid	6.414656	0.033071
Fructose 6-phosphate	2.923852	0.023516
Palmitoylcarnitine	2.651698	0.003406
Glucose 6-phosphate	2.553975	0.007284
Carnitine(C0)	2.438637	0.00279
Butyrylcarnitine	2.254381	0.001079
Propionylcarnitine	2.218658	0.007628
N-Acetyl-L-aspartic acid	2.13266	0.024714
L-Glutamine	1.904755	0.001973
Tauro-ω-muricholic acid	1.848827	0.004781
O-Acetylserine	1.837886	0.012994
N-Methyl-D-aspartic acid	1.824312	0.006865
N-Acetylserine	1.822761	0.021124
L-Pipecolic acid	1.814734	0.000414
Indole-3-carboxaldehyde	1.783162	0.01912
N-Acetylglutamine	1.741226	0.004577
Erythronic acid	1.714812	0.003179
L-Serine	1.714409	0.018628
L-Proline	1.563503	0.007907
Pyroglutamic acid	1.558168	0.002058
D-Xylose	1.53004	0.000358
L-Malic acid	1.519403	0.032054
Glycine	1.490975	0.020336
Stearylcarnitine (C18)	1.415856	0.01954
L-Threonine	1.375075	0.024692
L-Phenylalanine	1.35419	0.009933
Isovalerylglycine	1.325124	0.024171
L-Asparagine	1.310267	0.009095
4-Acetamidobutanoic acid	1.265906	0.012173

Supplemental Table 9. Statistical information.

Figure	Sample sizes	Statistical tests	Post hoc correction	p-values
1C	n_1=85<br>n_2=85	Mann-Whitney *U*		*p*=8.7×10^(-12)
1D	n_1=85<br>n_2=85	Wilson/Brown		*p*=8.6×10^(-12)
1E	n_1=33<br>n_2=38<br>n_3=14	Kruskal-Wallis	Dunn	NW vs. OW, *p*=8.9×10^(-3);<br>NW vs.Obese, *p*=3.9×10^(-5);<br>OW vs.Obese, *p*=8.7×10^(-2).
1F	x=73<br>y=73	Spearman		BMI, *p*=5.4×10^(-3);<br>TG, *p*=8.9×10^(-2);<br>LDL-C, *p*=4.3×10^(-2).
1F	x=70<br>y=70	Spearman		HDL-C, *p*=2.1×10^(-1)
1J	n_1=9<br>n_2=9	Mann-Whitney *U*		Adipose tissue (Exosome), *p*=4.1×10^(-5);<br>Adipose tissue (HAEC), *p*=8.0×10^(-4);<br>Serum (Exosome), *p*=4.1×10^(-5);<br>Serum (HAEC), *p*=2.0×10^(-4).
1K	n_1=6<br>n_2=6	Mann-Whitney *U*		WAT, *p*=2.2×10^(-3);<br>BAT, *p*=9.7×10^(-1);<br>Exosome, *p*=2.2×10^(-3);<br>Aorta, *p*=4.3×10^(-3).
1L	n_1=6<br>n_2=6	Mann-Whitney *U*		WAT, *p*=2.2×10^(-3);<br>BAT, *p*=4.9×10^(-1);<br>Exosome, *p*=2.2×10^(-3);<br>Aorta, *p*=4.3×10^(-3).
1M	n_1=6<br>n_2=6	Mann-Whitney *U*		WAT, *p*=4.3×10^(-3);<br>BAT, *p*=5.9×10^(-1);<br>Exosome, *p*=2.2×10^(-2);<br>Aorta, *p*=8.7×10^(-3).
2A	n_1=3<br>n_2=3	Hypergeometric test	Benjamini-Hochberg	Nitric oxide biosynthetic process：<br>Log(*p*-value) = -5.3,<br>Log(q-value) = -3.3;<br>Glutathione metabolic process:<br>Log(*p*-value) = -2.9,<br>Log(q-value) = -1.4;<br>Regulation of fat differentiation:<br>Log(*p*-value) = -4.0,<br>Log(q-value) = -2.2;<br>Cellular oxidant detoxification:<br>Log(*p*-value) = -2.6,<br>Log(q-value) = -1.2;<br>Lipid and atherosclerosis:<br>Log(*p*-value) = -4.9,<br>Log(q-value) = -2.9;

Figure	Sample sizes	Statistical tests	Post hoc correction	p-values
2A	n_1=3<br>n_2=3	Hypergeometric test	Benjamini-Hochberg	SLC-mediated transmembrane transport:<br>Log(*p*-value) = -4.2,<br>Log(q-value) = -2.4.
2E	n_1=6<br>n_2=6	Mann-Whitney *U*		WT, *p*=2.2×10^(-3);<br>MT, *p*>9.9×10^(-1);
2F	n_1=6<br>n_2=6	Mann-Whitney *U*		*p*=2.2×10^(-3)
2G	n_1=6<br>n_2=6	Mann-Whitney *U*		*p*=4.3×10^(-3)
2H	n_1=6<br>n_2=6	Mann-Whitney *U*		Ago1 (miR-30e-5p), *p*=2.2×10^(-3);<br>Ago2 (miR-30e-5p), *p*=2.2×10^(-3);<br>Ago1 (SLC7A11), *p*=2.2×10^(-3);<br>Ago2 (SLC7A11), *p*=4.3×10^(-3).
2I	n_1=3<br>n_2=3<br>n_3=3	Hypergeometric test	Benjamini-Hochberg	D-glutamine and D-glutamate metabolism：<br>*p*-value=1.5×10^(-1),<br>q-value=1.2×10^(-1);<br>Valine, leucine and isoleucine biosynthesis:<br>*p*-value=1.0×10^(-1),<br>q-value=1.5×10^(-1);<br>Phenylalanine, tyrosine and tryptophan biosynthesis:<br>*p*-value=1.0×10^(-1),<br>q-value=1.5×10^(-1);<br>Pantothenate and CoA biosynthesis:<br>*p*-value=3.8×10^(-2),<br>q-value=7.6×10^(-2);<br>Alanine, aspartate and glutamate metabolism:<br>*p*-value=5.6×10^(-3),<br>q-value=1.7×10^(-2);<br>Glutathione metabolism:<br>*p*-value=2.5×10^(-3),<br>q-value=1.5×10^(-2).
2J	n_1=3<br>n_2=3<br>n_3=3	Kruskal-Wallis	Dunn	Glutamate (NC vs. Pal):<br>*p*=5.1×10^(-2);<br>Glutamate (NC vs. Pal+inhibitor):<br>*p*=3.0×10^(-1);<br>Glutamate (Pal vs. Pal+inhibitor):<br>*p*>9.9×10^(-1);<br>Cystine (NC vs. Pal):<br>*p*=2.2×10^(-2);

Figure	Sample sizes	Statistical tests	Post hoc correction	p-values
2J	n_1=3<br>n_2=3<br>n_3=3	Kruskal-Wallis	Dunn	Cystine (NC vs. Pal+inhibitor): *p*=5.4×10^(-1);<br>Cystine (Pal vs. Pal+inhibitor): *p*=5.4×10^(-1);<br>Glutamate/cystine (NC vs. Pal): *p*=2.2×10^(-2);<br>Glutamate/cystine (NC vs. Pal+inhibitor): *p*=5.4×10^(-1);<br>Glutamate/cystine (Pal vs. Pal+inhibitor): *p*=5.4×10^(-1).
2K	n_1=6<br>n_2=6	Mann-Whitney *U*		Glutamate, *p*=2.2×10^(-3);<br>Cystine, *p*=2.2×10^(-3);<br>Glutamate/cystine, *p*=2.2×10^(-3).
2L	n_1=8<br>n_2=8<br>n_3=8	Kruskal-Wallis	Dunn	HC serum+NC inhibitor vs. CAD serum<br>+NC inhibitor, *p*=1.2×10^(-3);<br>HC serum+NC inhibitor vs. CAD serum +30e inhibitor, *p*=4.4×10^(-1);<br>CAD serum+NC inhibitor vs. CAD serum +30e inhibitor, *p*=1.1×10^(-1).
2M	n_1=8<br>n_2=8<br>n_3=8	Kruskal-Wallis	Dunn	NC+Ad-Null vs. 30e+Ad-Null, *p*=3.0×10^(-5);<br>NC+Ad-Null vs. 30e+Ad-SLC7A11, *p*=5.3×10^(-2);<br>30e+Ad-Null vs. 30e+Ad-SLC7A11, *p*=1.2×10^(-1).
3F (mRNA)	n_1=7<br>n_2=7	Mann-Whitney *U*		*p*=7.0×10^(-3)
3F (Protein)	n_1=8<br>n_2=8	Mann-Whitney *U*		*p*=3.0×10^(-3)
3G (mRNA)	n_1=5<br>n_2=5	Mann-Whitney *U*		*p*=7.9×10^(-3)
3G (Protein)	n_1=7<br>n_2=9	Mann-Whitney *U*		*p*=7.0×10^(-4)
3H	n_1=6<br>n_2=6	Mann-Whitney *U*		*p*=2.2×10^(-3)
3I	n_1=29<br>n_2=29	Hypergeometric test	Benjamini-Hochberg	Alanine, aspartate and glutamate metabolism：<br>Log(*p*-value) = -2.20,<br>Log(q-value) = -1.78;<br>Cysteine and methionine metabolism:<br>Log(*p*-value) = -4.03,<br>Log(q-value) = -2.57;<br>Glutathione metabolism:<br>Log(*p*-value) = -1.39,<br>Log(q-value) = -1.30;

Figure	Sample sizes	Statistical tests	Post hoc correction	p-values
3I	n_1=29<br>n_2=29	Hypergeometric test	Benjamini-Hochberg	Glyoxylate and dicarboxylate metabolism:<br>Log(*p*-value) = -2.67,<br>Log(q-value) = -2.00;<br>Arginine and proline metabolism:<br>Log(*p*-value) = -1.84,<br>Log(q-value) = -1.55;<br>Arginine biosynthesis:<br>Log(*p*-value) = -2.41,<br>Log(q-value) = -1.85;<br>Histidine metabolism:<br>Log(*p*-value) = -1.74,<br>Log(q-value) = -1.51;<br>Porphyrin metabolism:<br>Log(*p*-value) = -1.39,<br>Log(q-value) = -1.29.
3J	n_1=29<br>n_2=29	Mann-Whitney *U*		Glutamate, *p*=2.0×10^(-2);<br>Cystine, *p*=5.2×10^(-6).
3K	n_1=29<br>n_2=29	Spearman		*p*=2.5×10^(-3)
3L	n_1=22<br>n_2=22	Student *t*-test		Glutamate, *p*=2.7×10^(-2);<br>Cystine, *p*=7.6×10^(-2).
3M	n_1=22<br>n_2=22	Pearson		*p*=2.5×10^(-3)
3N (Discovery)	n_1=29<br>n_2=29	Wilson/Brown		*p*=2.0×10^(-2)
3N (Validation)	n_1=22<br>n_2=22	Wilson/Brown		*p*=3.3×10^(-2)
4B	n_1=4<br>n_2=4	Hypergeometric test	Benjamini-Hochberg	Central carbon metabolism in cancer：<br>Log(*p*-value) = -18.88,<br>Log(q-value) = -18.10;<br>Carbon metabolism:<br>Log(*p*-value) = -6.62,<br>Log(q-value) = -6.54;<br>ABC transporters:<br>Log(*p*-value) = -9.66,<br>Log(q-value) = -9.48;<br>Biosynthesis of amino acids:<br>Log(*p*-value) = -11.75,<br>Log(q-value) = -11.45;<br>Aminoacyl-tRNA biosynthesis:<br>Log(*p*-value) = -12.69,<br>Log(q-value) = -12.21;<br>Alanine, aspartate and glutamate metabolism:<br>Log(*p*-value) = -5.28,<br>Log(q-value) = -5.27.

Figure	Sample sizes	Statistical tests	Post hoc correction	p-values
4C	n_1=9<br>n_2=9	Mann-Whitney *U*		Glycolysis, *p*=1.9×10^(-3);<br>Glyco Cap, *p*=8.0×10^(-4);<br>Glyco Res, *p*=2.0×10^(-1).
4D	n_1=6<br>n_2=6	Mann-Whitney *U*		ATP Pro, *p*=1.3×10^(-1);<br>Max Resp, *p*=8.7×10^(-3);<br>Spare Resp Cap, *p*=6.5×10^(-2).
4E (Glycolysis)	n_1=6<br>n_2=6<br>n_3=6	Kruskal-Wallis	Dunn	NC+Ad-Null vs. miR-30e-5p+Ad-Null, *p*=6.0×10^(-2);<br>NC+Ad-Null vs. miR-30e-5p+Ad-*SLC7A11*, *p*=6.4×10^(-1);<br>miR-30e-5p+Ad-Null vs. miR-30e-5p+Ad-*SLC7A11*, *p*=8.4×10^(-1).
4E (Glyco Cap)	n_1=6<br>n_2=6<br>n_3=6	Kruskal-Wallis	Dunn	NC+Ad-Null vs. miR-30e-5p+Ad-Null, *p*=8.5×10^(-2);<br>NC+Ad-Null vs. miR-30e-5p+Ad-*SLC7A11*, *p*>9.9×10^(-1);<br>miR-30e-5p+Ad-Null vs. miR-30e-5p+Ad-*SLC7A11*, *p*=3.6×10^(-2).
4F (Max Resp)	n_1=6<br>n_2=6<br>n_3=9	Kruskal-Wallis	Dunn	NC+Ad-Null vs. miR-30e-5p+Ad-Null, *p*=1.3×10^(-3);<br>NC+Ad-Null vs. miR-30e-5p+Ad-*SLC7A11*, *p*=7.3×10^(-2);<br>miR-30e-5p+Ad-Null vs. miR-30e-5p+Ad-*SLC7A11*, *p*=3.3×10^(-1).
4F (Spare Resp Cap)	n_1=6<br>n_2=6<br>n_3=9	Kruskal-Wallis	Dunn	NC+Ad-Null vs. miR-30e-5p+Ad-Null, *p*=5.0×10^(-4);<br>NC+Ad-Null vs. miR-30e-5p+Ad-*SLC7A11*, *p*=1.6×10^(-1);<br>miR-30e-5p+Ad-Null vs. miR-30e-5p+Ad-*SLC7A11*, *p*=1.1×10^(-1).
4G (Number)	n_1=6<br>n_2=6<br>n_3=6	Kruskal-Wallis	Dunn	NC+Ad-Null vs. 30e+Ad-Null, *p*=1.8×10^(-2);<br>NC+Ad-Null vs. 30e+Ad-*SLC7A11*, *p*>9.9×10^(-1);<br>30e+Ad-Null vs. 30e+Ad-*SLC7A11*, *p*=1.8×10^(-2).
4G (Individuals)	n_1=6<br>n_2=6<br>n_3=6	Kruskal-Wallis	Dunn	NC+Ad-Null vs. 30e+Ad-Null, *p*=1.0×10^(-1);<br>NC+Ad-Null vs. 30e+Ad-*SLC7A11*, *p*>9.9×10^(-1);<br>30e+Ad-Null vs. 30e+Ad-*SLC7A11*, *p*=1.0×10^(-2).
4G (Networks)	n_1=6<br>n_2=6<br>n_3=6	Kruskal-Wallis	Dunn	NC+Ad-Null vs. 30e+Ad-Null, *p*=1.0×10^(-1);<br>NC+Ad-Null vs. 30e+Ad-*SLC7A11*, *p*>9.9×10^(-1);<br>30e+Ad-Null vs. 30e+Ad-*SLC7A11*, *p*=1.0×10^(-2).

Figure	Sample sizes	Statistical tests	Post hoc correction	p-values
4H	$n_1=6$<br>$n_2=6$	Mann-Whitney *U*		SLC7A11, *p*=2.2×10^(-3);<br>FN1, *p*=1.5×10^(-2);<br>SNAI1, *p*=4.3×10^(-3);<br>VWF, *p*=3.8×10^(-1);<br>CDH5, *p*=4.3×10^(-3);<br>NR2F2, *p*=4.3×10^(-3).
4I	$n_1=5$<br>$n_2=5$	Mann-Whitney *U*		*p*=7.9×10^(-3)
4J	$n_1=6$<br>$n_2=6$<br>$n_3=6$	Kruskal-Wallis	Dunn	NC+Ad-Null vs. miR-30e-5p+Ad-Null, *p*=2.9×10^(-3);<br>NC+Ad-Null vs. miR-30e-5p+Ad-SLC7A11, *p*>9.9×10^(-1);<br>miR-30e-5p+Ad-Null vs. miR-30e-5p+Ad-SLC7A11, *p*=5.2×10^(-2).
5B	$n_1=5$<br>$n_2=5$	Mann-Whitney *U*		*p*=1.6×10^(-2)
5E	$n_1=7$<br>$n_2=7$<br>$n_3=7$	Kruskal-Wallis	Dunn	NC vs. miR-30e, *p*=4.3×10^(-3);<br>NC vs. miR-30e+GW, *p*=5.0×10^(-3);<br>miR-30e vs. miR-30e+GW, *p*>9.9×10^(-1).
5G	$n_1=7$<br>$n_2=7$<br>$n_3=7$	Kruskal-Wallis	Dunn	NC vs. miR-30e, *p*=2.7×10^(-3);<br>NC vs. miR-30e+GW, *p*=7.7×10^(-3);<br>miR-30e vs. miR-30e+GW, *p*>9.9×10^(-1).
5H	$n_1=7$<br>$n_2=7$<br>$n_3=7$	Kruskal-Wallis	Dunn	NC vs. miR-30e, *p*=9.0×10^(-4);<br>NC vs. miR-30e+GW, *p*=1.6×10^(-1);<br>miR-30e vs. miR-30e+GW, *p*=2.8×10^(-1).
5I	$n_1=6$<br>$n_2=6$<br>$n_3=6$	Kruskal-Wallis	Dunn	NC vs. miR-30e, *p*=2.9×10^(-3);<br>NC vs. miR-30e+GW, *p*>9.9×10^(-1);<br>miR-30e vs. miR-30e+GW, *p*=3.3×10^(-2).
5K	$n_1=7$<br>$n_2=7$<br>$n_3=7$	Kruskal-Wallis	Dunn	NC vs. miR-30e, *p*=5.0×10^(-4);<br>NC vs. miR-30e+GW, *p*=8.4×10^(-2);<br>miR-30e vs. miR-30e+GW, *p*=3.6×10^(-1).
5L (Slc7a11 protein)	$n_1=6$<br>$n_2=6$<br>$n_3=6$	Kruskal-Wallis	Dunn	NC vs. miR-30e, *p*=3.0×10^(-4);<br>NC vs. miR-30e+GW, *p*=1.6×10^(-1);<br>miR-30e vs. miR-30e+GW, *p*=1.6×10^(-1).
5L (Slc7a11 mRNA)	$n_1=6$<br>$n_2=6$<br>$n_3=6$	Kruskal-Wallis	Dunn	NC vs. miR-30e, *p*=5.1×10^(-3);<br>NC vs. miR-30e+GW, *p*>9.9×10^(-1);

Figure	Sample sizes	Statistical tests	Post hoc correction	p-values
5L (Slc7a11 mRNA)	n_1=6<br>n_2=6<br>n_3=6	Kruskal-Wallis	Dunn	miR-30e vs. miR-30e+GW, p=2.1×10^(-2).
5M (T-CHO)	n_1=7<br>n_2=7<br>n_3=7	Kruskal-Wallis	Dunn	NC vs. miR-30e, p=5.5×10^(-1); NC vs. miR-30e+GW, p=9.7×10^(-1); miR-30e vs. miR-30e+GW, p>9.9×10^(-1).
5M (TG)	n_1=7<br>n_2=7<br>n_3=7	Kruskal-Wallis	Dunn	NC vs. miR-30e, p>9.9×10^(-1); NC vs. miR-30e+GW, p>9.9×10^(-1); miR-30e vs. miR-30e+GW, p>9.9×10^(-1).
5M (LDL-C)	n_1=7<br>n_2=7<br>n_3=7	Kruskal-Wallis	Dunn	NC vs. miR-30e, p=9.7×10^(-1); NC vs. miR-30e+GW, p>9.9×10^(-1); miR-30e vs. miR-30e+GW, p>9.9×10^(-1).
5N	n_1=7<br>n_2=7<br>n_3=7	Kruskal-Wallis	Dunn	NC vs. miR-30e, p=3.0×10^(-4); NC vs. miR-30e+GW, p=4.7×10^(-1); miR-30e vs. miR-30e+GW, p=4.2×10^(-2).
5O	n_1=7<br>n_2=7<br>n_3=7	Kruskal-Wallis	Dunn	NC vs. miR-30e, p=8.0×10^(-4); NC vs. miR-30e+GW, p=5.0×10^(-1); miR-30e vs. miR-30e+GW, p=6.7×10^(-2).
5P	n_1=7<br>n_2=7<br>n_3=7	Kruskal-Wallis	Dunn	NC vs. miR-30e, p=1.2×10^(-3); NC vs. miR-30e+GW, p>9.9×10^(-1); miR-30e vs. miR-30e+GW, p=1.5×10^(-2).
5Q (Glycolysis)	n_1=6<br>n_2=6<br>n_3=6	Kruskal-Wallis	Dunn	NC vs. miR-30e, p=3.0×10^(-4); NC vs. miR-30e+GW, p=1.5×10^(-1); miR-30e vs. miR-30e+GW, p=1.5×10^(-1).
5Q (Glyco Cap)	n_1=6<br>n_2=6<br>n_3=6	Kruskal-Wallis	Dunn	NC vs. miR-30e, p=1.5×10^(-2); NC vs. miR-30e+GW, p>9.9×10^(-1); miR-30e vs. miR-30e+GW, p=5.2×10^(-2).
5R (Max Resp)	n_1=6<br>n_2=6<br>n_3=6	Kruskal-Wallis	Dunn	NC vs. miR-30e, p=5.1×10^(-3); NC vs. miR-30e+GW, p>9.9×10^(-1); miR-30e vs. miR-30e+GW, p=3.3×10^(-2).
**Figure**	**Sample sizes**	**Statistical tests**	**Post hoc correction**	**p-values**

5R<br>(Spare Rsep Cap)	n_1=6<br>n_2=6<br>n_3=6	Kruskal-Wallis	Dunn	NC vs. miR-30e, p=4.5×10^(-2);<br>NC vs. miR-30e+GW, p>9.9×10^(-1);<br>miR-30e vs. miR-30e+GW, p=4.5×10^(-2).
6B	n_1=6<br>n_2=6<br>n_3=6	Kruskal-Wallis	Dunn	NC vs. anti-miR-30e, p=1.6×10^(-3);<br>NC vs. GW4869, p=5.2×10^(-2);<br>anti-miR-30e vs. GW4869, p=8.4×10^(-1).
6D	n_1=5<br>n_2=5<br>n_3=5	Kruskal-Wallis	Dunn	NC vs. anti-miR-30e, p=3.3×10^(-2);<br>NC vs. GW4869, p>9.9×10^(-1);<br>anti-miR-30e vs. GW4869, p=1.8×10^(-2).
6E	n_1=6<br>n_2=6<br>n_3=6	Kruskal-Wallis	Dunn	NC vs. anti-miR-30e, p=8.0×10^(-4);<br>NC vs. GW4869, p=1.5×10^(-1);<br>anti-miR-30e vs. GW4869, p=2.7×10^(-1).
6F	n_1=6<br>n_2=6<br>n_3=6	Kruskal-Wallis	Dunn	NC vs. anti-miR-30e, p=2.4×10^(-3);<br>NC vs. GW4869, p=3.9×10^(-2);<br>anti-miR-30e vs. GW4869, p>9.9×10^(-1).
6H	n_1=6<br>n_2=6<br>n_3=6	Kruskal-Wallis	Dunn	NC vs. anti-miR-30e, p=5.9×10^(-2);<br>NC vs. GW4869, p=8.9×10^(-3);<br>anti-miR-30e vs. GW4869, p>9.9×10^(-1).
6I<br>(Slc7a11 protein)	n_1=5<br>n_2=5<br>n_3=5	Kruskal-Wallis	Dunn	NC vs. anti-miR-30e, p=1.8×10^(-2);<br>NC vs. GW4869, p=3.3×10^(-2);<br>anti-miR-30e vs. GW4869, p>9.9×10^(-1).
6I<br>(Slc7a11 mRNA)	n_1=6<br>n_2=6<br>n_3=6	Kruskal-Wallis	Dunn	NC vs. anti-miR-30e, p=1.1×10^(-2);<br>NC vs. GW4869, p=1.1×10^(-2);<br>anti-miR-30e vs. GW4869, p>9.9×10^(-1).
6J<br>(T-CHO)	n_1=6<br>n_2=6<br>n_3=6	Kruskal-Wallis	Dunn	NC vs. anti-miR-30e, p=9.5×10^(-1);<br>NC vs. GW4869, p>9.9×10^(-1);<br>anti-miR-30e vs. GW4869, p>9.9×10^(-1).
6J<br>(TG)	n_1=6<br>n_2=6<br>n_3=6	Kruskal-Wallis	Dunn	NC vs. anti-miR-30e, p>9.9×10^(-1);<br>NC vs. GW4869, p>9.9×10^(-1);<br>anti-miR-30e vs. GW4869, p=5.8×10^(-1).

**Figure**	**Sample sizes**	**Statistical tests**	**Post hoc correction**	**p-values**
6J	n_1=6	Kruskal-Wallis	Dunn	NC vs. anti-miR-30e, p=3.9×10^(-

(LDL-C)	n_2=6<br>n_3=6			1);<br>NC vs. GW4869, *p*=3.5×10^(-1);<br>anti-miR-30e vs. GW4869, *p*>9.9×10^(-1).
6K	n_1=6<br>n_2=6<br>n_3=6	Kruskal-Wallis	Dunn	NC vs. anti-miR-30e, *p*=3.5×10^(-3);<br>NC vs. GW4869, *p*=4.5×10^(-2);<br>anti-miR-30e vs. GW4869, *p*>9.9×10^(-1).
6L	n_1=6<br>n_2=6<br>n_3=6	Kruskal-Wallis	Dunn	NC vs. anti-miR-30e, *p*=9.2×10^(-2);<br>NC vs. GW4869, *p*=4.3×10^(-3);<br>anti-miR-30e vs. GW4869, *p*=9.1×10^(-1).
6M	n_1=6<br>n_2=6<br>n_3=6	Kruskal-Wallis	Dunn	NC vs. anti-miR-30e, *p*=3.9×10^(-2);<br>NC vs. GW4869, *p*=2.4×10^(-3);<br>anti-miR-30e vs. GW4869, *p*>9.9×10^(-1).
6N (Glycolysis)	n_1=6<br>n_2=6<br>n_3=6	Kruskal-Wallis	Dunn	NC vs. anti-miR-30e, *p*=2.2×10^(-1);<br>NC vs. GW4869, *p*=1.1×10^(-3);<br>anti-miR-30e vs. GW4869, *p*=2.2×10^(-1).
6N (Glyco Cap)	n_1=6<br>n_2=6<br>n_3=6	Kruskal-Wallis	Dunn	NC vs. anti-miR-30e, *p*=3.9×10^(-2);<br>NC vs. GW4869, *p*=2.4×10^(-3);<br>anti-miR-30e vs. GW4869, *p*>9.9×10^(-1).
6O (Max Resp)	n_1=6<br>n_2=6<br>n_3=6	Kruskal-Wallis	Dunn	NC vs. anti-miR-30e, *p*=6.0×10^(-2);<br>NC vs. GW4869, *p*=1.3×10^(-2);<br>anti-miR-30e vs. GW4869, *p*>9.9×10^(-1).
6O (Spare Rsep Cap)	n_1=6<br>n_2=6<br>n_3=6	Kruskal-Wallis	Dunn	NC vs. anti-miR-30e, *p*=1.8×10^(-2);<br>NC vs. GW4869, *p*=1.1×10^(-2);<br>anti-miR-30e vs. GW4869, *p*>9.9×10^(-1).
S1A	x=85<br>y=85	Spearman		BMI, *p*=1.0×10^(-6);<br>TG, *p*=2.4×10^(-2);<br>LDL-C, *p*=2.3×10^(-3);<br>HDL-C, *p*=4.1×10^(-2).
S2B	n_1=6<br>n_2=6	Mann-Whitney *U*		miR-30e-5p, *p*=2.2×10^(-3);<br>SLC7A11, *p*=2.2×10^(-3).
S2C	n_1=5<br>n_2=5	Mann-Whitney *U*		miR-30e-5p, *p*=5.6×10^(-2);<br>SLC7A11, *p*=7.9×10^(-3).
S2D	n_1=6<br>n_2=6<br>n_3=6	Kruskal-Wallis	Dunn	Exosome (NC vs. Pal), *p*=4.5×10^(-2);
**Figure**	**Sample sizes**	**Statistical tests**	**Post hoc correction**	**p-values**

S2D	n_1=6<br>n_2=6<br>n_3=6	Kruskal-Wallis	Dunn	Exosome (NC vs. Pal+inhibitor), *p*=9.9×10^(-1);<br>Exosome (Pal vs. Pal+inhibitor), *p*=2×10^(-3);<br>HAEC (NC vs. Pal), *p*=1.5×10^(-1);<br>HAEC (NC vs. Pal+inhibitor), *p*=1.5×10^(-1);<br>HAEC (Pal vs. Pal+inhibitor), *p*=3.0×10^(-4).
S4A	n_1=8<br>n_2=6	Mann-Whitney *U*		Glycolysis, *p*=7.0×10^(-4);<br>Glyco Cap, *p*=7.0×10^(-4);<br>Glyco Res, *p*=8.1×10^(-2).
S4B	n_1=6<br>n_2=10	Mann-Whitney *U*		ATP Pro, *p*=1.0×10^(-3);<br>Max Resp, *p*=2.0×10^(-4);<br>Spare Resp Cap, *p*=2.0×10^(-4).
S4C	n_1=12<br>n_2=12	Mann-Whitney *U*		Glycolysis, *p*=2.2×10^(-5);<br>Glyco Cap, *p*=8.9×10^(-6);
S4C	n_1=12<br>n_2=12	Student *t*-test		Glyco Res, *p*=2.0×10^(-4).
S4D	n_1=6<br>n_2=8	Mann-Whitney *U*		ATP Pro, *p*=7.0×10^(-4);<br>Max Resp, *p*=7.0×10^(-4);<br>Spare Resp Cap, *p*=1.3×10^(-3).
S4E	n_1=6<br>n_2=6	Mann-Whitney *U*		*p*=2.2×10^(-3)
S4F	n_1=5<br>n_2=5	Mann-Whitney *U*		*p*=7.9×10^(-3)
S4G	n_1=5<br>n_2=5	Mann-Whitney *U*		*p*=1.6×10^(-2)
S4H	n_1=5<br>n_2=5	Mann-Whitney *U*		SLC7A11, *p*=1.6×10^(-2);<br>$peNOS^{Ser1177}$, *p*=7.9×10^(-3);<br>eNOS, *p*=9.5×10^(-2).
S4I	n_1=6<br>n_2=6	Mann-Whitney *U*		*p*=2.2×10^(-3)
S5C (Male)	n_1=6<br>n_2=6	Mann-Whitney *U*		TC, *p*=7.0×10^(-1);<br>TG, *p*=2.0×10^(-1);<br>LDL-C, *p*=9.4×10^(-1);<br>HDL-C, *p*=7.3×10^(-1).
S5C (Female)	n_1=6<br>n_2=5	Mann-Whitney *U*		TC, *p*=2.5×10^(-1);<br>TG, *p*=2.3×10^(-1);<br>LDL-C, *p*=2.5×10^(-1);<br>HDL-C, *p*=1.6×10^(-1).
S5D (Male)	n_1=6<br>n_2=6	Mann-Whitney *U*		*p*=2.2×10^(-3)
S5D (Female)	n_1=6<br>n_2=5	Mann-Whitney *U*		*p*=4.3×10^(-3)
S5E (Male)	n_1=5<br>n_2=5	Mann-Whitney *U*		Lesion area, *p*=9.5×10^(-2)
S5E (Male)	n_1=6<br>n_2=6	Mann-Whitney *U*		Fluorescent intensity, *p*=2.2×10^(-3)
**Figure**	**Sample sizes**	**Statistical tests**	**Post hoc correction**	**p-values**
S5E	n_1=6	Mann-Whitney *U*		Lesion area, *p*=2.2×10^(-3)

(Female)	n_2=5			
S5E (Female)	n_1=5 n_2=5	Mann-Whitney *U*		Fluorescent intensity, *p*=7.9×10^(-3)
S5F	n_1=6 n_2=6	Mann-Whitney *U*		Glutamate, *p*=2.2×10^(-3); Cystine, *p*=2.2×10^(-3); Glutamate/ Cystine, *p*=2.2×10^(-3).
S5G	n_1=6 n_2=5	Mann-Whitney *U*		Glutamate, *p*=4.3×10^(-3); Cystine, *p*=4.3×10^(-3); Glutamate/ Cystine, *p*=4.3×10^(-3).
S6B	n_1=6 n_2=6	Mann-Whitney *U*		Serum, *p*=8.7×10^(-3); Aorta, *p*=2.2×10^(-3).
S6C	n_1=6 n_2=6	Mann-Whitney *U*		*p*=2.2×10^(-3)
S6D	n_1=6 n_2=6	Mann-Whitney *U*		LDL-C, *p*=3.9×10^(-1); T-CHO, *p*=7.0×10^(-1); TG, *p*=9.4×10^(-1).
S6E	n_1=6 n_2=6	Mann-Whitney *U*		*p*=2.2×10^(-3)
S6F	n_1=6 n_2=6	Mann-Whitney *U*		*p*=2.2×10^(-3)
S6G	n_1=6 n_2=6	Mann-Whitney *U*		*p*=2.2×10^(-3)
S7B	n_1=8 n_2=8	Mann-Whitney *U*		*p*=4.7×10^(-3)
S7C	n_1=8 n_2=8	Mann-Whitney *U*		*p*=2.0×10^(-4)
S7D (Serum)	n_1=7 n_2=7	Mann-Whitney *U*		*p*=1.8×10^(-2)
S7D (Aorta)	n_1=6 n_2=6	Mann-Whitney *U*		*p*=2.2×10^(-3)
S7E	n_1=6 n_2=6	Mann-Whitney *U*		*p*=2.2×10^(-3)
S7F	n_1=8 n_2=11	Mann-Whitney *U*		*p*=3.0×10^(-4)
S7G	n_1=7 n_2=10	Mann-Whitney *U*		*p*=4.0×10^(-4)
S7H	n_1=8 n_2=11	Mann-Whitney *U*		*p*=5.1×10^(-2)
S7J	n_1=8 n_2=10	Mann-Whitney *U*		TC, *p*=5.7×10^(-1); TG, *p*=8.5×10^(-1); LDL-C, *p*=5.7×10^(-1); HDL-C, *p*=9.8×10^(-1).
S7K	n_1=8 n_2=8	Mann-Whitney *U*		Glutamate, *p*=2.7×10^(-2); Cystine, *p*=6.1×10^(-2); Glutamate/ Cystine, *p*=2.0×10^(-4).
S8A (PBS/TNFα)	n_1=8 n_2=8	Mann-Whitney *U*		*p*=2.0×10^(-4)

**Figure**	**Sample sizes**	**Statistical tests**	**Post hoc correction**	**p-values**
S8A	n_1=5	Mann-Whitney *U*		*p*=2.4×10^(-2)

(PS/OS)	$n_2=5$		
S8A (TA/AA)	$n_1=7$ $n_2=7$	Mann-Whitney *U*	$p=1.8\times10^{(-2)}$

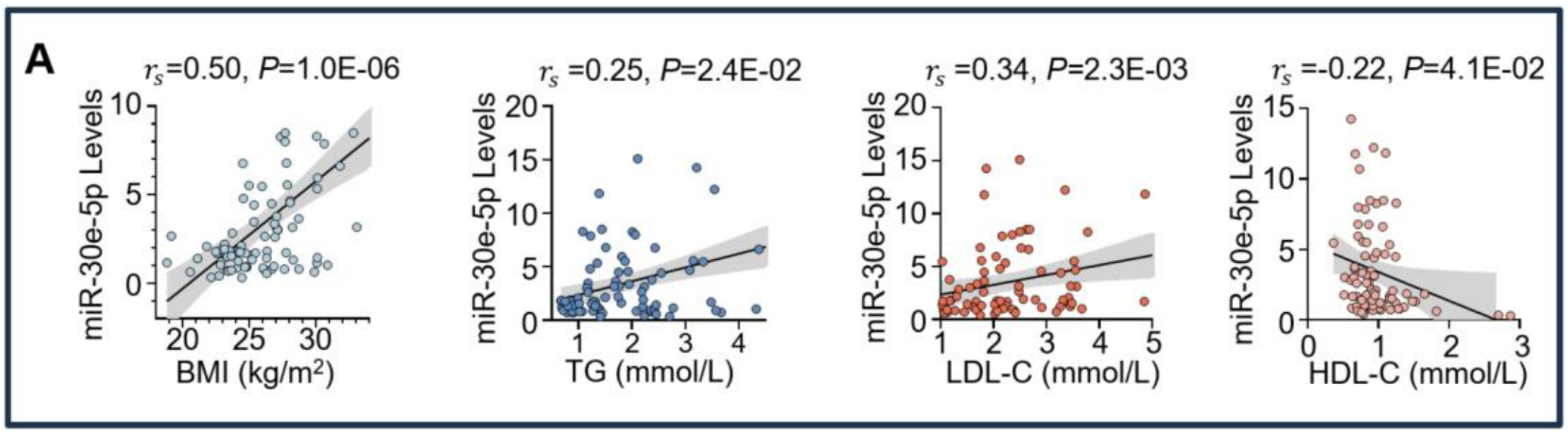

Supplemental Figure 1. (A) Serum was collected from CAD patients (n=85) and healthy controls (HCs) (n=85) registered in the First Affiliated Hospital of Xi'an Jiaotong University. Serum level of miR-30e-5p was measured by qPCR. The data are fold-change normalized to the mean level of HCs. Spearman's rank correlations between miR-30-5p level and BMI, TG, LDL-C, and HDL-C in CAD patients.

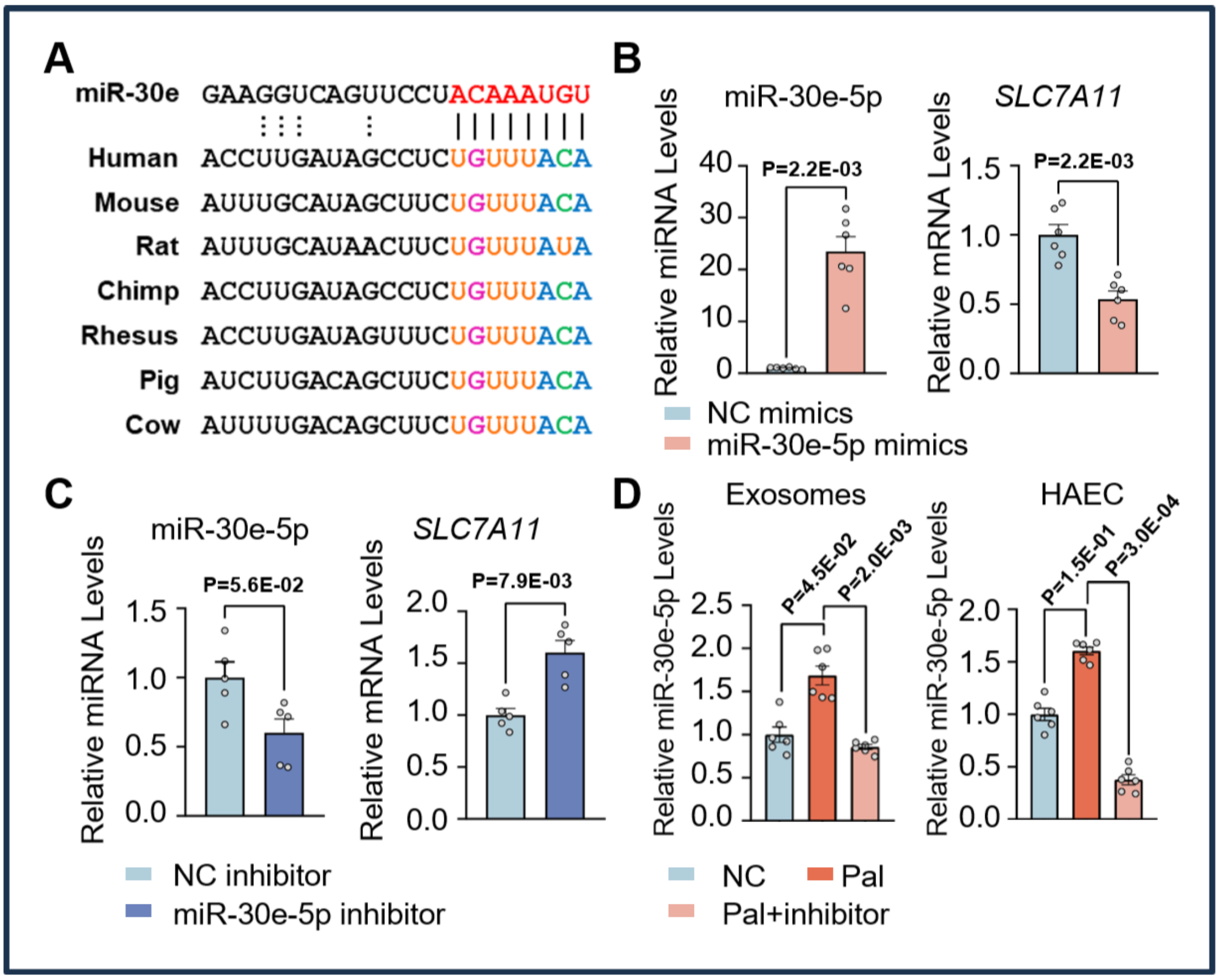

Supplemental Figure 2. (**A**) TargetScan prediction of miR-30e-5p binding sites in the 3′-UTR of *SLC7A11* mRNA in mammalian species as indicated. (**B)** HAECs were transfected with miR-30e-5p or control miRNA (n=6). (**C**) HAECs were transfected with miR-30e-5p inhibitor (Anti-30e) or control miRNA. All cells were lysed and levels of miR-30e-5p and *SLC7A11* were measured by qPCR (n=5). (**D**) 3T3L1-derived adipocytes were incubated with or without palmitate (400 mM) for 24 hr (Pal; NC). The palmitate-incubated adipocytes were also transfected with miR-30e-5p inhibitor (Pal+inhibitor). HAECs were incubated with exosomes isolated from the 3 groups of adipocytes. Levels of miR-30e-5p in exosomes and HAECs were measured (n=6). Data were analyzed by Mann-Whitney *U* test between the 2 indicated groups (B, C). Data were analyzed by Kruskal-Wallis with Dunnett's post *hoc* test among multiple groups

(D). The exact *p*-values, statistical tests, post *hoc* corrections, and sample sizes (n) are listed in the Table S9.

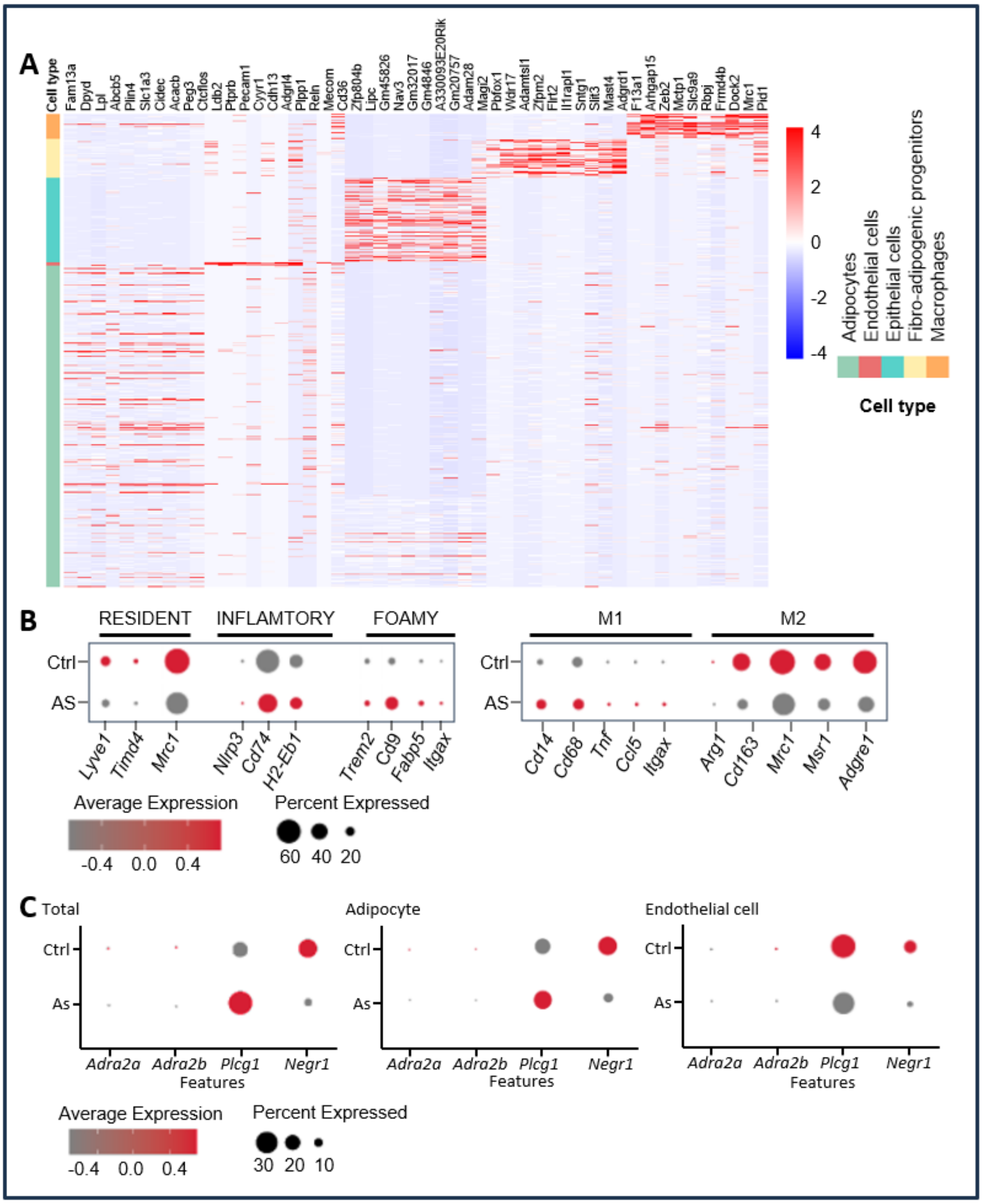

Supplemental Figure 3. (**A-C**) Eight-week-old male *Apoe*$^{-/-}$ mice were fed an HFD or chow diet for 12 weeks. eWAT were collected from 3 animals in each of the two groups for snRNA-seq analysis. (**A**) Heatmap showing the top 10 marker genes in each of the 5 cell types. The expression levels are shown from high (red) to low (blue). (**B**) Dot plots showing the expression of marker genes of the diversed macrophage subtypes. (**C**)

Dot plots showing the expression pattern of *Adra2a*, *Adra2b*, *Plcg1*, and *Negr1* in total cells and annotated adipocyte and EC subgroups. Color scale represents relative expression levels; grey: low, red: high.

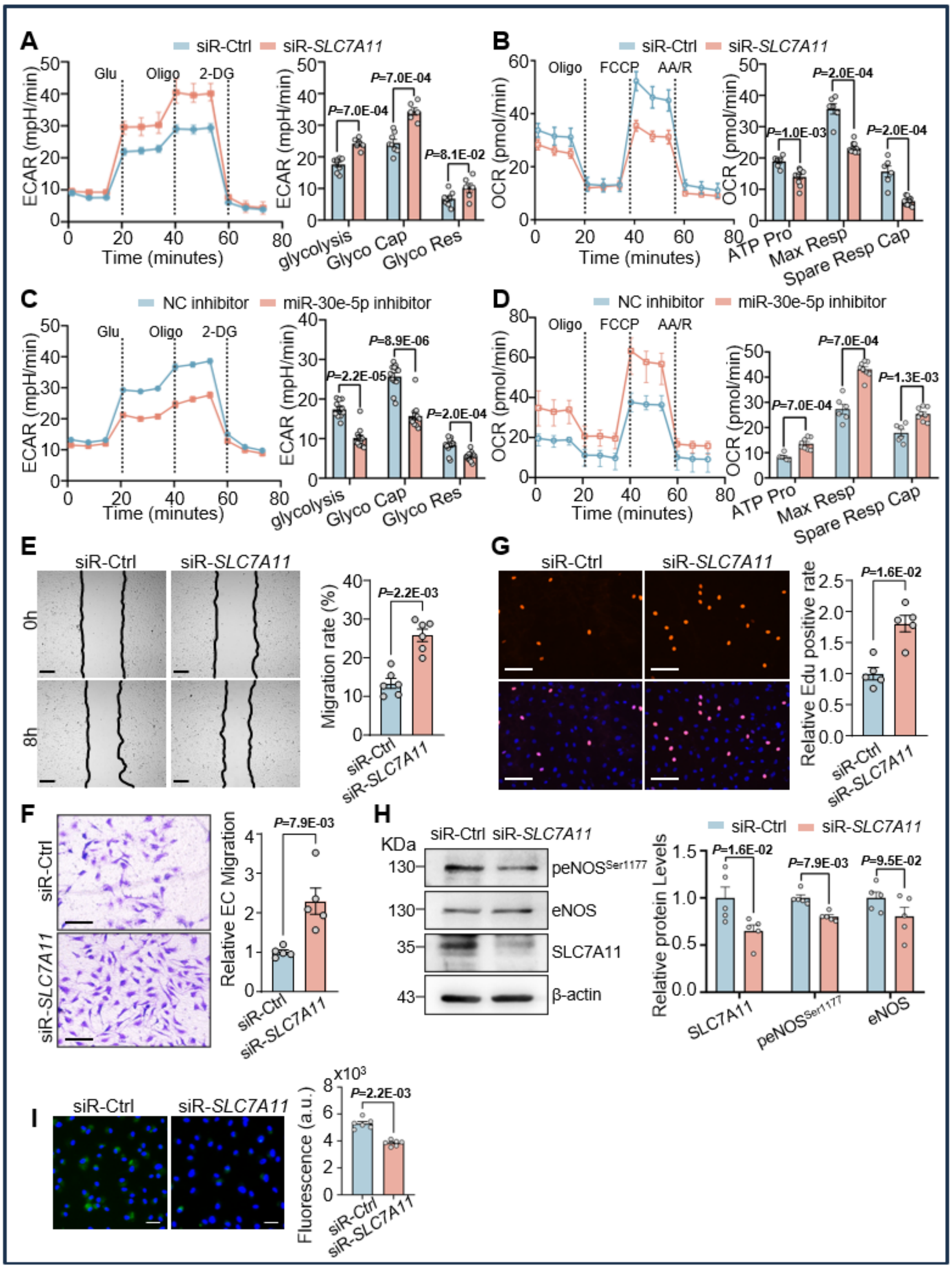

Supplemental Figure 4. (**A-D**) Extracellular acidification rate (ECAR) and oxygen consumption rate (OCR) assays of HAECs transfected with siR-*SLC7A11* (n=6 for ECAR; n=10 for OCR) or siR-Ctrl (n=8 for ECAR; n=6 for OCR) (A,B); and with miR-30e-5p inhibitor (n=12 for ECAR; n=8 for OCR) or NC miRNA (n=12 for ECAR; n=6 for OCR) (C,D). (**E-I**) HAECs were transfected with siR-*SLC7A11* or siR-Ctrl.

Migration was measured by wound healing (E, n=6, scale bar=100 μm) and trans-well assays (F, n=5, scale bar=50 μm); proliferation was evaluated by EdU staining (G, n=5, scale bar=50 μm); and protein levels of SLC7A11, eNOS, p-eNOS S1177, and β-actin were detected by western blot analysis (H, n=5). The level of intracellular nitric oxide was evaluated using the NO (DAF-FM DA) assay kit (I, n=6); representative immunofluorescence images of NO (green) and DAPI-labeled nuclei (blue) in SLC7A11 knockdown and control cells (left, scale bar=50 μm); quantification of NO fluorescence intensity by a fluorescence spectrophotometer (right, ex. 495 nm and em. 515 nm). Data were analyzed by Student *t*-test [Glyco Res in (C)] or Mann-Whitney *U* test [A, B, Glycolysis and Glyco Cap in (C), D-I] between the 2 indicated groups. The exact *p*-values, statistical tests, post *hoc* corrections, and sample sizes (n) are listed in the Table S9.

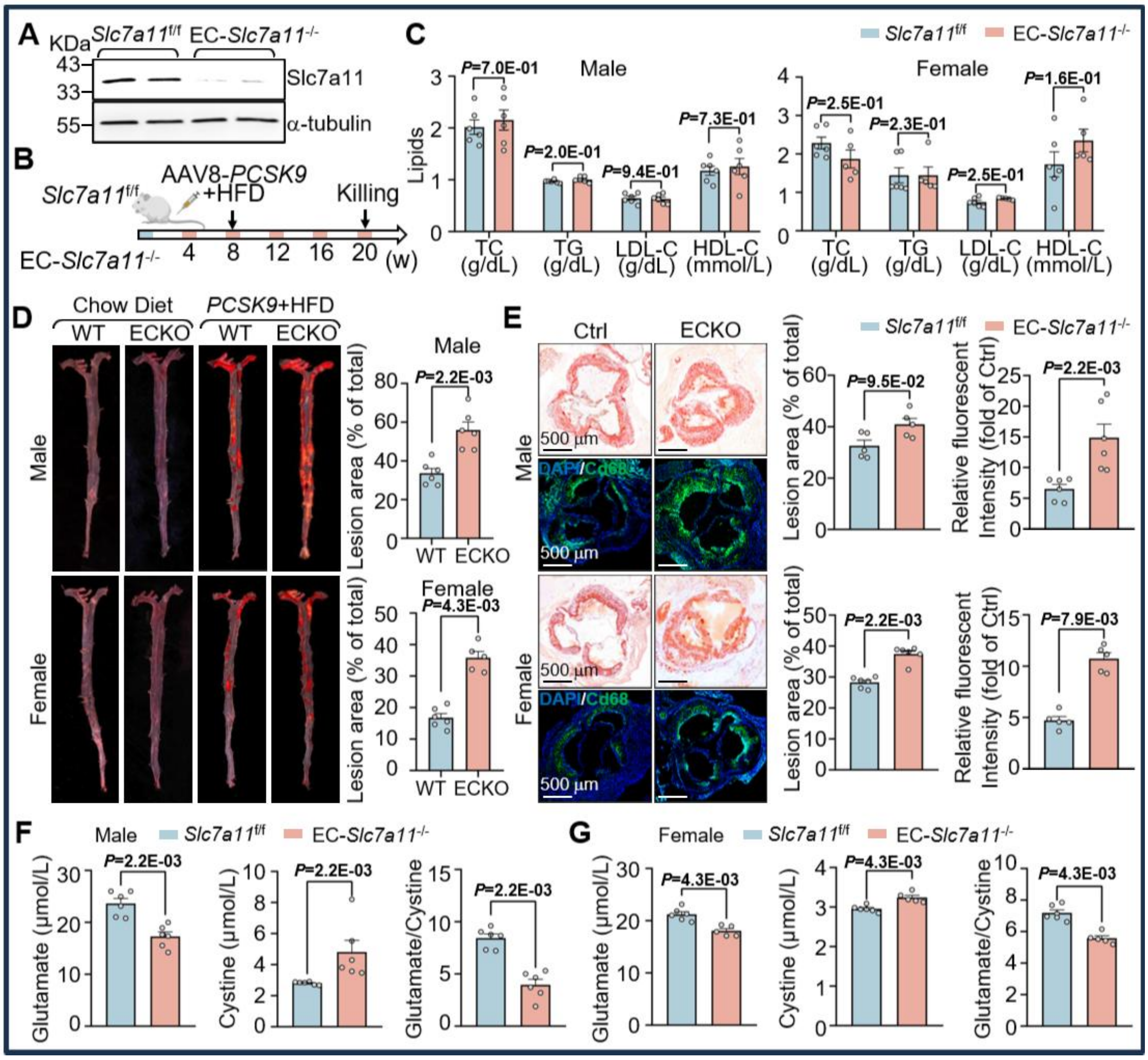

Supplemental Figure 5. EC-specific *Slc7a11* deficiency aggravates atherosclerosis in mice. (**A-B**) Western blot analysis showing Slc7a11 and α-tubulin in lung ECs from EC-*Slc7a11*$^{-/-}$ and their *Slc7a11*$^{flox/flox}$ control littermates. (**B**) Eight-week-old male and female EC-*Slc7a11*$^{-/-}$ and their *Slc7a11*$^{flox/flox}$ littermates were administered a single dose of AAV8-*PCSK9*^{D377Y} (1×10^{11} vg/mouse) via tail vein injection and fed an HFD for 12 weeks. (**C**) Levels of serum TC, TG, LDL-C, and HDL-C in the two groups of mice (n=6 males and 6 females in *Slc7a11*$^{flox/flox}$ group; n=6 males and 5 females in EC-*Slc7a11*$^{-/-}$ group). (**D**) *En face* Oil-red O staining of aortic specimens (n=6 males and 6 females in *Slc7a11*$^{flox/flox}$ group; n=6 males and 5 females in EC-*Slc7a11*$^{-/-}$ group). (**E**) Histological sections of aortic roots stained with Oil-red O (atherosclerosis) (n=5 males

and 6 females in *Slc7a11*$^{flox/flox}$ group; n=5 males and 5 females in EC-*Slc7a11*$^{-/-}$ group), or immunostaining with anti-Cd68 antibody (macrophage, green). The specimens were counterstained with DAPI (blue) (n=6 males and 5 females per group). Scale bars: 500 μm. (**F,G**) Levels of serum glutamate and cystine and their ratios in the two groups of mice (n=6 in F; n=6 *Slc7a11*$^{flox/flox}$ and 5 EC-*Slc7a11*$^{-/-}$ mice in G). Data were analyzed by Mann-Whitney *U* test between the 2 indicated groups (C-G). The exact *p*-values, statistical tests, post *hoc* corrections, and sample sizes (n) are listed in the Table S9.

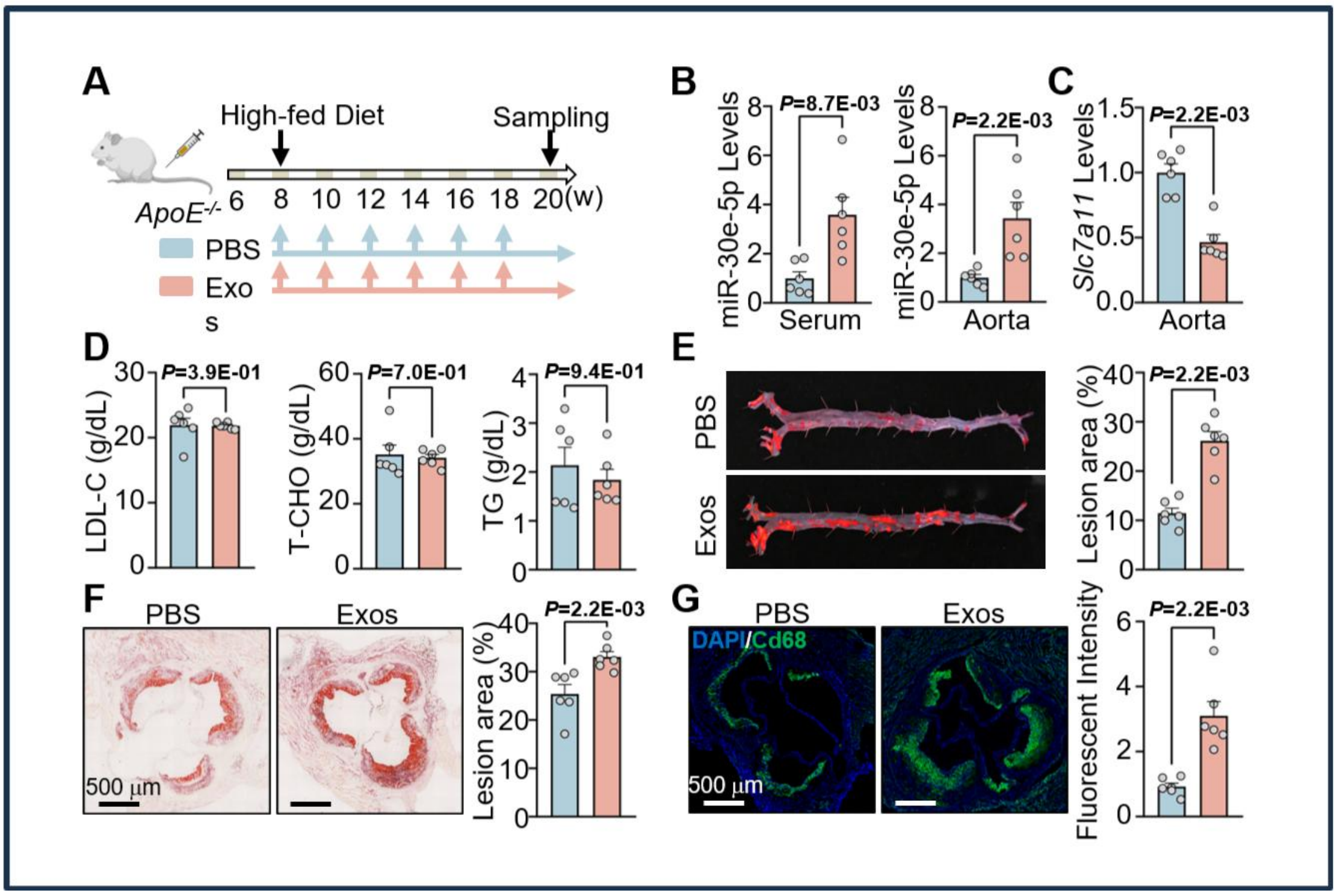

Supplemental Figure 6. (**A**) At 8 weeks old, male *Apoe*$^{-/-}$ mice fed with HFD received exosomes derived from WAT of *ob*/*ob* mice (Exos) at 2 mg/kg/2 wk or PBS via tail-vein injection for 12 weeks. (**B**) Levels of miR-30e-5p in serum and aortic intima; (**C**) *Slc7a11* mRNA; (**D**) TC, TG, and LDL-C; (**E**) *en face* Oil-red O staining of atherosclerotic lesions; (**F-G**) Oil-red O and anti-Cd68 immunostaining of the aortic root of the 2 groups of mice. Data are mean±SEM from 6 mice per group. Data were analyzed by Mann-Whitney *U* test (B-G). The exact *p*-values, statistical tests, post *hoc* corrections, and sample sizes (n) are listed in the Table S9.

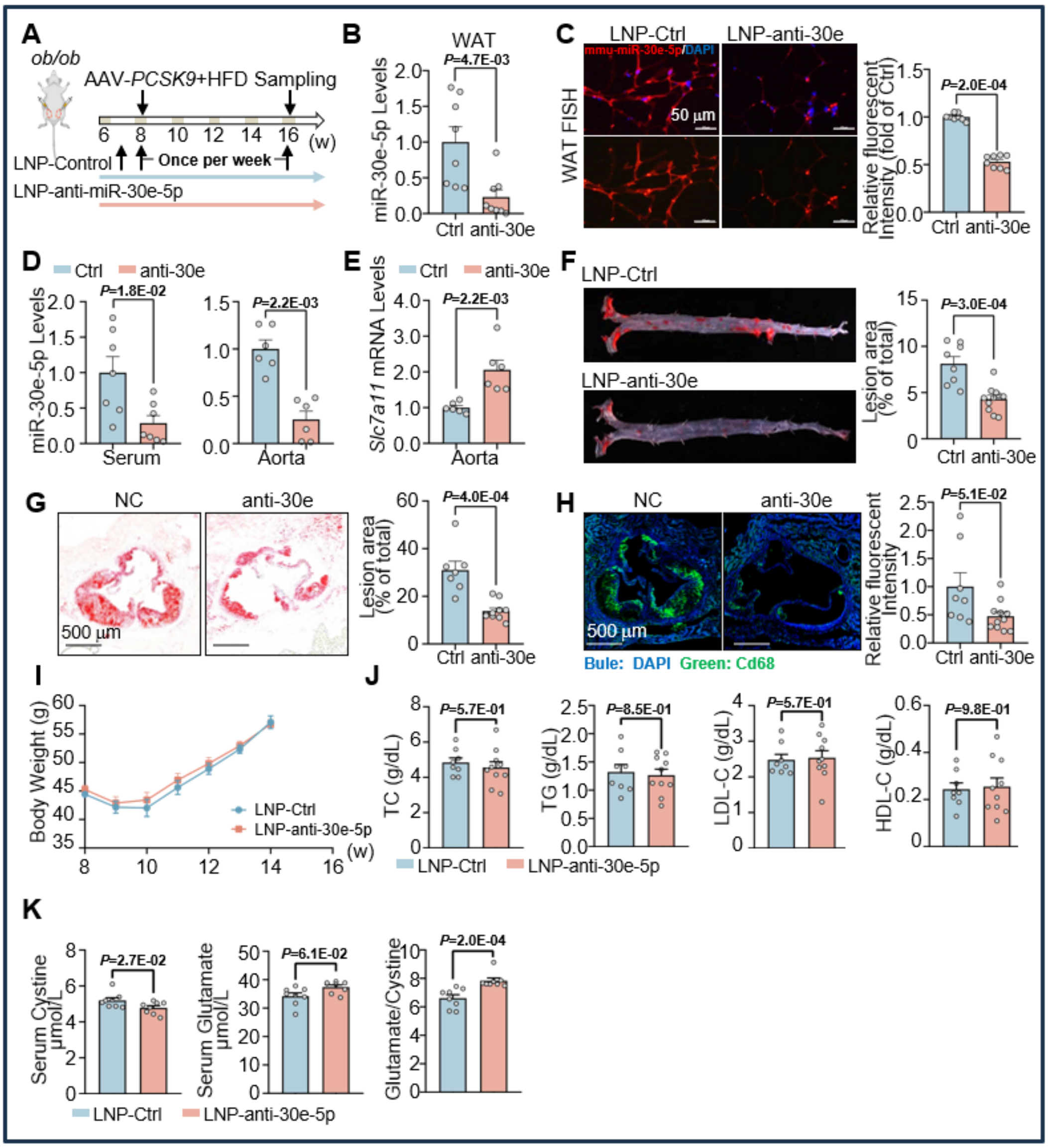

Supplemental Figure 7. Atherosclerosis reduction by LNP-miR-30e-5p antagonism. (**A**) At 7 weeks old, male *ob/ob* mice received 2 mg/kg/wk LNP-anti-miR-30e-5p or LNP-control miRNA (LNP-Ctrl) in WAT in the inguinal area. One week later, all mice were administered AAV8-*PCSK9*^{D377Y} and fed an HFD to induce hyperlipidemia. (**B-K**) Levels of miR-30e-5p in WAT by qPCR (B, n=8) and FISH (C, n=8) and in serum (n=7) and aortic intima (n=6) (D); level of *Slc7a11* mRNA in mouse aortic intima (E, n=6); Oil-red O staining of atherosclerosis in aortic specimens (n=8 for LNP-Ctrl group, n=11 for LNP-anti-miR-30e-5p group) and aortic root (n=7 for

LNP-Ctrl, n=10 for LNP-anti-miR-30e-5p) (F,G); anti-Cd68 immunostaining (H; n=8 for LNP-Ctrl, n=11 for LNP-anti-miR-30e-5p); body weights (I; n=8 for LNP-Ctrl, n=11 for LNP-anti-miR-30e-5p); levels of serum TC, TG, LDL-C, and HDL-C (J, n=8 for LNP-Ctrl, n=10 for LNP-anti-miR-30e-5p); serum levels of glutamate and cystine and their ratios (K, n=8). Data were analyzed by Mann-Whitney *U* test between the 2 indicated groups (B-H, J, K). The exact *p*-values, statistical tests, post *hoc* corrections, and sample sizes (n) are listed in the Table S9.

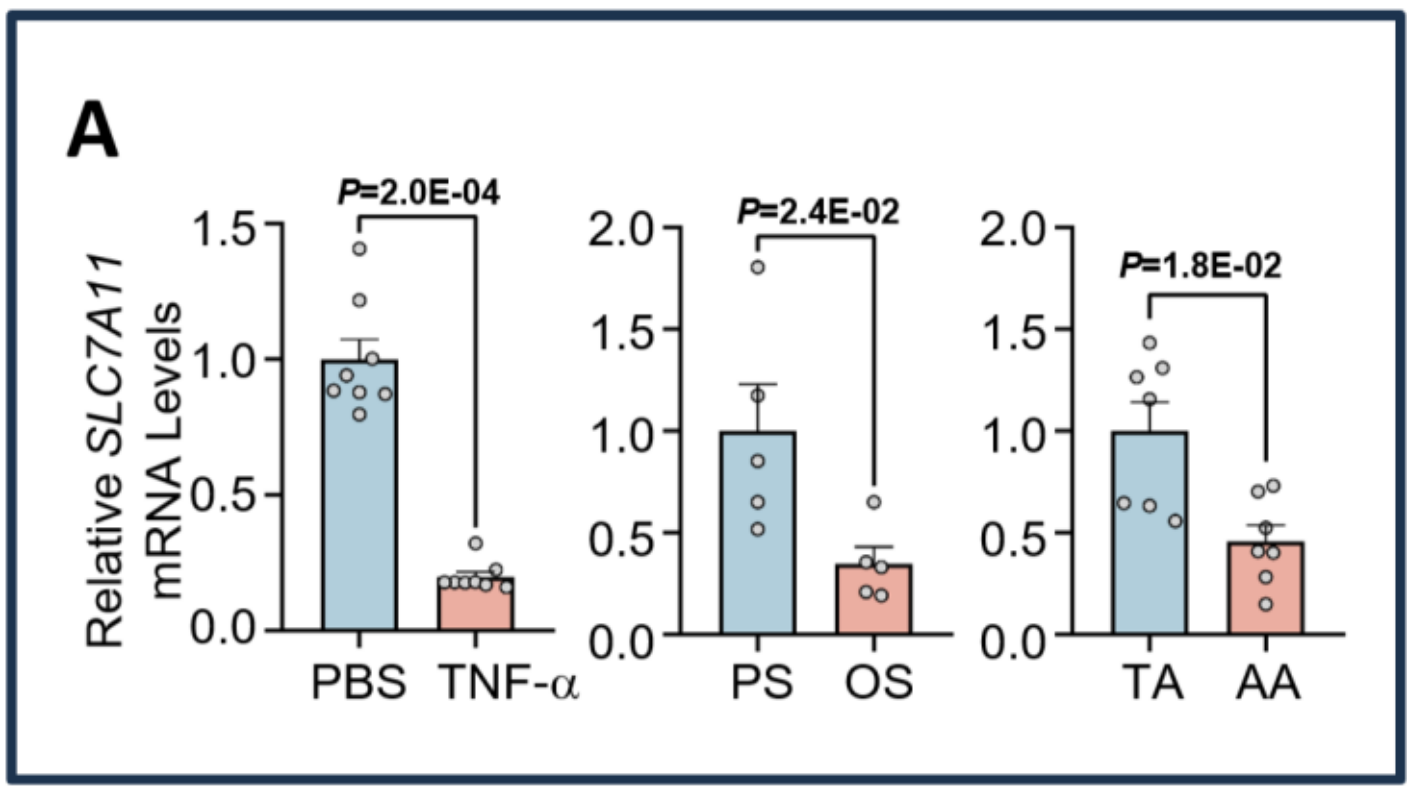

Supplemental Figure 8. (**A**) HAECs were treated with TNFα (10 ng/mL) or PBS as controls for 48 hr (n=8, 2 technological replicates from 4 different batches of HAECs). In a separate experimental set, HAECs were exposed to oscillatory shear stress (OS: 0.5±4 dyn/cm^2) or pulsatile shear stress (PS: 12±4 dyn/cm^2) for 16 h (n=5). The thoracic aorta (TA) or aortic arch (AA) was isolated from C57BL/6 mice (n=7). The expression of *SLC7A11* was measured by qPCR. Data were analyzed by Mann-Whitney *U* test between the 2 indicated groups.

Major Resources Table

In order to allow validation and replication of experiments, all essential research materials listed in the Methods should be included in the Major Resources Table below. Authors are encouraged to use public repositories for protocols, data, code, and other materials and provide persistent identifiers and/or links to repositories when available. Authors may add or delete rows as needed.

Mouse Models

Strain	Vendor or Source	Background Strain	Sex	Other Information (e.g. breeding scheme for F1, F2…if applicable)	Persistent ID / URL
C57BL/6J WT Mice	Xi'an Jiaotong University	C57BL/6J	M	Mice were obtained directly from Xi'an Jiaotong University at 7-wk of age (no in-house breeding).	
Apoe$^{-/-}$	Cyagen Biosciences Inc.	C57BL/6J	M	Mice were purchased directly from Cyagen at 7-wk of age (no in-house breeding).	https://www.cyagen.cn/mice-products/C001507
ob/ob	Beijing HFK Bioscience Co., Ltd.	C57BL/6J	M	Mice were purchased directly from Cyagen at 7-wk of age (no in-house breeding).	http://www.hfkbio.com/cms/item/view?table=product&id=52
Slc7a11 flox/flox	Cyagen Biosciences Inc.	C57BL/6J	F	This strain was purchased for mouse breeding to generate endothelial cell-specific deletion of *Slc7a11.*	https://www.cyagen.cn/mice-bank/S-CKO-09630
VE-Cad-Cre-ERT2	GemPharmatech Co., Ltd.	C57BL/6J	M	This strain was purchased for mouse breeding to generate endothelial cell-specific deletion of *Slc7a11.*	https://cn.gempharmatech.com/shop/productDetails/60569
Slc7a11$^{flox/flox}$ VE-Cad-Cre-ERT2 (EC-*Slc7a11*$^{-/-}$)	In house	C57BL/6J	M&F	Breeding Scheme:<br>F0: Female *Slc7a11*$^{flox/flox}$ x Male VE-Cad-*Cre-ERT2*$^{+/0}$<br>F1: Female *Slc7a11*$^{flox/+}$ VE-Cad-*Cre-ERT2*$^{0/0}$ x Male *Slc7a11*$^{flox/+}$ VE-Cad-*Cre-ERT2*$^{+/0}$<br>F2: Female *Slc7a11*$^{flox/flox}$ VE-Cad-*Cre-ERT2*$^{0/0}$ x Male *Slc7a11*$^{flox/flox}$ VE-Cad-*Cre-ERT2*$^{+/0}$	

Mouse Models Used in This Study

Strain	Vendor or Source	Background Strain	Sex	Other Information (e.g. breeding scheme for F1, F2…if applicable)	Persistent ID / URL
AS (C57BL/6J WT, PCSK9+HFD)	Xi'an Jiaotong University	C57BL/6J	M	8-wk-old C57BL/6J WT mice were administered a single dose of AAV8-*PCSK9*^{D377Y} (1×10^11 vg/mouse) by tail vein injection and fed a HFD for 12 weeks.	
AS (*Slc7a11*$^{flox/flox}$ &EC-*Slc7a11*$^{-/-}$, PCSK9+HFD)	In house	C57BL/6J	M&F	8-wk-old *Slc7a11*$^{flox/flox}$ and EC-*Slc7a11*$^{-/-}$ mice were administered a single dose of AAV8-*PCSK9*^{D377Y} (1×10^11 vg/mouse) by tail vein injection and fed a HFD for 12 weeks.	
AS (*Apoe*$^{-/-}$, HFD)	Cyagen Biosciences Inc.	C57BL/6J	M	8-wk-old *Apoe*$^{-/-}$ mice were fed a HFD for 12 weeks.	
AS (*ob/ob*, PCSK9+HFD)	Beijing HFK Bioscience Co., Ltd.	C57BL/6J	M	8-wk-old *ob/ob* mice were administered a single dose of AAV8-*PCSK9*^{D377Y} (1×10^11 vg/mouse) by	

DOI [to be added]

				tail vein injection and fed a HFD for 12 weeks.	

Antibodies

Target antigen	Vendor or Source	Catalog #	Working concentration	Lot # (preferred but not required)	Persistent ID / URL
SLC7A11	Cell Signaling	12691	WB 1:800		https://www.cellsignal.cn/products/primary-antibodies/xct-slc7a11-d2m7a-rabbit-monoclonal-antibody/12691
SLC7A11	Abcam	ab307601	IF 1:400		https://www.abcam.cn/products/primary-antibodies/xct-antibody-epr27115-64-ab307601
eNOS	BD Biosciences	610296	WB 1:1000		https://www.bdbiosciences.com/zh-cn/products/reagents/western-blotting-and-molecular-reagents/western-blot-reagents/purified-mouse-anti-enos-nos-type-iii.610296?tab=product_details
p-eNOS S1177	Cell Signaling	9570	WB 1:500		https://www.cellsignal.cn/products/primary-antibodies/phospho-enos-ser1177-c9c3-rabbit-monoclonal-antibody/9570
β-actin	Santa Cruz	sc-47778	WB 1:1000		https://www.scbt.com/zh/p/beta-actin-antibody-c4
GAPDH	Abways	AB0037	WB 1:10000		https://www.abways.com/ProductsStd/AB0037.html
α-tubulin	Affinity Biosciences	AF7010	WB 1:20000		https://www.affbiotech.cn/goods-6285-AF7010-Tubulin_alpha_Antibody.html
Peroxidase-conjugated Goat Anti-Rabbit IgG	Jackson	111-035-003	WB 1:5000		https://www.jacksonimmuno.com/catalog/products/111-035-003
Peroxidase-conjugated Goat Anti-Mouse IgG	Jackson	115-035-003	WB 1:5000		https://www.jacksonimmuno.com/catalog/products/115-035-003
Ago1	Cell Signaling	5053	IP 5 μL/ 1.5×10^7 cells		https://www.cellsignal.cn/products/primary-antibodies/argonaute-1-d84g10-rabbit-monoclonal-antibody/5053
Ago2	Cell Signaling	2897	IP 5 μL/ 1.5×10^7 cells		https://www.cellsignal.cn/products/primary-antibodies/argonaute-2-c34c6-rabbit-monoclonal-antibody/2897
CD68	Abcam	ab53444	IF 1：200		https://www.abcam.cn/products/primary-antibodies/cd68-antibody-fa-11-ab53444
CD31	Invitrogen	14-0311-82	IF 1:50		https://www.thermofisher.cn/cn/zh/antibody/product/CD31-PECAM-1-Antibody-clone-390-Monoclonal/14-0311-82
Flag	Proteintech	20543-1-AP	IF 1:100		https://www.ptgcn.com/products/Flag-Tag-Antibody-20543-1-AP.htm
goat anti-rabbit Alexa Fluor 488	Servicebio	GB25303	IF 1:200		https://www.service-bio.com/goodsdetail?id=273
donkey anti-mouse Cy3	Servicebio	GB21401	IF 1:200		https://www.service-bio.com/goodsdetail?id=254

DOI [to be added]

FITC conjugated Goat Anti-Rat	Servicebio	GB22302	IF 1:400		https://www.service-bio.com/goodsdetail?id=258
Rabbit mAb IgG	Cell Signaling	3900S	IF 1:200		https://www.cellsignal.cn/products/primary-antibodies/rabbit-da1e-monoclonal-antibody-igg-isotype-control/3900
Mouse mAb IgG	Cell Signaling	61656S	IF 1:200		https://www.cellsignal.cn/products/primary-antibodies/mouse-e5y6q-monoclonal-antibody-igg2a-isotype-control/61656

DNA/cDNA/miRNA Clones

Clone Name	Sequence	Source / Repository	Persistent ID / URL
Cy3-labelled-mmu-miR-30e-5p RNA probes	5'-CUUCCAGUCAAGGAUGUUUACA-3'	Sangon Biotech	
Homo-miR-30e-5p mimics	5'-UGUAAACAUCCUUGACUGGAAG-3'<br>5'-UCCAGUCAAGGAUGUUUACAUU-3'	Genepharma	https://www.genepharma.com/en/miRNA-Mimics/
Homo-miR-mimics control	5'-UUCUCCGAACGUGUCACGUTT-3'<br>5'-ACGUGACACGUUCGGAGAATT-3'	Genepharma	https://www.genepharma.com/en/miRNA-Mimics/
Homo-miR-30e-5p inhibitor	5'-CUUCCAGUCAAGGAUGUUUACA-3'	Genepharma	https://www.genepharma.com/en/miRNA-Inhibitors/
Homo-miR-inhibitor control	5'-CAGUACUUUUGUGUAGUACAA-3'	Genepharma	https://www.genepharma.com/en/miRNA-Inhibitors/
Mus-miR-30e-5p antagomir	5'-CUUCCAGUCAAGGAUGUUUACA-3'	Genepharma	https://www.genepharma.com/en/miRNA-agomir-antagomir/
Mus-miR-antagomir control	5'-CAGUACUUUUGUGUAGUACAA-3'	Genepharma	https://www.genepharma.com/en/miRNA-agomir-antagomir/
AAV9-adiponectin-miR-30e-5p	GTACAAATAACCAGTCAGAAAACCTGTTGGAGAAGTGGGCATCCTAATTGACATTAATTCTGACCCTGCATGAGGGTTTTTGGGTTAGCAAGCCCTGGGTCACCTCCTTATGGGAGCAGGTTGTGCCTCACTGAAGCTCTTTATTTTTCTGGGCAGTCTTTGCTACTGTAAACATCCTTGACTGGAAGCTGTAAGGTGTTGAGAGGAGCTTTCAGTCGGATGTTTACAGCGGCAGGCTGCCACAGTCGTCCCCGGCTGCGCTCATCGGCTTTGAGACATTGCAGGTTTGTGCCCATGAACTTGTCTCCTTTTCTTGGGTTACTTCCTTTCTCTCTGAGAAGTCACAAGAACTCAGACTGTCTCTCTTCGTTTGGAGCTGATAATACCATGAG	HANBIO Biotechnology Co. Ltd	https://www.hanbio.net/productclass_8/productdetail_11.shtml
AAV9-adiponectin-miR-30e-5p sponge	CTTCCAGTCATCCTGTTTACATATACCTTCCAGTCATCCTGTTTACAACATCCTTCCAGTCATCCTGTTTACATCTTCACTTCCAGTCATCCTGTTTACA	HANBIO Biotechnology Co. Ltd	https://www.hanbio.net/productclass_8/productdetail_11.shtml
AAV9-adiponectin-CD63-3xFlag	gccaccATGGCGGTGGAAGGAGGAATGAAGTGTGTCAAGTTTTTGCTCTACGTTCTCCTGCTGGCCTTCTGCGCCTGTGCAGTGGGATTGATCGCCATTGGTGTAGCGGTTCAGGTTGTCTTGAAGCAGGCCATTACCCATGAGACTACTGCTGGCTCGCTGTTGCCTGTGGTCATCATTGCAGTGGGTGCCTT	HANBIO Biotechnology Co. Ltd	https://www.hanbio.net/productclass_8/productdetail_11.shtml

DOI [to be added]

	CCTCTTCCTGGTGGCCTTTGTGGGCTGC<br>TGTGGGGCCTGCAAGGAGAACTACTGT<br>CTCATGATTACATTTGCCATCTTCCTGTC<br>TCTTATCATGCTTGTGGAGGTGGCTGTG<br>GCCATTGCTGGCTATGTGTTTAGAGACC<br>AGGTGAAGTCAGAGTTTAATAAAAGCT<br>TCCAGCAGCAGATGCAGAATTACCTTAA<br>AGACAACAAAACAGCCACTATTTTGGA<br>CAAATTGCAGAAAGAAAATAACTGCTG<br>TGGAGCTTCTAACTACACAGACTGGGA<br>AAACATCCCCGGCATGGCCAAGGACAG<br>AGTCCCCGATTCTTGCTGCATCAACATA<br>ACTGTGGGCTGTGGGAATGATTTCAAG<br>GAATCCACTATCCATACCCAGGGCTGCG<br>TGGAGACTATAGCAATATGGCTAAGGA<br>AGAACATACTGCTGGTGGCTGCAGCGG<br>CCCTGGGCATTGCTTTTGTGGAGGTCTT<br>GGGAATTATCTTCTCCTGCTGTCTGGTG<br>AAGAGTATTCGAAGTGGCTATGAAGTA<br>ATGccaccggtcatggtgagcaagggcgaggagc<br>tgttcaccggggtggtgcccatcctggtcgagctgg<br>acggcgacgtaaacggccacaagttcagcgtgtcc<br>ggcgagggcgagggcgatgccacctacggcaagc<br>tgaccctgaagttcatctgcaccaccggcaagctgc<br>ccgtgccctggcccaccctcgtgaccaccctgacct<br>acggcgtgcagtgcttcagccgctaccccgaccaca<br>tgaagcagcacgacttcttcaagtccgccatgcccg<br>aaggctacgtccaggagcgcaccatcttcttcaagg<br>acgacggcaactacaagacccgcgccgaggtgaa<br>gttcgagggcgacaccctggtgaaccgcatcgagc<br>tgaagggcatcgacttcaaggaggacggcaacatc<br>ctggggcacaagctggagtacaactacaacagcca<br>caacgtctatatcatggccgacaagcagaagaacg<br>gcatcaaggtgaacttcaagatccgccacaacatc<br>gaggacggcagcgtgcagctcgccgaccactacca<br>gcagaacacccccatcggcgacggccccgtgctgc<br>tgcccgacaaccactacctgagcacccagtccgccc<br>tgagcaaagaccccaacgagaagcgcgatcacat<br>ggtcctgctggagttcgtgaccgccgccgggatcac<br>tctcggcatggacgagctgtacaag		
Ad-SLC7A11	ATGGTCAGAAAGCCTGTTGTGTCCACCA<br>TCTCCAAAGGAGGTTACCTGCAGGGAA<br>ATGTTAACGGGAGGCTGCCTTCCCTGG<br>GCAACAAGGAGCCACCTGGGCAGGAG<br>AAAGTGCAGCTGAAGAGGAAAGTCACT<br>TTACTGAGGGGAGTCTCCATTATCATTG<br>GCACCATCATTGGAGCAGGAATCTTCAT<br>CTCTCCTAAGGGCGTGCTCCAGAACAC<br>GGGCAGCGTGGGCATGTCTCTGACCAT<br>CTGGACGGTGTGTGGGGTCCTGTCACT<br>ATTTGGAGCTTTGTCTTATGCTGAATTG<br>GGAACAACTATAAAGAAATCTGGAGGT<br>CATTACACATATATTTTGGAAGTCTTTG<br>GTCCATTACCAGCTTTTGTACGAGTCTG<br>GGTGGAACTCCTCATAATACGCCCTGCA<br>GCTACTGCTGTGATATCCCTGGCATTTG<br>GACGCTACATTCTGGAACCATTTTTTATT<br>CAATGTGAAATCCCTGAACTTGCGATCA	HANBIO Biotechnology Co. Ltd	https://www.hanbio.net/productclass_7/productdetail_15.shtml

DOI [to be added]

	AGCTCATTACAGCTGTGGGCATAACTGT<br>AGTGATGGTCCTAAATAGCATGAGTGT<br>CAGCTGGAGCGCCCGGATCCAGATTTT<br>CTTAACCTTTTGCAAGCTCACAGCAATT<br>CTGATAATTATAGTCCCTGGAGTTATGC<br>AGCTAATTAAAGGTCAAACGCAGAACT<br>TTAAAGACGCCTTTTCAGGAAGAGATTC<br>AAGTATTACGCGGTTGCCACTGGCTTTT<br>TATTATGGAATGTATGCATATGCTGGCT<br>GGTTTTACCTCAACTTTGTTACTGAAGA<br>AGTAGAAAACCCTGAAAAAACCATTCC<br>CCTTGCAATATGTATATCCATGGCCATT<br>GTCACCATTGGCTATGTGCTGACAAATG<br>TGGCCTACTTTACGACCATTAATGCTGA<br>GGAGCTGCTGCTTTCAAATGCAGTGGC<br>AGTGACCTTTTCTGAGCGGCTACTGGG<br>AAATTTCTCATTAGCAGTTCCGATCTTTG<br>TTGCCCTCTCCTGCTTTGGCTCCATGAA<br>CGGTGGTGTGTTTGCTGTCTCCAGGTTA<br>TTCTATGTTGCGTCTCGAGAGGGTCACC<br>TTCCAGAAATCCTCTCCATGATTCATGTC<br>CGCAAGCACACTCCTCTACCAGCTGTTA<br>TTGTTTTGCACCCTTTGACAATGATAAT<br>GCTCTTCTCTGGAGACCTCGACAGTCTT<br>TTGAATTTCCTCAGTTTTGCCAGGTGGC<br>TTTTTATTGGGCTGGCAGTTGCTGGGCT<br>GATTTATCTTCGATACAAATGCCCAGAT<br>ATGCATCGTCCTTTCAAGGTGCCACTGT<br>TCATCCCAGCTTTGTTTTCCTTCACATGC<br>CTCTTCATGGTTGCCCTTTCCCTCTATTC<br>GGACCCATTTAGTACAGGGATTGGCTTC<br>GTCATCACTCTGACTGGAGTCCCTGCGT<br>ATTATCTCTTTATTATATGGGACAAGAA<br>ACCCAGGTGGTTTAGAATAATGTCAGA<br>GAAAATAACCAGAACATTACAAATAAT<br>ACTGGAAGTTGTACCAGAAGAAGATAA<br>GTTATGA		

Cultured Cells

Name	Vendor or Source	Sex (F, M, or unknown)	Persistent ID / URL
Human HAECs	Sciencell	M&F	https://sciencellonline.com/en/human-aortic-endothelial-cells/
Mouse aortic ECs	Generated in our Laboratory	M	
Mouse lung ECs	Generated in our Laboratory	M	
3T3-L1	ATCC	unknown	
HEK293T	ATCC	unknown	

Data & Code Availability

Description	Source / Repository	Persistent ID / URL
RNA-Seq	GEO (GSE334495)	https://www.ncbi.nlm.nih.gov/geo/query/acc.cgi?acc=GSE334495
snRNA-Seq	GEO (GSE335161)	https://www.ncbi.nlm.nih.gov/geo/query/acc.cgi?acc=GSE335161

Other

DOI [to be added]

Description	Source / Repository	Persistent ID / URL
TaqMan™ MicroRNA RT Assay Kit	Applied Biosystems	https://www.thermofisher.cn/order/catalog/product/4366596?SID=srch-srp-4366596
TaqMan™ MicroRNA Assay (Cel-39, U6, homo/mus miR-30e-5p primers)	Applied Biosystems	https://www.thermofisher.cn/order/catalog/product/4427975?SID=srch-hj-4427975
GoTaq® qPCR Master Mix	Promega	https://www.promega.com.cn/products/pcr/qpcr-and-rt-qpcr/qpcr-kits/?catNum=A6002
SLC7A11 siRNA	Santa Cruz	https://www.scbt.com/p/xct-sirna-h-shrna-and-lentiviral-particle-gene-silencers
Control siRNA	Santa Cruz	https://www.scbt.com/zh/p/control-sirna-a
Seahorse FluxPaks	Agilent	https://www.agilent.com.cn/store/productDetail.jsp?catalogId=103792-100&catId=SubCat2ECS_897068
Seahorse XF Glycolytic Rate Assay Kit	Agilent	https://www.agilent.com.cn/store/productDetail.jsp?catalogId=103344-100&catId=SubCat2ECS_897074
Seahorse XF Cell Mito Stress Test Kit	Agilent	https://www.agilent.com.cn/store/productDetail.jsp?catalogId=103015-100&catId=SubCat2ECS_897072
Cystine uptake assay kit	Dojindo Laboratories	https://www.dojindo.cn/#/goodsDetails?gid=1098
Nitric oxide assay kit	Beyotime	https://www.beyotime.com/product/S0020S.htm
Glutamate ELISA kit (human)	FANKEW	http://www.shfksc.com/cp_view.asp?id=89065
Cystine ELISA kit (human)	FANKEW	http://www.shfksc.com/cp_view.asp?id=89066
Glutamate ELISA kit (mouse)	FANKEW	http://www.shfksc.com/cp_view.asp?id=89062
Cystine ELISA kit (mouse)	FANKEW	http://www.shfksc.com/cp_view.asp?id=89064
GW4869	MCE	https://www.medchemexpress.cn/GW4869.html
ROS Assay kit	YEASEN	https://www.yeasen.com/products/detail/1038
MitoTracker Red CMXRos	Beyotime	https://www.beyotime.com/product/C1035-250%CE%BCg.htm
BeyoClick™ EdU-555 Cell Proliferation Assay Kit	Beyotime	https://www.beyotime.com/product/C0075S.htm
SYBR Green Supermix	Bio-Rad	https://www.bio-rad.com/zh-cn/sku/1725272-ssoadvanced-universal-sybr-green-supermix-1-000-x-20-ul-rxns-10-ml-10-x-1-ml?ID=1725272
Tamoxifen	Sigma-Aldrich	https://www.sigmaaldrich.cn/CN/zh/product/sigma/t5648
High-fed diet	Research Diets	https://researchdiets.com/zh-CN/formulas/d12109c

ARRIVE GUIDELINES

The ARRIVE guidelines (https://arriveguidelines.org/) are a checklist of recommendations to improve the reporting of research involving animals. Key elements of the study design should be included below to better enable readers to scrutinize the research adequately, evaluate its methodological rigor, and reproduce the methods or findings.

Study Design

Figure 5 (AAV-miR-30e-5p)

Groups	Sex	Age	Number (prior to experiment)	Number (after termination)	Littermates (Yes/No)	Other description
Group 1 (NC)	M	8-wk-old	13	13	NO (age-matched with Group 2&3)	
Group 2 (miR-30e)	M	8-wk-old	13	13	NO	
Group 2 (miR-30e+GW)	M	8-wk-old	13	13	NO	

Figure 6 (AAV-anti-miR-30e-5p/GW4869)

Groups	Sex	Age	Number (prior to experiment)	Number (after termination)	Littermates (Yes/No)	Other description
Group 1 (NC)	M	8-wk-old	12	12	NO (age-matched with Group 2&3)	
Group 2 (anti-miR-30e)	M	8-wk-old	12	12	NO	
Group 2 (GW4869)	M	8-wk-old	12	12	NO	

Figure S5 (ECKO)

Groups	Sex	Age	Number (prior to experiment)	Number (after termination)	Littermates (Yes/No)	Other description
Group 1 (Slc7a11$^{f/f}$)	M&F	8-wk-old	6	6	NO (age-matched with Group 2)	
Group 2 (EC-*Slc7a11*$^{-/-}$)	M&F	8-wk-old	6	6	NO	

Figure S6 (Exo)

Groups	Sex	Age	Number (prior to experiment)	Number (after termination)	Littermates (Yes/No)	Other description
Group 1 (PBS)	M	8-wk-old	12	12	NO (age-matched with Group 2)	
Group 2 (Exo)	M	8-wk-old	12	12	NO	

Figure S7 (LNP)

Groups	Sex	Age	Number (prior to experiment)	Number (after termination)	Littermates (Yes/No)	Other description
Group 1 (LNP-Control)	M	8-wk-old	14	14	NO (age-matched with Group 2)	

DOI [to be added]

Group 2 (LNP-anti-miR-30e-5p)	M	8-wk-old	18	17	NO	One mouse died for unknown reasons during modeling and was excluded.

Sample Size: Sample size was determined based on our previous experimental experience in atherosclerotic mouse models. No formal power analysis was performed.

Inclusion Criteria

Study animals were monitored longitudinally with histopathological assessment. Valid atherosclerotic model was confirmed by detectable aortic plaques and increased macrophage infiltration, accompanied by elevated serum lipid profiles.

Exclusion Criteria

No animal was excluded from analysis.

Randomization

We used stratified block randomization with a block size of 4 mice for two-group designs and 6 mice for three-group designs to keep animal number balanced. All animals were enrolled in a blinded fashion with allocation concealment via third-party-assigned ear tag numbers.

Blinding

All animal procedures, in vivo morphometric and functional data collections, and transcriptomics were performed blinded to genotype and treatment, and data analysis was performed by a different investigator. In cellular studies, fully blinded analysis was not performed because the same person carried out experiments and analysis.

DOI [to be added]

Uncropped Western Blots

Fig.2F

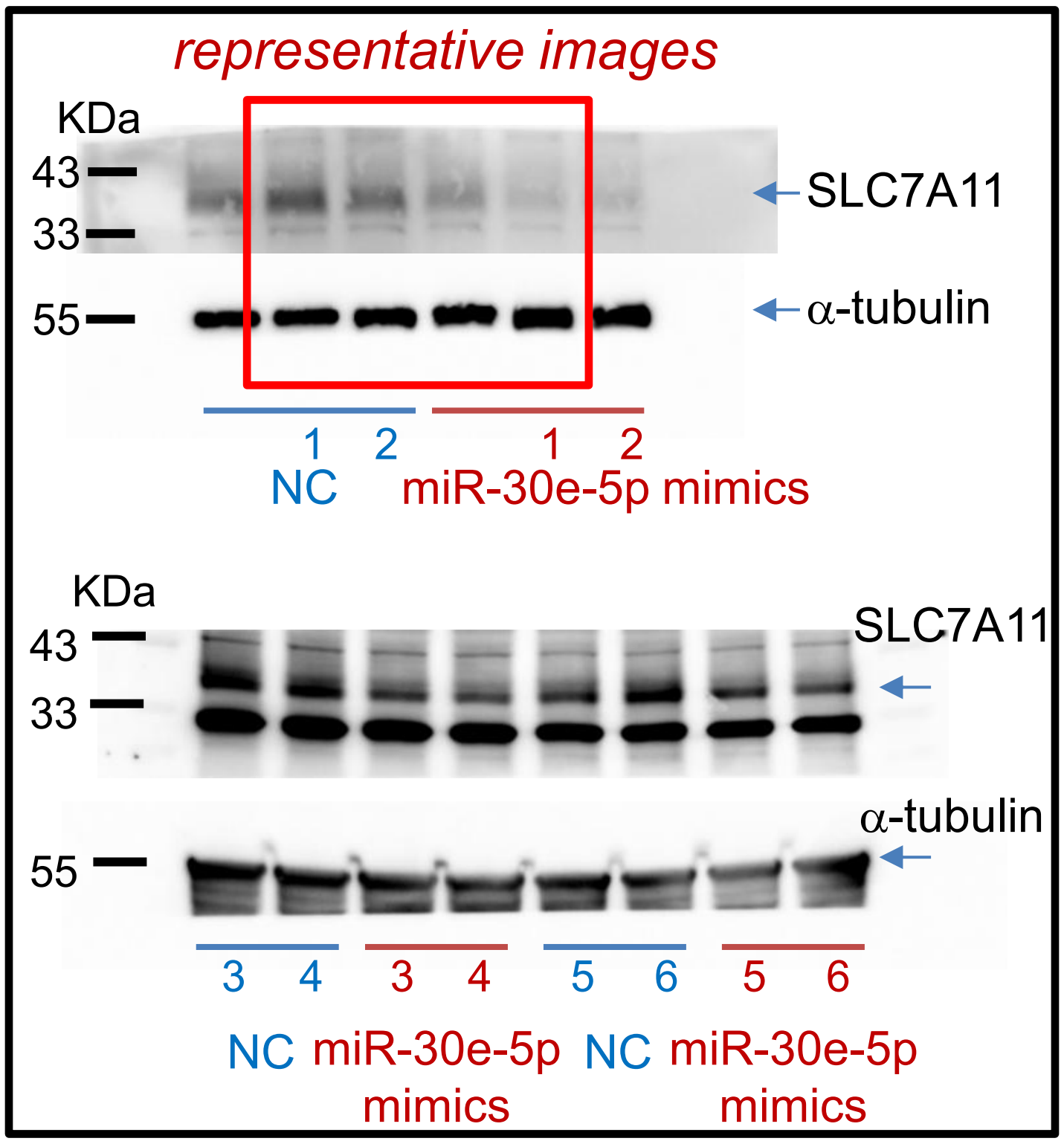

Fig.2G

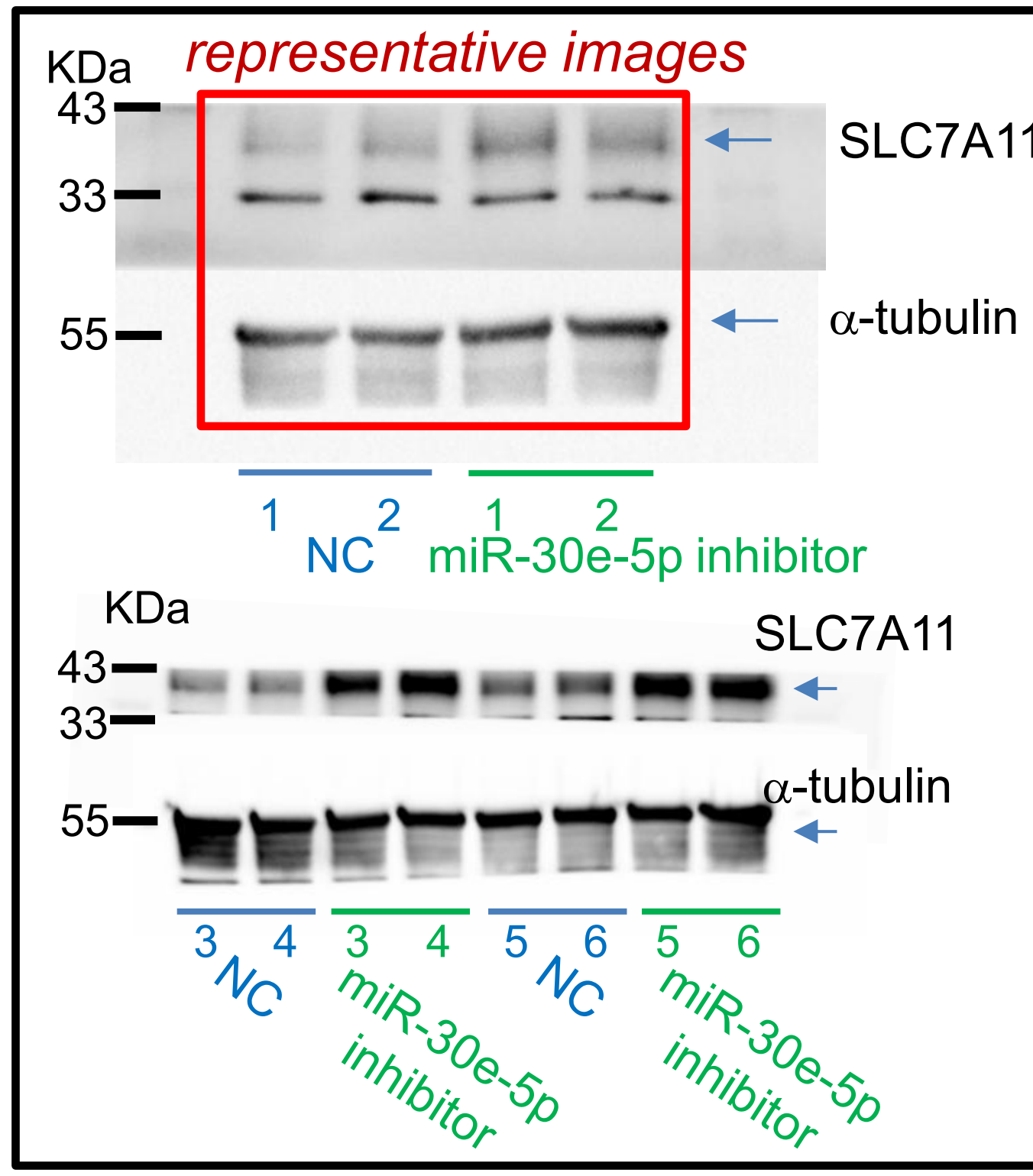

Fig.3F

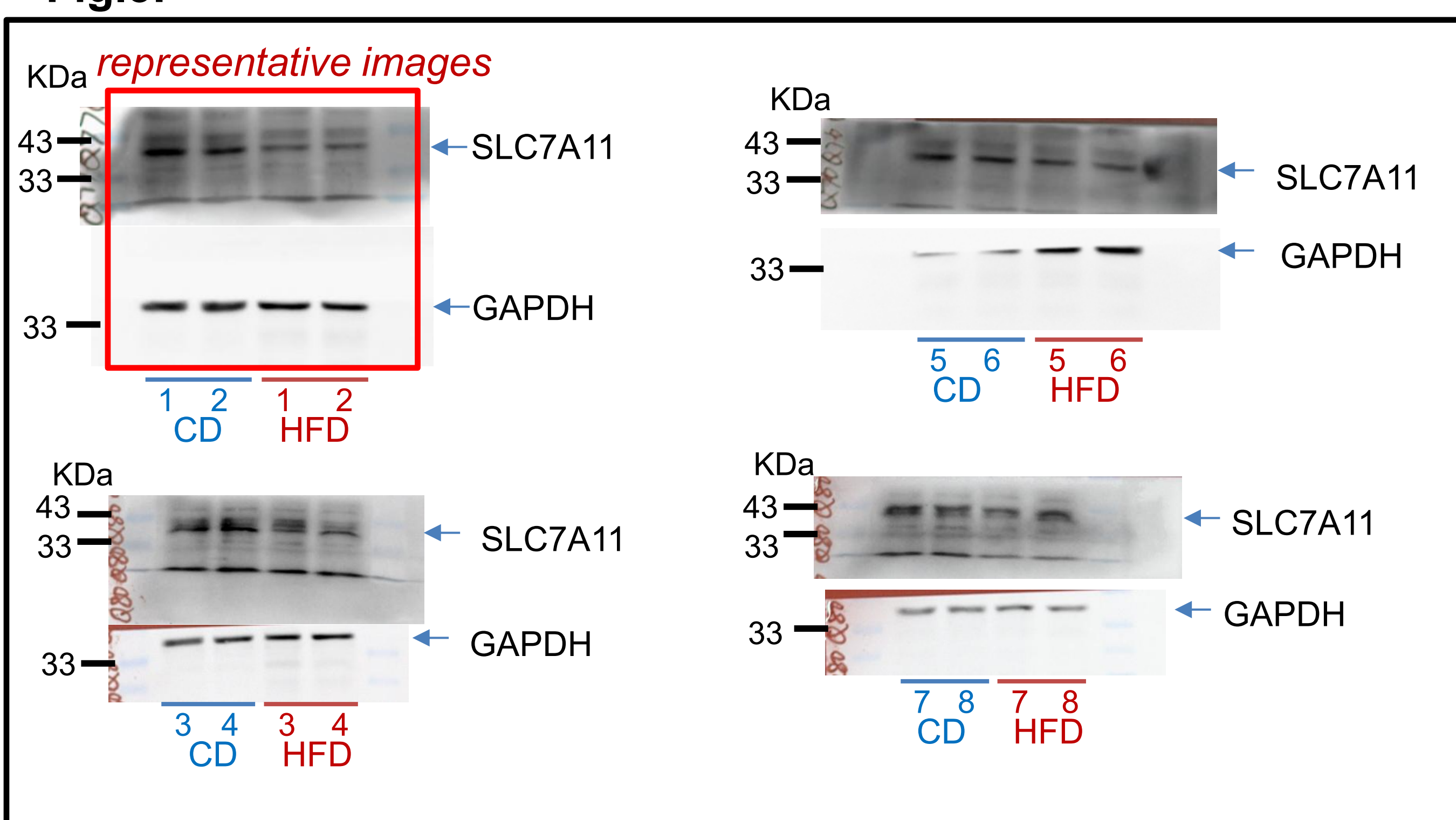

Fig.3G

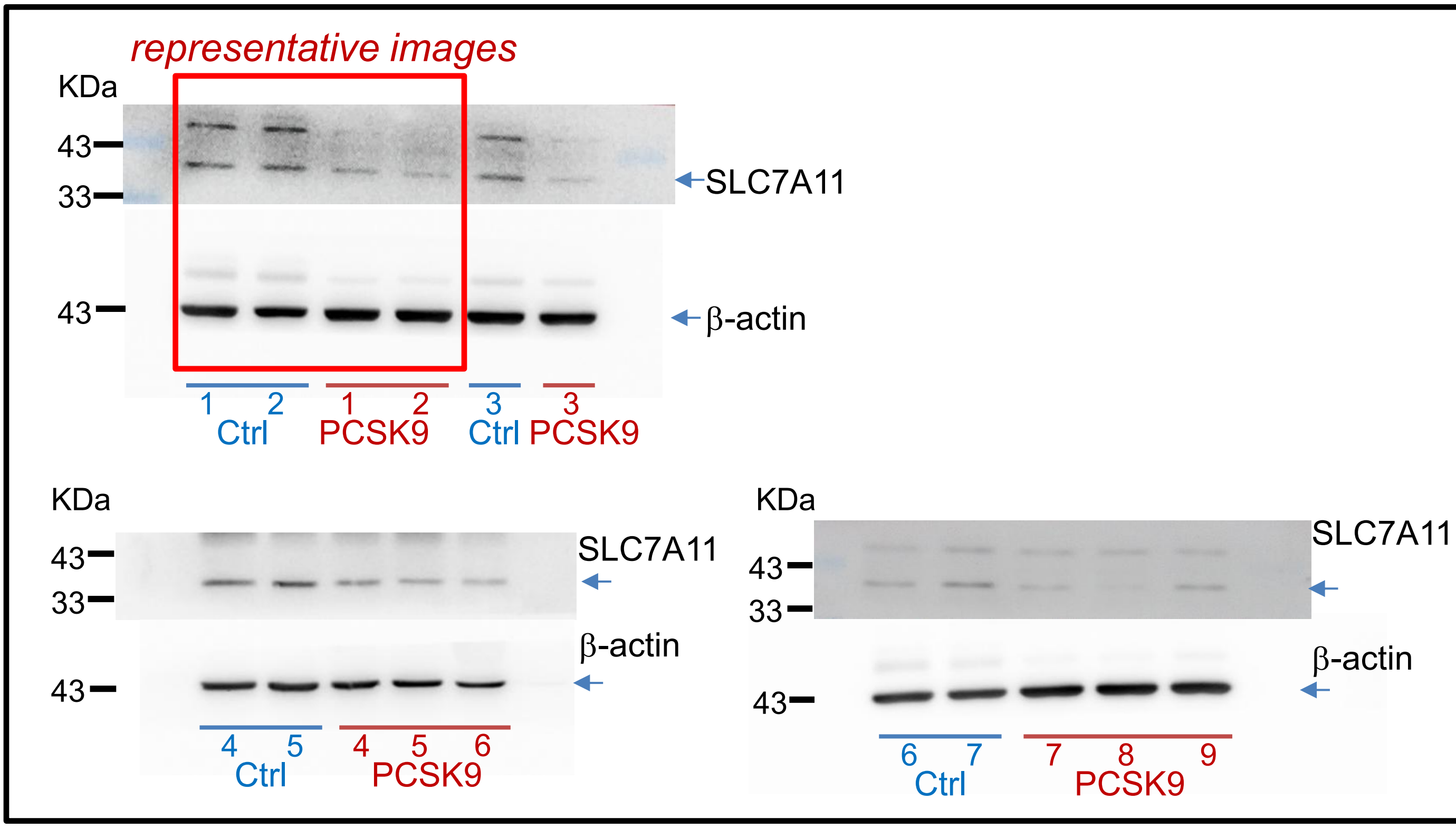

representative images
KDa
43
33
SLC7A11
43
β-actin
1 2 1 2 3 3
Ctrl PCSK9 Ctrl PCSK9
KDa
43
33
SLC7A11
β-actin
43
4 5 4 5 6
Ctrl PCSK9
KDa
SLC7A11
43
33
β-actin
43
6 7 7 8 9
Ctrl PCSK9

Fig.5C

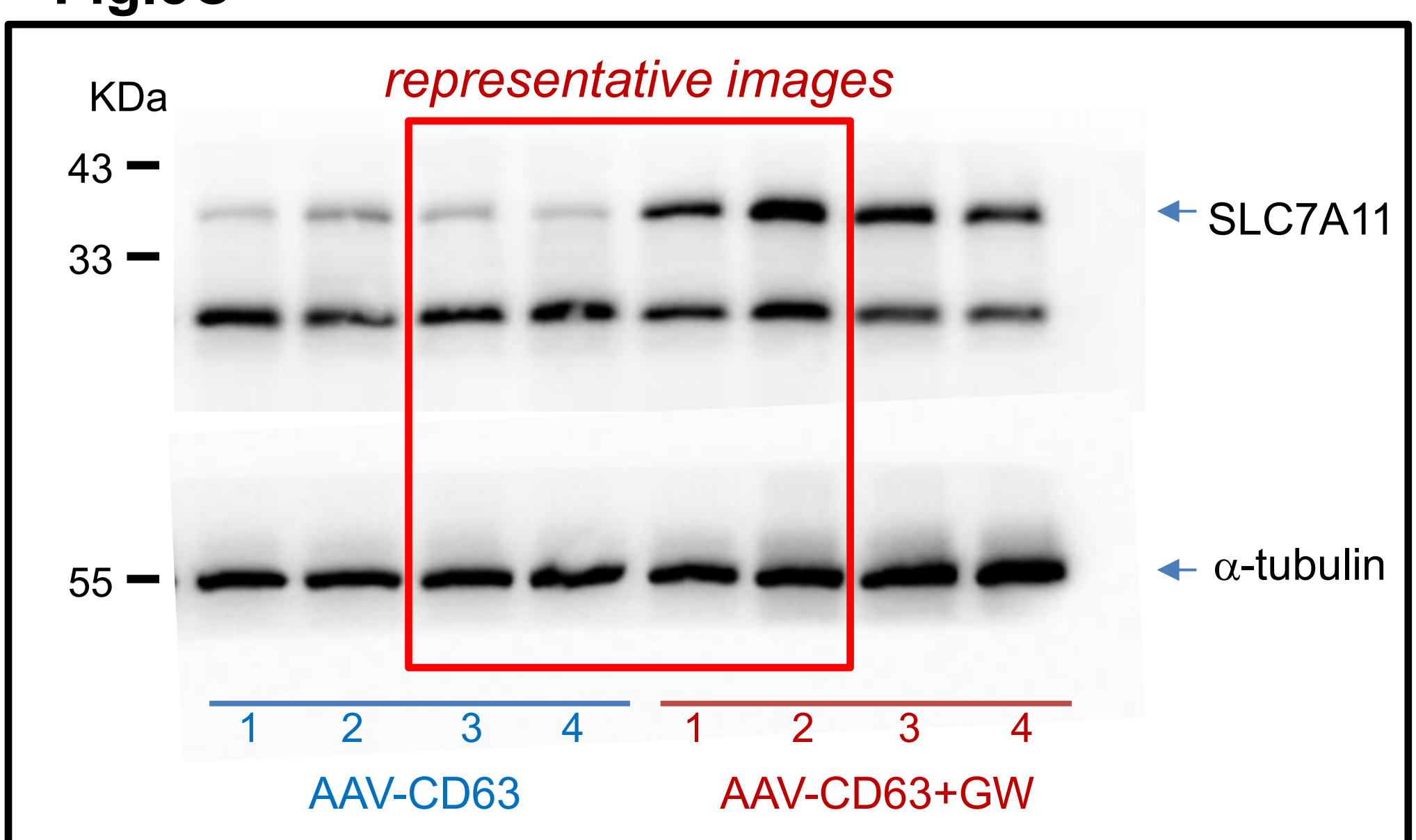

representative images
KDa
43
33
SLC7A11
55
α-tubulin
1 2 3 4 1 2 3 4
AAV-CD63 AAV-CD63+GW

Fig.5L

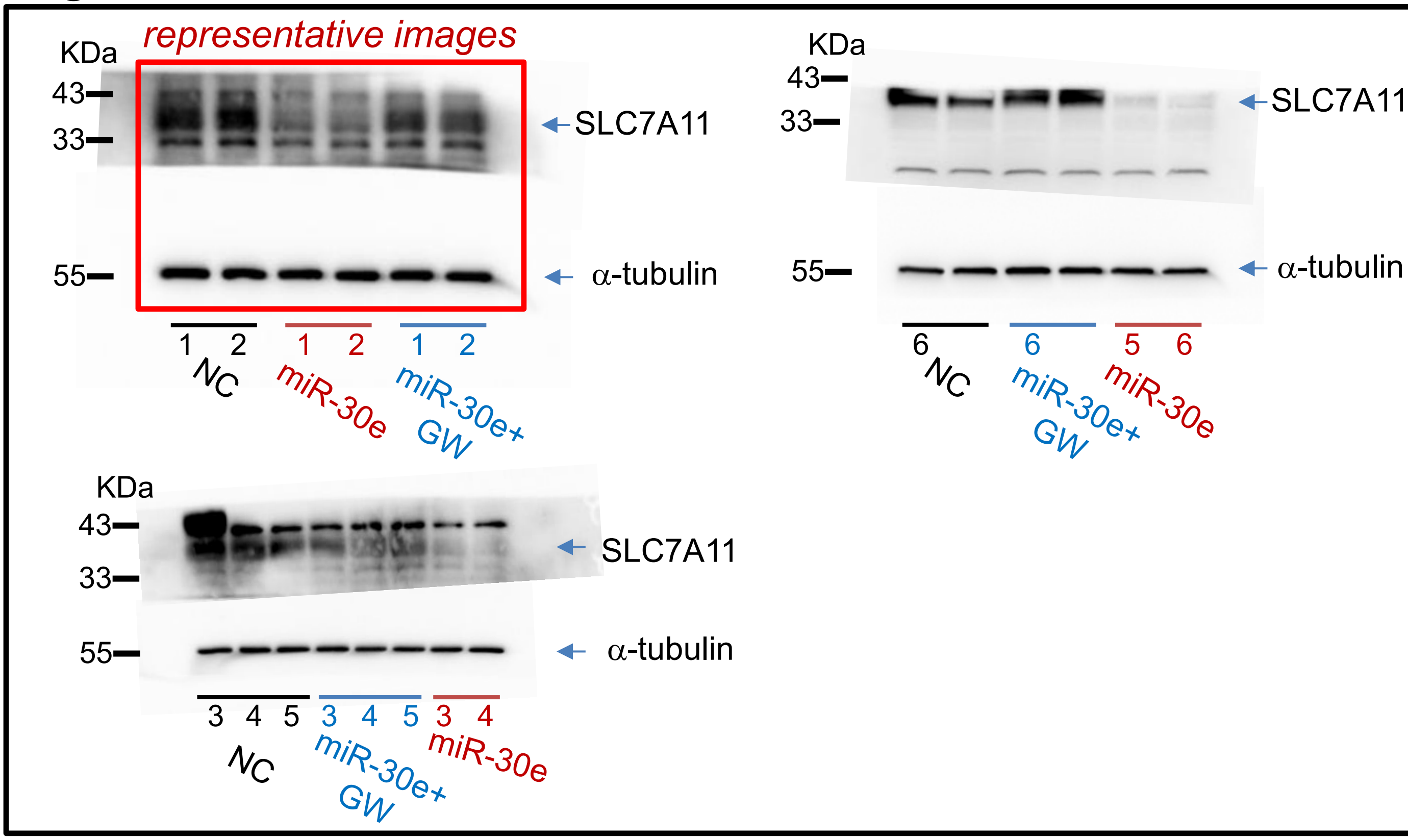
representative images
KDa
43
33
55
SLC7A11
α-tubulin
1 2
NC
1 2
miR-30e
1 2
miR-30e+ GW
KDa
43
33
55
SLC7A11
α-tubulin
6
NC
6
miR-30e+ GW
5 6
miR-30e
KDa
43
33
55
SLC7A11
α-tubulin
3 4 5
NC
3 4 5
miR-30e+ GW
3 4
miR-30e

Fig.6I

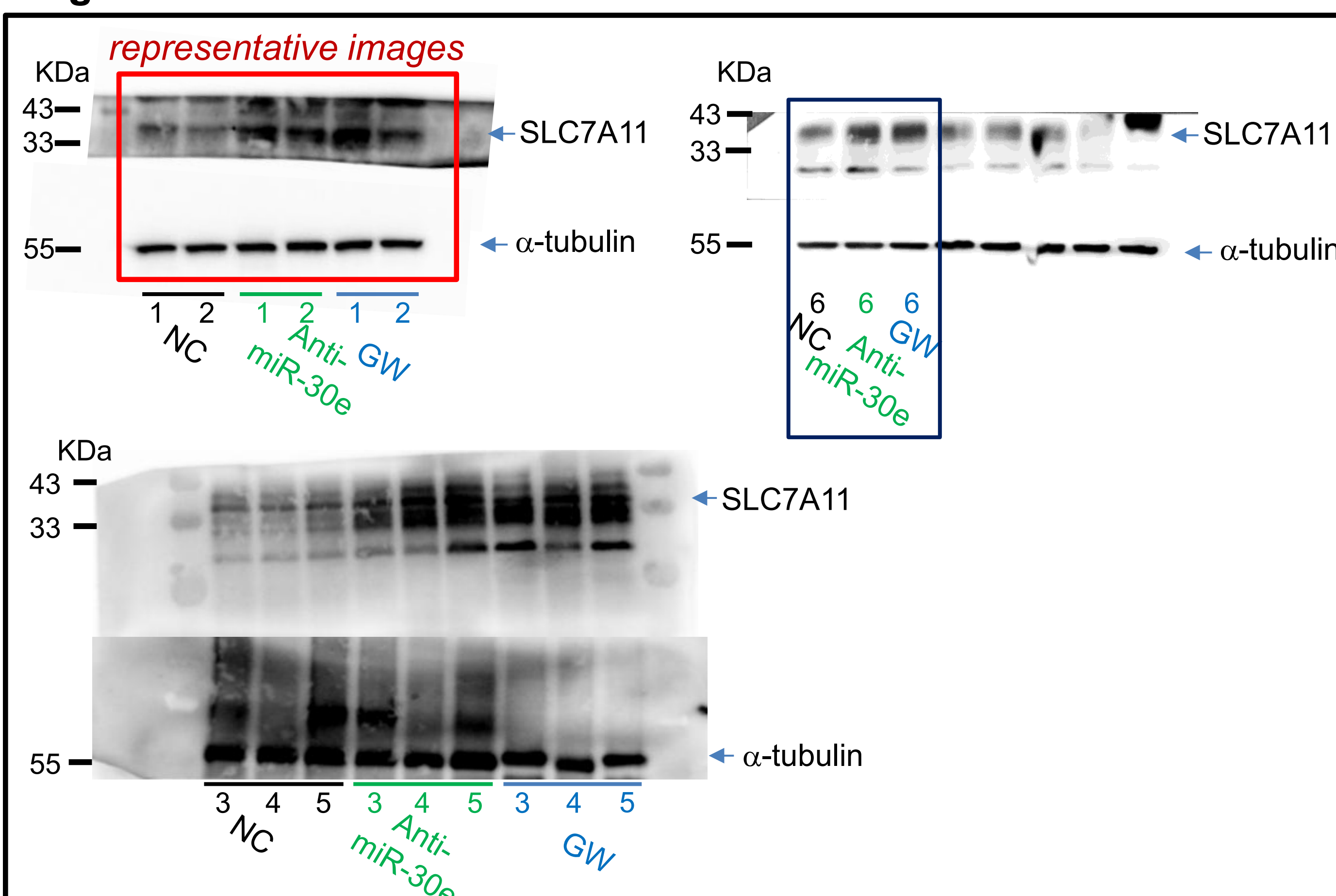
representative images
KDa
43
33
55
SLC7A11
α-tubulin
1 2
NC
1 2
Anti- miR-30e
1 2
GW
KDa
43
33
55
SLC7A11
α-tubulin
6
NC
6
Anti- miR-30e
6
GW
KDa
43
33
55
SLC7A11
α-tubulin
3 4 5
NC
3 4 5
Anti- miR-30e
3 4 5
GW

Fig.S4H

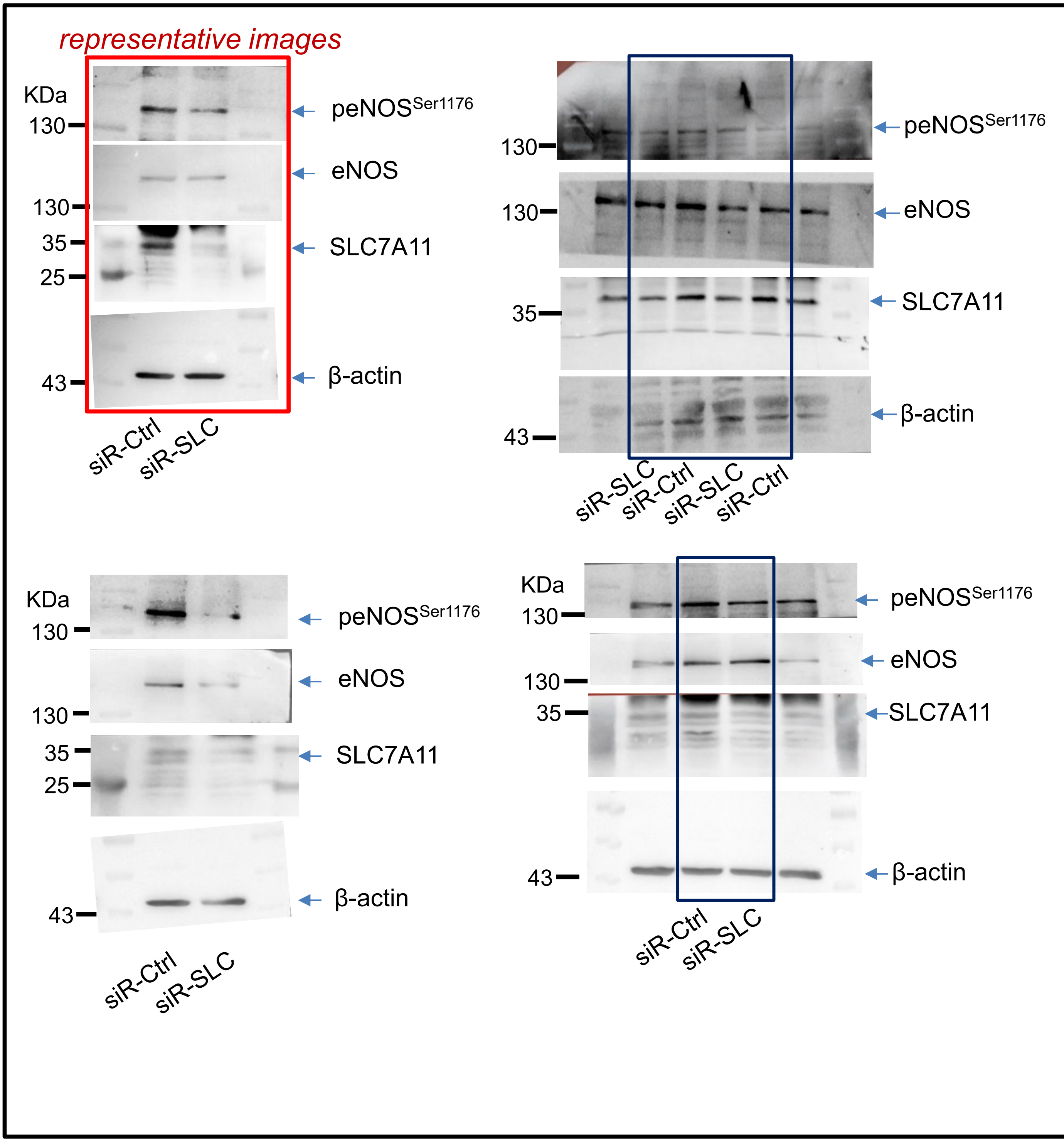
representative images
KDa
130
130
35
25
43
peNOSSer1176
eNOS
SLC7A11
β-actin
siR-Ctrl
siR-SLC
130
130
35
43
peNOSSer1176
eNOS
SLC7A11
β-actin
siR-SLC
siR-Ctrl
siR-SLC
siR-Ctrl
KDa
130
130
35
25
43
peNOSSer1176
eNOS
SLC7A11
β-actin
siR-Ctrl
siR-SLC
KDa
130
130
35
43
peNOSSer1176
eNOS
SLC7A11
β-actin
siR-Ctrl
siR-SLC

Fig.S5A

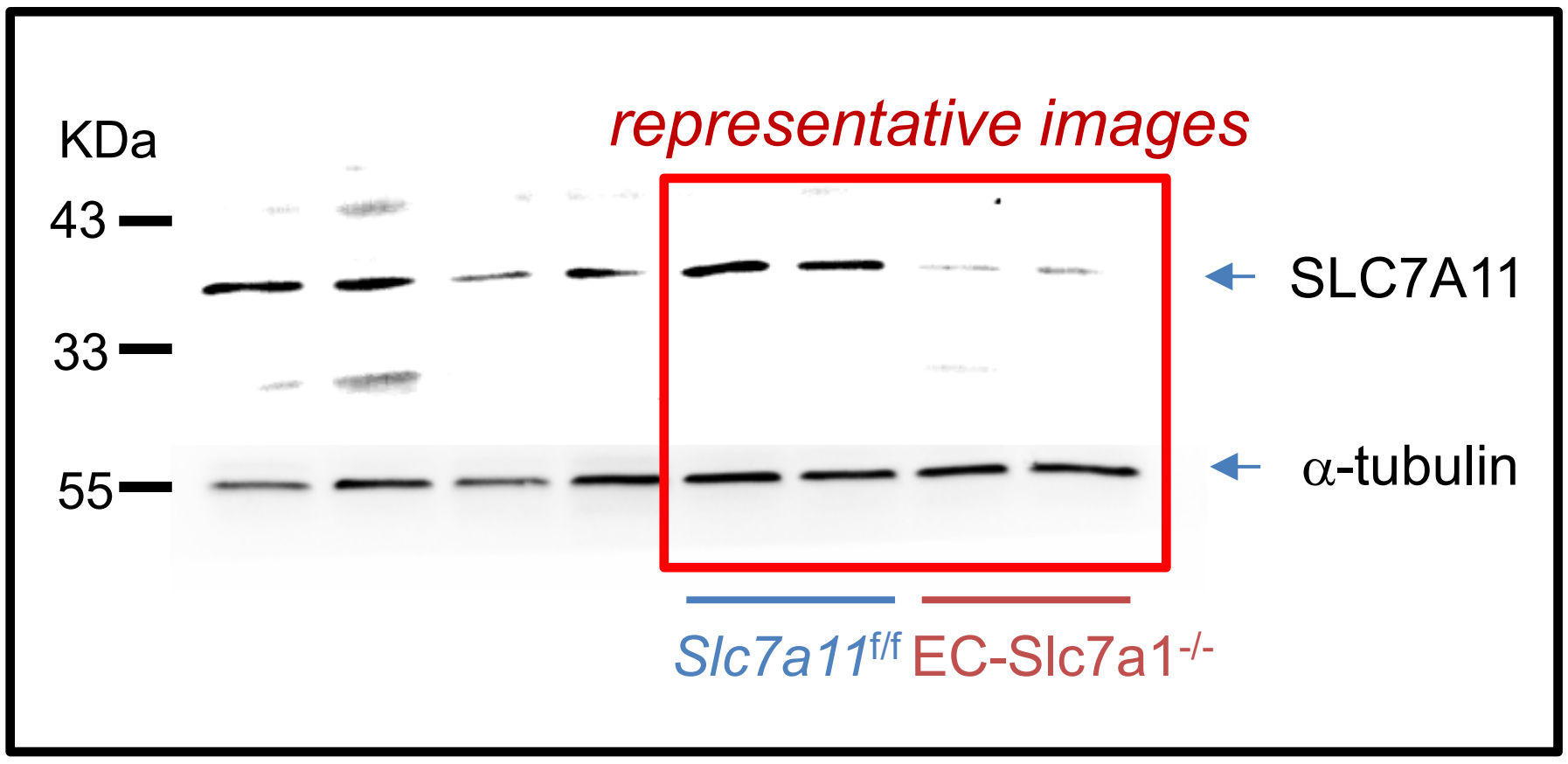
KDa
representative images
43
33
55
SLC7A11
α-tubulin
Slc7a11f/f
EC-Slc7a1-/-
